# Uni‐Axial Densification of Slurry‐Casted Li₆PS₅Cl Tapes: The Role of Particle Size Distribution and Densification Pressure

**DOI:** 10.1002/adma.202501592

**Published:** 2025-05-19

**Authors:** Quoc‐Anh Tran, Meenal Agrawal, Michael Häusler, Johannes Hörmann, Mohsen Sadeqi Moqadam, Günther J. Redhammer, Ingeborg Sellæg Ellingsen, Mir Mehraj Ud Din, Per Erik Vullum, Roman Zettl, Timo Danner, Arnulf Latz, Volker Hennige, Roland Brunner, Daniel Rettenwander

**Affiliations:** ^1^ Department of Materials Science and Engineering NTNU Norwegian University of Science and Technology Trondheim 7034 Norway; ^2^ Christian Doppler Laboratory for Solid‐State Batteries NTNU Norwegian University of Science and Technology Trondheim 7034 Norway; ^3^ Materials Center Leoben Forschung GmbH Leoben 8700 Austria; ^4^ German Aerospace Center (DLR) Institute of Engineering Thermodynamics 89081 Stuttgart Germany; ^5^ Helmholtz Institute Ulm for Electrochemical Energy Storage (HIU) 89081 Ulm Germany; ^6^ Department of Chemistry and Physics of Materials University of Salzburg Salzburg 5020 Austria; ^7^ Department of Physics NTNU Norwegian University of Science and Technology Trondheim 7034 Norway; ^8^ Sintef Industry Trondheim 7491 Norway; ^9^ AVL List GmbH Graz 8020 Austria; ^10^ Institute of Electrochemistry Ulm University 89081 Ulm Germany; ^11^ AIT Austrian Institute of Technology GmbH Center for Transport Technologies Battery Technologies Vienna 1210 Austria

**Keywords:** densification, Li₆PS₅Cl, particle size distribution, solid‐state battery, tape casting

## Abstract

Solid‐state batteries are transformative solutions for electric vehicles, offering superior energy density and safety. Sulfide‐based solid electrolytes like Li₆PS₅Cl (LPSCl) combine high ionic conductivity and mechanical adaptability, but challenges remain in scaling up high‐performance separator tapes due to particle size distribution (PSD) and processing constraints. This study investigates the uni‐axial densification of slurry‐casted LPSCl tapes, focusing on PSD refinement and compaction pressure. Wet milling has been identified to effectively reduce PSD to submicron levels while preserving structural integrity and near‐pristine conductivity. A critical pressure threshold (≈350 MPa) for tape‐casted LPSCl slurries (2.5% hydrated poly(acrylonitrile‐co‐butadiene)) is identified, where ionic conductivity peaks due to particle fusion and the formation of conductive networks. However, open porosity (≈30%), particularly along the densification direction, and surface irregularities persist. These structural issues have significant implications for battery performance. For example, surface roughness and interfacial voids lead to localized current focusing, with current densities exceeding applied values by over 20 times. Percolating porosity accelerates dendritic failure modes, undermining stability and limiting cycling rates. This work underscores the need for optimized powder processing and densification techniques to enhance scalability and performance, advancing LPSCl‐based separators for the practical adoption of solid‐state batteries in electric vehicles and other high‐energy applications.

## Graphical abstract

Solid‐state batteries promise high energy density and safety for electric vehicles. This study examines the densification of slurry‐casted Li₆PS₅Cl separator tapes, highlighting the role of particle size distribution and uni‐axial pressure. Wet milling refines particles while preserving conductivity, and a critical compaction threshold leads to superior performance. Structural defects, however, impact cycling stability, underscoring processing optimization needs.

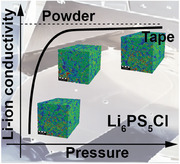

## 1 Introduction

Solid‐state batteries (SSBs) are emerging as a transformative technology for electric vehicles (EVs), offering the promise of higher energy densities and improved safety compared to conventional lithium‐ion batteries. Recent studies have demonstrated that SSBs in pouch cell formats can achieve energy densities exceeding 270 Wh kg^−1^ and 650 Wh L^−1^ sparking intense research into optimizing their components.^[^ ^1^ ^]^ Central to their performance are inorganic solid electrolytes (SEs), such as Li_7_La_3_Zr_2_O_12_ (LLZO),^[^ ^2^ ^]^ NASICON's (e.g., LiM_2_^4+^(PO_4_)_3, with_ M = Ti, Zr, Hf, etc.),^[^ ^3^ ^]^ thio‐LISICON (i.e., Li₁₀GeP₂S₁₂),^[^ ^4^ ^]^ Li₆PS₅Cl (LPSCl),^[^ ^5^ ^]^ and binary Li₂S‐P₂S₅,^[^ ^6^ ^]^ which provide the ionic conductivity necessary for battery operation while addressing the limitations of liquid electrolytes. Among those SEs, sulfide‐based SEs such as LPSCl stands out due to its high ionic conductivity (>10⁻^3^ S cm^−1^ at room temperature) in combination with mechanical adaptability enabling superior interfacial contact with both anode materials and ceramic cathodes. These properties have driven efforts by companies such as SolidPower to commercialize SSBs employing sulfide‐based SEs. Despite their remarkable properties, LPSCl‐based SSBs have predominantly been studied in configurations employing compacted SE powders with thicknesses exceeding 200 µm.^[^ ^7^ ^]^ While these lab‐scale cells demonstrate promising performance, translating such results to the pouch cell format remains a challenge. One critical barrier is the integration of binders and the subsequent processing of, for instance, SE tapes (e.g., tape casting, drying), which are necessary for scalable manufacturing but can hinder ionic conductivity.^[^ ^7^, ^8^ ^]^

SE tapes, which can be also considered as composite electrolytes, require the establishment of a well‐connected percolating network of SE particles. In oxide‐based SE tapes, achieving such conductivity has been proven to be challenging due to high interparticle resistance.^[^ ^9^ ^]^ To achieve high conductivity in such tapes a sinter step at temperatures above 700 °C is required.^[^ ^10^ ^]^ Consequently, the Li‐ion conductivity of these SE tapes often falls significantly below theoretical expectations. Sulfide‐based SEs, by contrast, exhibit a unique capacity for plastic deformation under high pressure, enabling the formation of dense, high‐conductivity SEs by “room‐temperature pressure sintering”.^[^ ^11^ ^]^ This room‐temperature pressure sintering behavior might explain the requirement of high compaction pressures—up to 370 MPa—to realize well performing solid‐state pouch cells.^[^ ^12^ ^]^

Beyond favorable mechanical properties, the particle‐size distribution (PSD) and processing conditions of the SE are crucial for forming uniform, dense green tapes—a prerequisite for full densification under pressure, analogous to high‐temperature sintering processes. For LPSCl tapes, particles larger than 20 µm have been shown to be unsuitable; to ensure a uniform layer, the particle size must remain below half the intended thickness of the tape.^[^ ^13^ ^]^ While ball milling is frequently employed to tailor PSD of LPSCl, it often introduces trade‐offs such as partial amorphization and conductivity loss, as observed in extended milling studies.^[^ ^14^ ^]^ For instance, Cronau et al. employed a two‐step milling process in combination with heptane and dibutyl ether, reducing particle sizes from≈20  to≈1 µm.^[^ ^14b^ ^]^ However, extending milling beyond 2 h led to partial amorphization and significant ionic conductivity loss. Similarly, Wang et al. utilized planetary milling in *m*‐xylene with 6 mm zirconium oxide balls, achieving particle size reductions from 10.8 ± 7.7  to 1.5 ± 0.8 µm after 24 h.^[^ ^14c^ ^]^ Prolonged milling improved PSD uniformity but resulted in decreased conductivity and porosity due to smoother particle surfaces and compacted structures, as revealed by scanning electron microscopy (SEM) and X‐ray diffraction (XRD) analysis. Chen et al.^[^ ^14a^ ^]^ targeted the reduction of non‐uniform PSDs in commercial LPSCl powders exceeding 20 µm through wet ball‐milling in *p*‐xylene, successfully achieving particles smaller than 10 µm. However, excessive size reduction introduced structural defects such as cracks during calendaring, increasing the likelihood of short circuits in SSBs. Additionally, insufficient milling times or oversized grinding media led to inconsistent PSDs, adversely impacting the uniformity of LPSCl tapes.^[^ ^14b^ ^]^ Despite these prior investigations, a comprehensive understanding of how milling parameters influence the PSD, structural integrity, and Li‐ion conductivity remains elusive.

Moreover, the translation of refined LPSCl powders into functional LPSCl tapes introduces further complexities. For instance, Luo, et al. prepared tapes using poly(ethylene oxide) (PEO) as a binder at a 95:5 ratio, achieving a conductivity of 2.83·10⁻⁴  S cm^−1^ at 40 °C, further augmented by the incorporation of LiClO₄ and SiO₂ nanoparticles.^[^ ^15^ ^]^ Similarly, Sedlmeier, et al. reported the development of LPSCl tapes using hydrated poly(acrylonitrile‐co‐butadiene) (HNBR) as a binder at the same 95:5 ratio, with an average particle size of ≈5 µm.^[^ ^16^ ^]^ Conductivity measurements under applied pressure revealed values of 0.17·10⁻^3^ S cm^−1^ at 20 MPa, 0.43·10⁻^3^ S cm^−1^ at 70 MPa, and a maximum of 0.94·10⁻^3^ S cm^−1^ at 590 MPa, highlighting the influence of pressure on ionic transport. Further, Chen et al. employed wet milling to produce LPSCl particles no larger than 10 µm, combining them with 3% to 10% poly(butadiene methacrylate) (PBMA) as a binder.^[^ ^14a^ ^]^ This approach resulted in conductivities ranging from 1.5·10⁻⁴ to 8.6·10⁻⁴ S cm^−1^, underscoring the role of particle size and binder composition in optimizing the electrochemical performance of LPSCl tapes (see Table S1, Supporting Information). Despite considerable progress in processing LPSCl tapes, the effects of integrating SEs with varied PSDs into tapes via slurry‐casting, followed by uniaxial densification, have yet to be systematically investigated.

**Figure 1:**
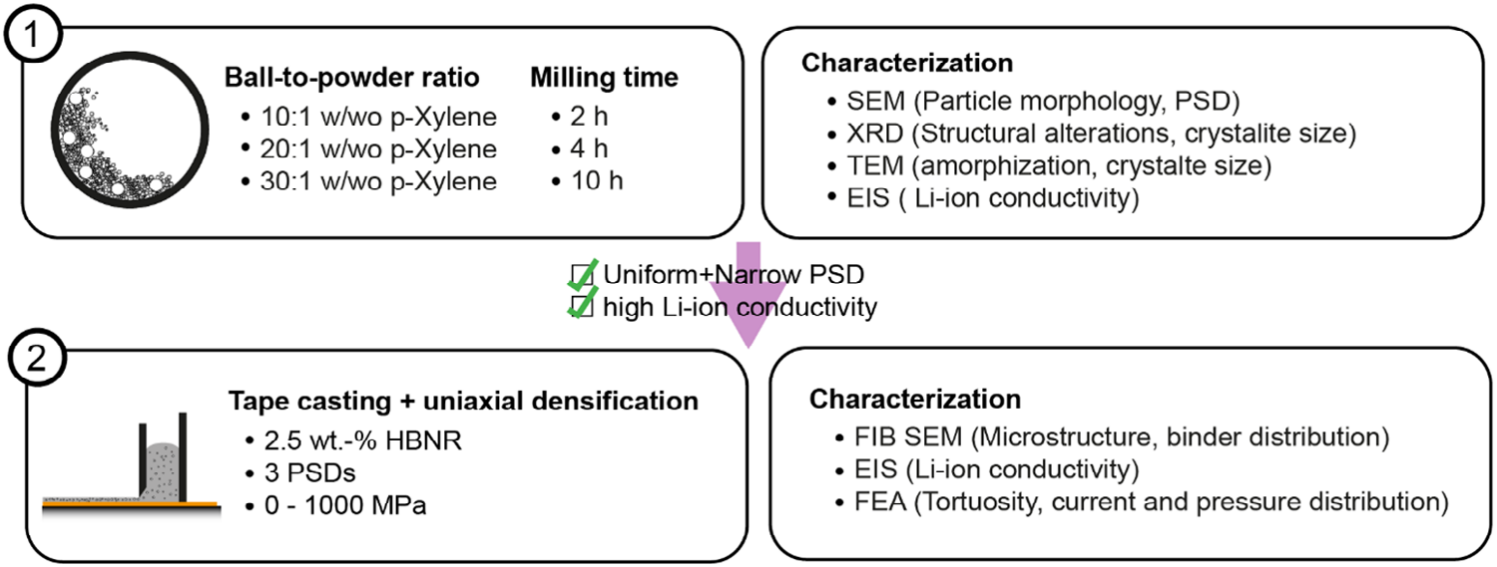
Schematic of the experimental workflow. Step 1: LPSCl powder was processed under eight different milling conditions. High‐energy dry milling with BPRs of 10:1, 20:1, and 30:1 for 10 h, both with and without p‐xylene as a solvent. Additionally, for a BPR of 20:1, milling times of 2, 4, and 10 h were investigated. The resulting samples were characterized using SEM, XRD, TEM, and EIS to evaluate particle morphology, phase composition, and Li‐ion conductivity. Step 2: LPSCl powders with narrow, uniform PSDs and the highest Li‐ion conductivities—specifically those milled at a BPR of 20:1 for 2, 4, and 10 h—were further processed into SE tapes via tape casting. The slurry used for tape casting contained 2.5 wt% HBNR binder. The tapes were subsequently densified under uniaxial pressure ranging from 0 to 1000 MPa and characterized using EIS, FIB‐SEM, and FEM to study their microstructure and transport properties.

In this study, we systematically investigate the impact of milling processes on the PSD, structural integrity, and ionic conductivity of LPSCl powders. Additionally, we examine the densification behavior of LPSCl powders with varying PSDs in LPSCl tapes based on HBNR binder fabricated via tape casting and applying uniaxial pressures of up to 1000 MPa. We further elucidate how the densification of LPSCl tapes influences ionic transport and identify potential implications when integrating these tapes into SSBs (A schematic of the experimental workflow is illustrated in **Figure** **1**).

By employing high‐energy dry milling (HDM) and low‐energy wet milling (LWM), we progressively refine PSDs, optimizing milling times and ball‐to‐powder ratios (BPRs). Characterization via X‐ray diffraction (XRD), transmission electron microscopy (TEM), and electrochemical impedance spectroscopy (EIS) demonstrates that LWM effectively reduces PSD to submicron levels while preserving the material's structural integrity and near‐pristine Li‐ion conductivities. Uniaxial densification studies on LPSCl tapes reveal a critical pressure threshold of ≈350 MPa for maximizing ionic conductivity. Combining insights from EIS, focused ion beam–scanning electron microscopy (FIB‐SEM), and advanced numerical tools for structural, mechanical, and electrochemical analysis, we demonstrate that LPSCl undergoes plastic deformation during densification, forming highly conductive percolating networks with conductivities comparable to compacted powders. However, our findings also show that LPSCl tapes retain a significant pore network, which adversely affects cell performance by increasing the risk of short circuits and causing uneven pressure distributions, thereby limiting the utilization of active materials.

These results highlight the necessity of optimizing both powder refinement and tape fabrication processes to enable the development of high‐performance LPSCl‐based SSBs.

## 2 Results and Discussion

### 2.1 Microstructural Engineering of LPSCl Particles

To reduce and narrow the PSD, we systematically evaluated various milling parameters, including milling duration, BPR, and the incorporation of a solvent medium, on commercially available LPSCl powder (NEI Corporation). As illustrated in Figure S1a,b (Supporting Information), the as‐received LPSCl powder exhibits a highly irregular morphology and a broad PSD, with particle sizes exceeding 30 µm and significant agglomeration.

#### 2.1.1 Implications of Dry Milling on PSD and Structure

The impact of HDM was investigated at 600 rpm using a fixed ball size of 1 mm and a constant BPR of 30:1 (Figure S1c–h, Supporting Information). After 2 h of milling, the average particle size was reduced to 2.42 ± 2.81 µm, although larger particles (>10 µm) persisted. Extending the milling time to 4 h improved particle size uniformity, with a PSD of 2.44 ± 2.15 µm. By 10 h, the PSD further narrowed to 2.6 ± 0.53 µm, effectively eliminating large particles and highlighting the role of extended milling in enhancing particle homogeneity.

**Figure 2:**
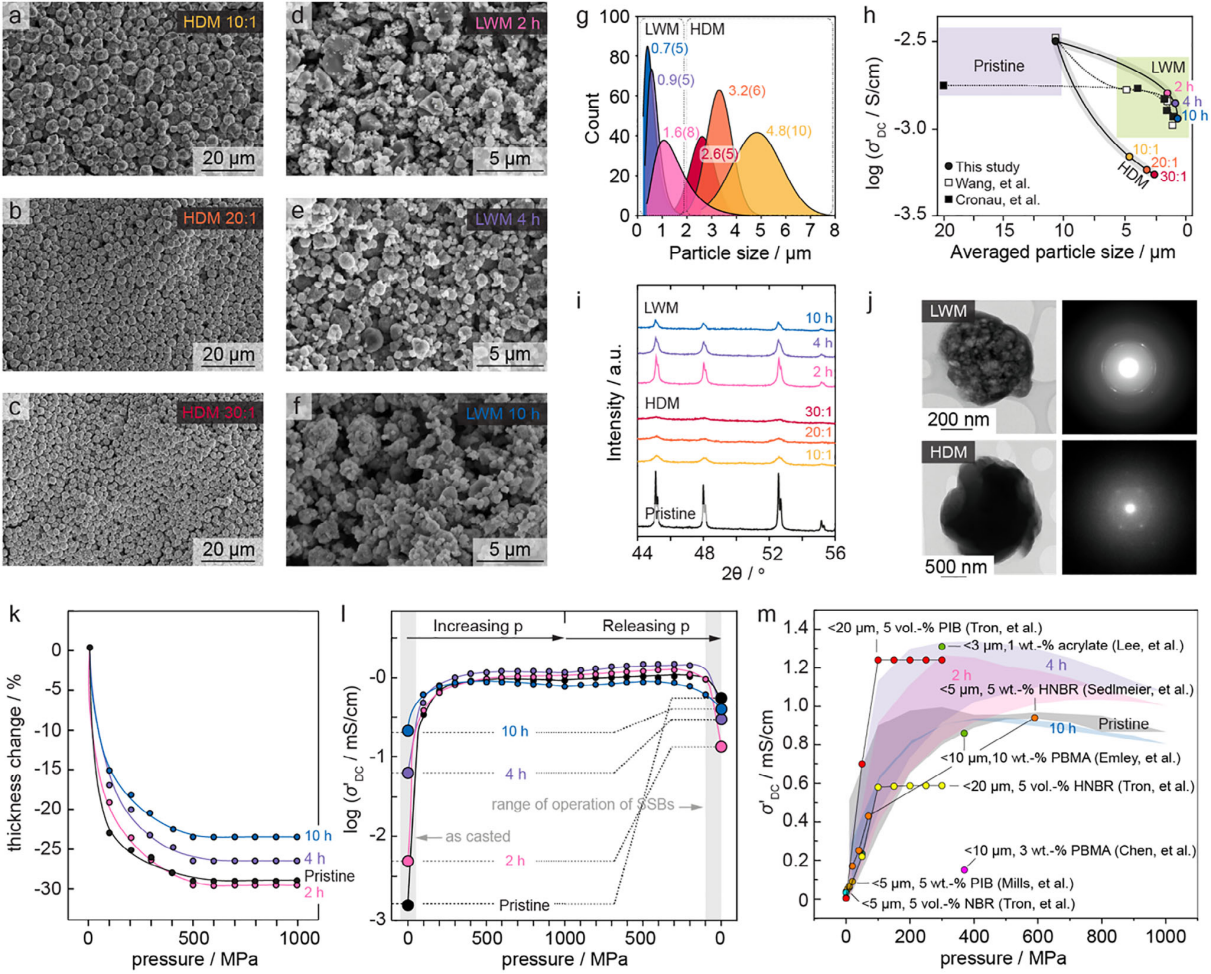
a–c) SEM images of LPSCl powder after HDM at 600 rpm for 10 h with BPR of 10:1, 20:1, and 30:1. d–f) SEM images of LPSCl powder after LWM at 200 rpm with a BPR of 20:1 for milling durations of 2, 4, and 10 h. g) PSD profiles for powders subjected to HDM and LWM. h) Relationship between average particle size and Li‐ion conductivity for different milling conditions with comparisons to the findings of Cronau et al.^[^ ^14b^ ^]^ and Wang et al.^[^ ^14c^ ^]^ i) XRD profiles showing structural evolution of LPSCl powders under varying milling parameters. j) TEM images and SAED patterns comparing LWM (top) and HDM (bottom) powders milled for 10 h with a BPR of 20:1, highlighting structural differences. k) Thickness reduction of LPSCl tapes as a function of uniaxial pressure during densification. l) Li‐ion conductivity of LPSCl tapes as a function of uniaxial densification pressure. m) Comparative analysis of uniaxial densification studies from the literature^[^ ^13^, ^14^, ^16^, ^21^, ^22^ ^]^ with results from this work.

To further refine the PSD, BPR values were adjusted to 10:1 (HDM_10:1), 20:1 (HDM_20:1), and 30:1 (HDM_30:1), with a constant milling time of 10 h. SEM images of HDM particles with different BPRs (**Figure** **2**a–c) reveal uniform PSDs (Figure 2g), with average particle sizes of 4.8 ± 1.08 , 3.2 ± 0.6 , and 2.6 ± 0.53 µm for HDM_10:1, HDM_20:1, and HDM_30:1, respectively.

The effects of these dry milling conditions on Li‐ion conductivity were examined using EIS of powder pressed pellets of LPSCl powders (cf Figure S2, Supporting Information) showed a significant decline from 3.08·10⁻^3^ S cm^−1^ in pristine samples to 0.57·10⁻^3^, 0.44·10⁻^3^, and 0.39·10⁻^3^ S cm^−1^ for increasing BPRs (Figure 2h), implying a performance loss that could be problematic for catholyte applications where conductivities near 10^−2^ S cm^−1^ are desirable.^[^ ^17^ ^]^ This decline in conductivity is likely due to structural degradation and partial amorphization from high‐energy impacts during dry milling. Powder XRD pattern reveals a broadening of diffraction peaks in correlation with BPR increases (Figure 2i). The presence of larger particles (>1 µm) suggests, however, that peak broadening could be attributable to structural degradation and amorphization rather than crystallite fracturing. To clarify peak broadening origins, TEM and selected area electron diffraction (SAED) were conducted on representative LPSCl particles. A bright field TEM image and a SAED pattern from one of the particles are shown at the bottom in Figure 1j. Diffraction patterns from this sample predominantly gave an amorphous signal, but with some residual crystallinity. Weak Bragg reflections from a single crystal are evident in the electron diffraction pattern shown in Figure 1j, but most of the particle is amorphous. As crystallite sizes remained unchanged, ion conductivity reductions associated with higher resistive grain boundaries are ruled out, supporting the hypothesis that structural degradation underlies the Li‐ion conductivity loss.

#### 2.1.2 Implications of Wet‐Milling on PSD and Structure

A solvent medium was then explored to further reduce particle size while mitigating structural degradation. Solvent milling offers several advantages, such as efficient heat dissipation, minimized particle agglomeration, and a narrower PSD with lower energy input. Among various solvent choices like heptane or toluene, p‐xylene was a balance between dispersion stability and low reactivity with LPSCl and its effectiveness in preventing excessive fragmentation while preserving crystallinity.^[^ ^18^ ^]^ Wet‐milling was performed at 200 rpm using different BPR values, i.e., 10:1, 20:1, and 30:1, with a constant milling time of 2 h. SEM images highlight the efficiency of LWM in achieving narrow PSDs in short duration, with no large particles (>10 µm) were observed in any of the applied conditions (Figure S3, Supporting Information). The average particle sizes were reduced to 1.8 ± 1.10 µm, 1.6 ± 0.88 µm, and 1.1 ± 0.69 µm for LWM_10:1_2 h, LWM_20:1_2 h, and LWM_30:1_2 h, respectively. This indicates that different BPR values can effectively tailor PSDs, similar to the trends observed in HDM. However, unlike HDM, where large particles persisted under comparable short milling durations (2 h), LWM demonstrated superior capability in reducing particle size and achieving greater uniformity.

To further reduce particle size and achieve a narrower PSD, milling duration was extended to 4 and 10 h under a 20:1 BPR condition. SEM images of LWM particles with different milling time (Figure 2d–f) reveal that the PSD narrowed significantly with increasing time: from 1.6 ± 0.88 µm after 2 h (LWM_2 h) to 0.9 ± 0.48 µm after 4 h (LWM_4 h), and 0.7 ± 0.44 µm after 10 h (LWM_10 h) (Figure 2g), with LWM outperforming HDM in particle size reduction and PSD narrowing under identical BPR conditions (20:1). The Li‐ion conductivity of wet‐milled LPSCl powders, decreased as the particle size was reduced (cf Figure S2, Supporting Information). LWM_2 h (1.6 ± 0.88 µm) exhibited a conductivity of 2.2·10⁻^3^ S cm^−1^, LWM_4 h (0.9 ± 0.48 µm) achieved 1.9·10⁻^3^ S cm^−1^, and LWM_10 h (0.7 ± 0.44 µm) reached 1.5·10⁻^3^ S cm^−1^. Despite the decline with extended milling, these values remained significantly higher than those of HDM counterparts: HDM_10:1 (4.8 ± 1.08 µm) recorded 0.57·10⁻^3^ S cm^−1^, HDM_20:1 (3.2 ± 0.6 µm) achieved 0.44·10⁻^3^ S cm^−1^, and HDM_30:1 reached 0.40·10⁻^3^ S cm^−1^. We hypothesize that the significant difference in conductivities between LWM and HDM is attributed to the lower and more homogeneous energy impact of the wet‐milling process. This leads to the fracturing of crystallites rather than amorphization, which is typically observed in HDM. Tables S2, S3 (Supporting Information) summarize the total energy impact under each milling condition. This hypothesis is further supported by additional XRD measurements incorporating an internal standard. Each sample was mixed with 50 wt.% of high‐purity crystalline silicon powder, whose constant peak intensity served as a normalization reference. This enabled accurate quantification of the relative loss in LPSCl crystallinity through Rietveld refinement (Table S4, Supporting Information). A gradual increase in amorphous content is observed with extended milling time in LWM: 0.2% in pristine LPSCl, 2.7% (LWM_2 h), 5.1% (LWM_4 h), and 8.6% (LWM_10 h). In contrast, HDM induces significantly higher amorphization: 25.5% (HDM_10:1), 29.7% (HDM_20:1), and 29.2% (HDM_30:1). Interestingly, although HDM_30:1 leads to a smaller particle size than HDM_20:1, the amorphous content remains nearly unchanged. This suggests that crystallinity loss reaches a saturation point for the applied milling conditions. These observations are consistent with prior studies on wet‐milled LPSCl (Figure 2h), while Cronau et al. associated the decreasing conductivity with amorphization (reflected in diffraction peak broadening),^[^ ^14b^ ^]^ Wang et al. observed no XRD changes.^[^ ^14c^ ^]^ Similar to Cronau et al., Chen et al.^[^ ^14a^ ^]^ noted peak broadening but attributed it to crystallite size reduction. Our findings align with Chen et al., as TEM analysis (Figure 2j) showed fracturing of crystallites into nanometer‐sized domains causing peak broadening per Scherrer's equation. The polycrystalline nature was further confirmed by SAED, with diffraction rings replacing spotty patterns, reinforcing the association between PSD shifts to sub‐µm regions and peak broadening.

### 2.2 Processing of LPSCl Particles into Tapes

Achieving high Li‐ion conductivities in LPSCl tapes requires optimizing both the intrinsic conductivity of the particles and their effective percolation within the composite matrix. This involves precise control over particle distribution, pore structures, and binder content to ensure efficient interparticle contact. Unlike oxide‐based SEs such as LLZO, which often require sintering to reduce interparticle resistance,^[^ ^9^ ^]^ sulfide‐based SEs like LPSCl can densify under applied pressure without sintering.^[^ ^19^ ^]^ This room‐temperature pressure sintering process^[^ ^20^ ^]^ plays a crucial role in tape densification. In the following sections, we first examine the impact of uniaxial pressure on electrochemical performance by correlating Li‐ion conductivity with microstructural features such as porosity and tortuosity. We then explore how porosity and surface roughness influence current and stress distribution at the electrode|electrolyte interface, particularly in relation to Li‐dendrite formation and propagation.

#### 2.2.1 Impact of Uni‐Axial Pressure on Tape Densification and Li‐Ion Transport Properties

The impact of uniaxial pressure on tape densification and Li‐ion transport properties was investigated through pressure‐dependent impedance spectroscopy conducted over a pressure cycle, ranging from 0 to 1000 MPa and back to 0 MPa (see Figure S4, Supporting Information) with corresponding thickness data collected in 100 MPa increments. Note, given their superior performance compared to particles produced through HDM, only LPSCl particles processed via LWM were used for LPSCl tape fabrication.

Under uniaxial pressure, all LPSCl tapes exhibited a thickness reduction of ≈23%–30%, with maximum densification achieved at≈350 MPa (Figure 2k). This densification behavior is similar to that observed during a conventional sintering process, progressing from the green body density to the final sintered state typical of ceramic materials. Notably, tapes fabricated with smaller and narrower PSDs displayed reduced thickness changes under pressure, likely due to their higher initial green tape density. This enhanced initial compaction correlates with the higher Li‐ion conductivities observed in tapes made from smaller LPSCl particles (Figure 2l). As tape thickness reduction approached its limit, corresponding to the densification plateau, Li‐ion conductivity also stabilized at values comparable to those of compacted LPSCl powders.

**Figure 3:**
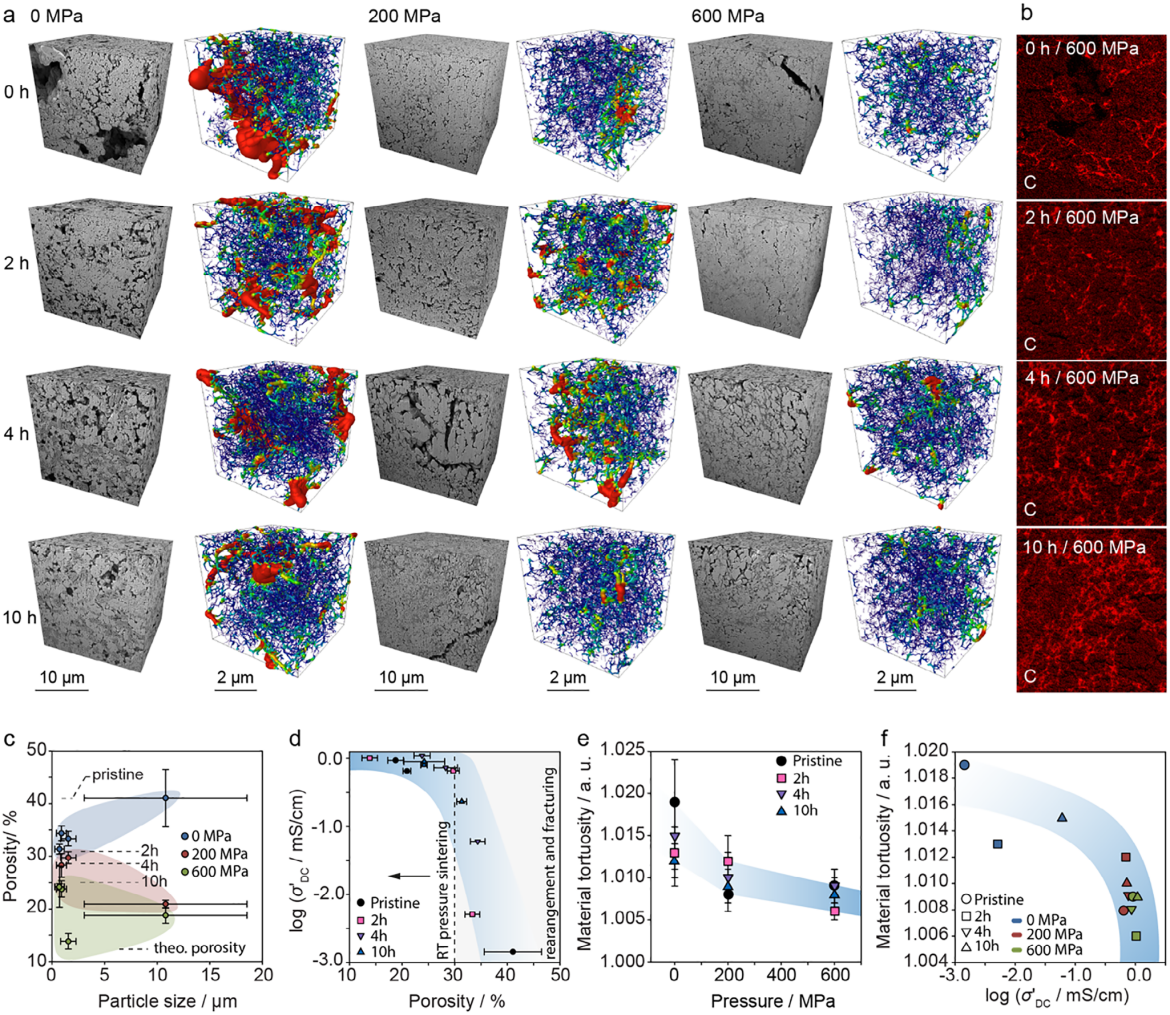
a) FIB‐SEM tomography of SE tapes prepared via pristine and LWM for 2, 4, and 10 h under densification pressures of 0 , 200 , and 600 MPa. 3D reconstructions from backscattered electron images and pore phase skeletonization illustrate the microstructure and inter‐grain phase distribution. b) EDS mapping of carbon (red) visualizing binder distribution in SE tapes densified at 600 MPa, highlighting spatial variations. c) Correlation between porosity and particle size for tapes processed under different conditions. The dashed line represents the theoretical green body density based on PSD and the Andersen relation, aligning with experimental values. Increasing densification pressure generally reduces porosity, though no systematic trend is observed with PSD. d) Li‐ion conductivity as a function of porosity, indicating that tortuosity has a minor impact on conductivity. Beyond a critical pressure threshold, Li‐ion conductivity remains stable within a porosity range of≈15%–35%. e) Material tortuosity as a function of f) densification pressure and Li‐ion conductivity. Tortuosity systematically increases with pressure but has a negligible effect on Li‐ion conductivity. Once densified, Li‐ion conductivity shows no significant change.

To better understand the relationship between densification of LPSCl tapes and Li‐ion transport properties, FIB‐SEM tomography with subsequent image segmentation was performed on representative tapes made from pristine, LWM_2 h, LWM_4 h, and LWM_10 h samples, examining both non‐densified and densified states—before the significant rise in Li‐ion conductivity and within the plateau region where conductivity stabilizes. **Figure** **3**a presents the 3D microstructure along with the extracted skeletal pore structure (cf Figures S5–S7 and Note S1, Supporting Information). The pore‐network skeletonization highlights not only the size and number of pores but also indicates the degree of percolation of pores (see Figure S8, Supporting Information). Note, larger cracks in densified samples are artifacts and will not be considered for further quantification of porosity, tortuosity, etc.

The pristine LPSCl tape exhibits high porosity (ca. 40%) due to irregular particle shapes and sizes, preventing close packing. This microstructure results in a relatively low conductivity of 1.44·10⁻⁶ S cm^−1^, comparable to other SEs in composite form.^[^ ^19^, ^20^ ^]^ Unlike other composite electrolytes where conduction occurs primarily through the polymer, our tapes utilize a non‐conductive binder, precluding polymer‐based conduction. Upon increasing pressure to 100 MPa, a 25.5% reduction in thickness is observed, with a marked conductivity increase to 0.33·10⁻^3^ S cm^−1^. This value aligns closely with previous reports for LPSCl tapes, though slightly lower than those from Tron et al., who used polyisobutylene and pre‐densified their tapes at 370 MPa (Figure 2m).^[^ ^21^ ^]^ The higher Li‐ion conductivity could be explained with the different binder used but cannot explain the lower values obtained for samples using same LPSCl and binder as used herein. Further pressure increases yield a thickness reduction of 32%, raising conductivity to 0.93·10⁻^3^ S cm^−1^ at 300 MPa, exceeding values achieved so far for LPSCl tapes (based on our best knowledge;^[^ ^13^, ^14^, ^16^, ^21^, ^22^ ^]^ see Figure 2m). Beyond this pressure, conductivity plateaus despite additional densification (total reduction: 68%). Reducing pressure to 600 MPa preserves high conductivity, indicating plastic deformation of LPSCl, similar to effects observed for densified LPSCl powders (see above).^[^ ^12^, ^23^ ^]^ Table S5 (Supporting Information) summarizes the values of conductivities of pristine, 2 , 4 , and 10 h milled samples with pressure.

The binder distribution within the LPSCl tape plays a crucial role regard of both the mechanical integrity and Li‐ion conductivity of the tape. To assess binder distribution, energy dispersive spectroscopy (EDS) analysis was conducted on representative cross‐sections selected from FIB‐SEM analysis. Figure 3b shows the resulting binder distribution in representative samples, with the carbon (C) signal serving as a marker for the presence of the HNBR binder. The results show that as the SE particle size decreases and the PSD narrows, the binder becomes more homogeneously distributed throughout the tape. The ability of the binder to homogeneously distribute is related to the low glass transition temperature of the HNBR binder (T_g_ = −24 °C), which remains below the pressing temperature, ensuring sufficient binder distribution within the green body tape.^[^ ^24^ ^]^

A quantitative analysis of the microstructure and connection to the Li‐ion conductivity is shown in Figure 3c–f. In Figure 3c the relation between the PSD and porosity is illustrated. The dashed line corresponds to the theoretical green body density of tapes (f) considering the PSD of the particles used using the Andersen relation given by f = (D – D_min_) / (D_max_ – D_min_), with D, D_min_, and D_max_ as the mean, lower end, and upper end of the PSD, respectively. The calculated f values fit perfectly with the experimental obtained values as indicated with dashed lines in Figure 3c. The quantitative analysis general shows a lower porosity with increasing densification pressure. However, no systematic trend is observed regarding the PSD. This absence of correlation may stem from the identical slurry formulation used across all tape casting processes. Since particle size variations influence rheological behavior—affecting viscosity, dispersion, and flow properties—each PSD likely interacts differently with the slurry, impacting green tape density before densification begins. Additionally, mechanical differences between particle sizes further contribute to inconsistencies: larger particles deform more under pressure, while smaller ones primarily rearrange to enhance packing. As a result, densification is governed by a complex interplay of slurry rheology, initial particle distribution, and mechanical deformation, rather than PSD alone. This highlights the need to optimize slurry formulations for any modification of particles used in the tape fabrication.

Figure 3d represents the plot of Li‐ion conductivity (measured by EIS) against the porosity where, a significant increase in the Li‐ion conductivity can be observed when a porosity of ≈32% has been reached, while remaining constant thereafter. We hypothesize that room‐temperature pressure sintering will take place, when no further densification through the rearrangement of particles or their fracturing can be achieved by applying pressure. While the Li‐ion conductivity is low before densification, fusion of particles at higher pressure will lead do a boost in Li‐ion conductivity by overcoming high interparticle resistances. Any further increase will be related to tortuosity or structural alteration caused by the introduction of structural defects as recently shown by Zeier and co‐workers.^[^ ^25^ ^]^ In Figure 3e,f the relation between the geometric tortuosity, pressure, and Li‐ion conductivity is shown, respectively. A steady decrease in the geometric tortuosity is observed with increasing pressure, while the Li‐ion conductivity initially shows a large impact before stabilizing at a nearly constant level. Again, the initial large change might be related to the high interparticle resistances when particles have not experienced enough pressure.

**Figure 4:**
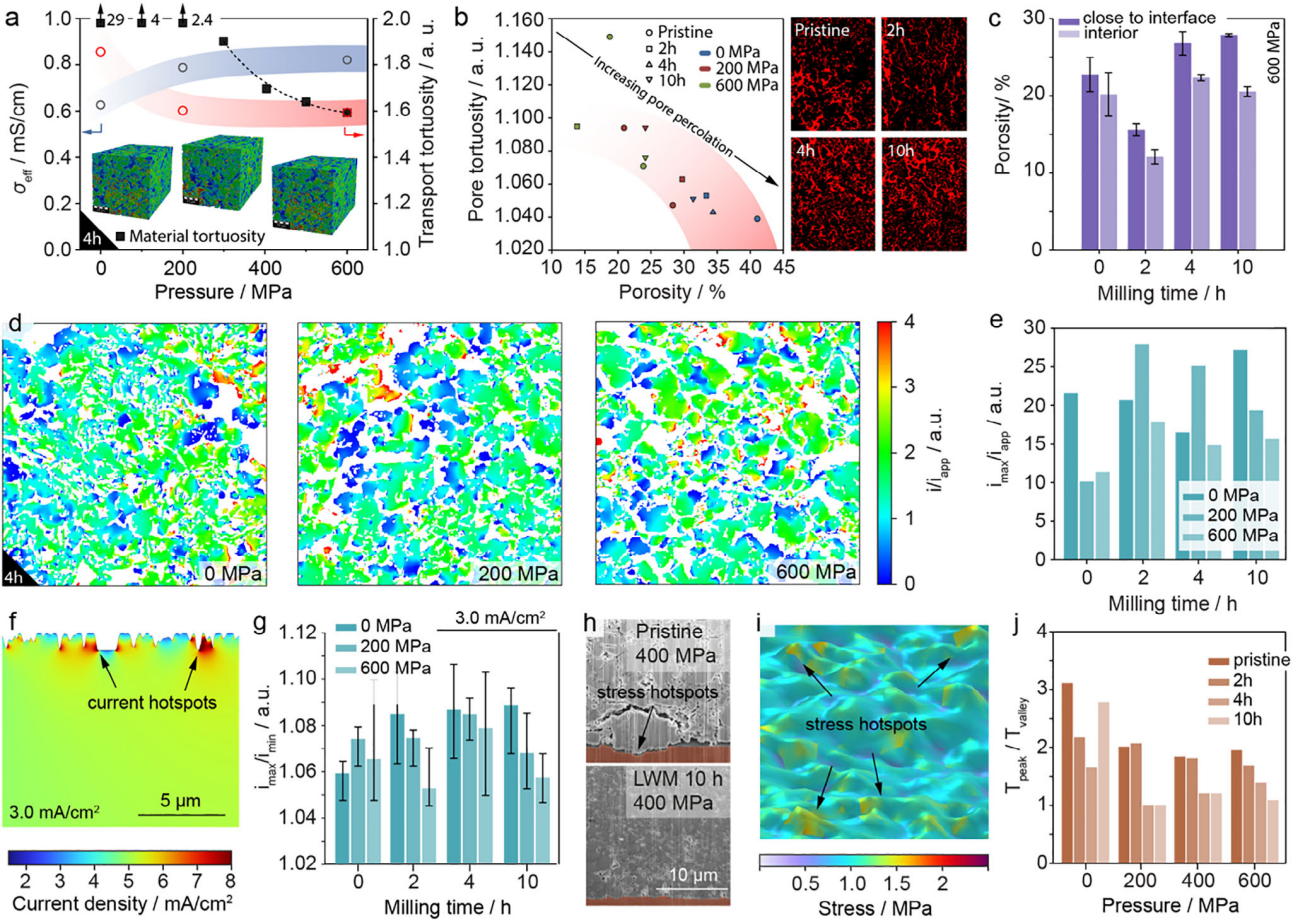
a) Effective Li‐ion conductivities and corresponding tortuosity values as a function of densification pressure for a representative sample set (LWM_4 h), including geometrical tortuosity for comparison. b) Pore percolation trends as a function of porosity for tapes made from different LPSCl powders and densification pressures, with segmented cross‐sections shown for pristine, 2 , 4 , and 10 h milled powders densified at 600 MPa (white = pores, red = percolating pores), indicating persistent percolation. c) Comparison of porosity near the interface and tape interior for all samples densified at 600 MPa. d) Representative current density maps for LWM_4 h densified at 0, 200, and 600 MPa, showing evidence of current focusing at regions with small features. e,f) Distribution of current density within the electrolyte close to the interface, highlighting “hot spots” with maximal currents at rougher regions. g) Correlation between the interface stability descriptor (i_max/i_min) and milling time and densification pressure under an applied current density of 3 mA cm^−2^. h) Cross‐sections of LPSCl SEs, i.e., pristine and LWH_10, pressed at 400 MPa. The SEM images reveal that due to the inhomogeneous shape and size of particles, the application of high pressure causes particles to deform and press against the aluminum foil, leading to its deformation. i) Stress distribution on rough interfaces, and j) variation in the peak‐to‐valley stress ratio with respect to pressure and milling time.

To further investigate the impact of densification on Li‐ion conductivity, we utilized segmented 3D structures and conductivity measurements of compacted powders as inputs to calculate effective Li‐ion conductivities and corresponding transport tortuosity. **Figure** **4**a presents the calculated Li‐ion conductivity and transport tortuosity as functions of densification pressure for tapes fabricated using LWM_4 h (Figure 3f). The calculated conductivity and transport tortuosity of fully densified tapes (p ≥ 600 MPa) align closely with experimental results. However, notable discrepancies are observed for tapes that experienced lower pressure (p < 600 MPa). The convergence of experimental and calculated values at higher densification pressures supports our hypothesis of room‐temperature pressure sintering, as the simulation did not account for interparticle resistances. The initial divergence between experimental and calculated transport tortuosities, followed by their convergence, can be attributed to particle fusion during densification, which reduces interparticle resistances. The increase in calculated Li‐ion conductivity indicates improvements solely related to the enhanced microstructure. Similar trends are evident across all milling durations, as detailed in Table S6 (Supporting Information).

It is also important to note that due to the low T_g_ of HNBR and the fact that geometric tortuosity plays a minor role compared to particle fusion, our findings are applicable to free‐standing LPSCl tapes, which require a higher binder content. This conclusion is further supported by previous studies that observed minimal conductivity loss despite increasing binder content. For instance, Li‐ion conductivity in pressed LPSCl powder (70 MPa) was reported at 1.5 mS cm^−1^. The addition of 1.7 vol% HNBR binder led to a two‐fold decrease in conductivity; however, further increases in binder content had a diminishing effect on conductivity.^[^ ^16^ ^]^

#### 2.2.2 Implication of Porosity and Surface Roughness on Li‐Dendrite Formation

Although the porosity has minor impact on the effective transport properties, it can have a detrimental impact on cell performance. For example, it has been shown that the degree of porosity is linked to the formation of dendrites when Li metal anodes are employed.^[^ ^26^ ^]^ Diallo, et al. have demonstrated that the most prevalent failure mechanism in powder pressed samples is the Li‐filament growth through percolating pores. They also have shown that the Li‐filament growth is suppressed when a critical relative density of ≈95% is achieved and the percolating pore network is closed.^[^ ^26^ ^]^ Herein, all tapes – independent of PSD and densification pressure, have lower density and show throughout a percolating pore network. In Figure 4b exemplary cross sections indicating pore connectivity are shown. The corresponding segmented images illustrate the 3D pore distribution within the tapes as shown in Figure S8 (Supporting Information). Qualitatively it is already evident that most pores are percolating which will lead to low pore tortuosity values. Additionally, in Figure 4b the plot of pore tortuosity against the porosity is shown. Clearly with increasing porosity, pore tortuosity is decreasing but pores remain percolating at any point. Moreover, analysis of bulk and surface microstructure pressed at, e.g., 600 MPa reveal that porosity is even more pronounced near to surface as shown in Figure 4c. These findings suggest that uniaxial densified tapes will be very prone to failure by Li filaments even at relatively low current densities or under modest stack pressure (see Figures S9, S10, Supporting Information). This will be even more pronounced by the mechanical weakness perpendicular to the densification direction. A detailed study on the impact of porosity in tapes on the critical current density (CCD) is out of the scope of this manuscript and will be published elsewhere.

Current focusing as a consequence of surface roughness has been identified as an additional important aspect responsible for the failure of SSBs. For example, Singh et al. studied fine and coarse grained powders in this regard and found that larger grains form rough interface with unfavourable geometries which results in preferential lithium growth at these points.^[^ ^27^ ^]^ In contrast finer grains resulting in a smoother interface, which results in a more uniform current distribution, hence, lead to a more uniform lithium deposition.^[^ ^14c^ ^]^

In order to categorize the interface stability, previous studies used the ratio between the maximum and minimum current density (i_Max_/i_Min_) as a metric for evaluation, in which a high value represents a geometrically heterogeneous interface that is more susceptible to failure.^[^ ^14^, ^28^ ^]^ This lower stability is associated with a non‐uniform potential distribution along the interface which causes fluctuations in the reaction current, leading to localized areas of high current density where the electrolyte is less stable. This criterion is also employed here to compare different tapes generated using LPSCl with different PSD and pressures. Therefore, the surface roughness has been extracted from cross‐sectional SEM pictures (Figure S11, Supporting Information), which was afterward used to create the interface of the model geometry (Figure S12, Supporting Information) and calculate the current density distribution using electrochemical modelling and analysis (EA). The electric potential along with the electrical current streamlines within the electrolyte calculated by the model are depicted in Figure S13 (Supporting Information). Distortion of streamlines close to the interface indicates that the electric current is influenced by the roughness of the electrolyte, leading to localized areas of high current density where the electrolyte is less stable (Figure 4f). Figure S14 (Supporting Information) illustrates the variation in reaction current density (calculated via Bulter‐Volmer equation) for a representative case, highlighting regions with maximum and minimum values (see Figure S15 (Supporting Information) for current profiles of different samples prepared under various milling times and compressional pressures). The calculated current rations (i_Max_/i_Min_) are compared for various microstructures in Figure 4g (see Figure S16, Supporting Information). It is evident that an increasing compressive pressure leads to lower ratios, indicating the potential beneficial impact of higher densification pressure in respect of the cell performance. A systematic correlation between milling time and the current ratio, however, cannot be established.

Despite the surface roughness, voids at the interface can significantly influence current distribution similar to Li metal voids that lead to serious current constrictions along the interface.^[^ ^29^ ^]^ Using 3D effective conductivity calculations (TA) on FIB‐SEM images provides detailed insights on transport processes and current distributions. As shown in a representative set of samples in Figure 4d and the corresponding current ratio plots in Figure 4e, the application of higher‐pressure results in a more homogeneous current distribution across the 2D interface. However, even under a densification pressure of 600 MPa, residual porosity persists, giving rise to localized current hotspots that exceed the applied current density by a factor of more than 15. Based on the TA the area at the interface can be quantified which exceeds a given critical current density (CCD), giving an indication on the risk of Li filament formation. A detailed analysis of CCDs including windows for safe operation of the cells is given in Note S2 (Supporting Information). When assuming a local CCD of 1 mA cm^−2^ we estimate an upper limit of 0.1 mA cm^−2^ applied current density for safe operation.^[^ ^30^ ^]^ At higher current conditions are likely to critically compromise the cell's stability, ultimately leading to failure via short‐circuiting.

#### 2.2.3 Implication of Porosity and Surface Roughness on the Pressure Distribution at the 2D‐Electrode|LPSCl Interface

The microstructure of pristine, non‐densified LPSCl tapes reveals that uneven particle distribution leads to misaligned particles forming a network of voids and smaller particles that are insufficiently compacted. Some particles, however, experience excessive pressure, penetrating even the Al metal foil on which they are coated (Figure 4h). This heterogeneity in stress distribution poses significant challenges for the integration of such materials in SSBs. For example, Si anodes operate best within a narrow pressure range. Hence, localized high or low pressures alter utilization efficiency and affect performance due to the pressure‐dependent redox potential of Si that can even lead to Li plating hence short circuits.^[^ ^31^ ^]^

To elucidate the role of surface roughness in stress distribution, we mapped the surface of the tapes using optical microscopy (Figures S17, S18, Supporting Information). Surface topographies revealed two primary variations: 1) sharp, localized peaks protruding from the surface, and 2) global surface inclinations (Figure S19, Supporting Information). These features generate regions of varying elevations, which we modelled by simulating the application of stress (MA) via a pressing block (Note S3 and Figure S20, Supporting Information). In analogy to the current ratio defined above, the stress ratio between neighbouring peaks and valleys (peak‐to‐valley stress ratio, T_Peak_/T_Valley_) serves as a metric for interfacial quality. In Figure 4i the stress distribution of a representative sample is shown. The calculated peak‐to‐valley stress ratios are illustrated in Figure 4j. As shown, a general decreasing trend is observed with increasing pressing pressure and milling time, suggesting that both parameters enhance uniformity in stress distribution at the SE/electrode interface, thereby suggesting to improving electrochemical performance when applied in a SSB. This is aligned with previous experimental and theoretical observations, suggesting that increasing compressing pressure improves SE/Electrode contact and interface conductivity in addition to inhibiting the formation of voids.^[^ ^32^ ^]^ Based on the observed results (Figure 4j), the pressure has a considerable impact on the reducing the interface heterogeneity up to 200 MPa. The effect diminishes in greater pressures. Gupta et al. has reported similar observations for stack pressure and its effect on interface impedance. Concluding, local variations of a factor of 3 can be expected solely related to the surface roughness, not including potential multiplication by directional stress due to uneven PSD through the SE.

## 3 Conclusion

This study focuses on the optimization of LPSCl separator tapes for SSBs by examining the effects of PSD refinement and densification pressure. Firstly, we explored the impact of different milling protocols, including varying BPRs, milling durations, and the addition of solvents. We identified conditions that allow for tailoring and narrowing PSDs, with wet milling protocols demonstrating superior performance in balancing size reduction and maintaining structural integrity, resulting in conductivities close to the pristine powder. TEM and XRD analysis revealed that dry milling primarily breaks agglomerates without further reducing crystallite size, while wet milling preserves crystallinity and avoids significant amorphization, thus maintaining higher Li‐ion conductivity.

Secondly, we examined the role of PSD in the densification processes of slurry‐casted tapes as a function of the applied uniaxial densification pressure (up to 1000 MPa). Tapes were fabricated using LWM‐processed LPSCl powders with HBNR binder and p‐xylene as solvent. Operando EIS analysis during the densification process revealed a threshold pressure at ≈350 MPa at which Li‐ion conductivity significantly increases. FIB‐SEM tomography in combination with FEM showed that the increase in conductivity is not solely due to increased density and reduced tortuosity. Only minor changes in tortuosity were observed, with a significant portion of open porosities (≈30%) oriented along the densification direction. The increase in Li‐ion conductivity was attributed to particle fusion (“cold sintering”) when further densification by particle rearrangement or fracturing is no longer possible.

Lastly, we investigated the implications of porosity and surface roughness when LPSCl tapes are employed in a SSB. Found persistent surface irregularities and residual percolating porosity parallel to the densification direction will pose significant performance challenges in SSBs. For example, surface roughness and interfacial voids at the LPSCl/Li metal interface can locally amplify current densities by a factor of >20, compromising the ability of LPSCl tapes to allow only low cycling rates. Additionally, the presence of open porosity facilitates Li filament growth along percolating pathways, bypassing the need to penetrate solid material and amplifying the risk of dendritic failure.

Our findings emphasize the necessity for further refinement of both powder processing and densification techniques to significantly enhance the potential for high‐performance, scalable SSB separators. These advancements are crucial for the practical adoption of SSBs in EVs and other high‐energy applications.

## 4 Experimental Section

### Samples—Ball Milling

Commercial LPSCl powder was procured from NEI Corporation and processed using both HDM and LWM. Both methods utilized 1 mm zirconia milling balls (MSE) in a Pulverisette 7 classic line (Fritsch). The BPR used were 10:1, 20:1, and 30:1. For instance, a BPR of 20:1 involved combining 30 g of zirconia milling balls with 1.5 g of as‐received LPSCl powder in a 12 mL zirconia grinding bowl. The grinding bowl was tightly sealed under argon atmosphere inside a glovebox. Milling durations of 2, 4, and 10 h were employed, consisting of cycles of 30 min of milling followed by 30 min of rest.

HDM was initially carried out without solvent at a rotational speed of 600 rpm and a fixed BPR of 30:1 for 2, 4, and 10 h. To further refine the PSD, additional milling experiments were conducted on the as‐received LPSCl powder using BPRs of 10:1, 20:1, and 30:1 for 10 h. After milling, the dry LPSCl powder was collected and sieved to separate it from the milling balls. Any residual powder adhered to the jar walls was carefully removed using a brush. The resulting powder was stored inside an argon‐filled glove box for subsequent experiments.

For LWM, 7 mL of p‐xylene was added to each jar to facilitate the milling process and minimize particle agglomeration. Thorough hand mixing was performed to ensure the solvent was evenly distributed. Milling was performed at a rotational speed of 200 rpm using a BPR of 20:1 for durations of 2, 4, and 10 h. To study the effects of BPR on LWM, additional experiments were conducted using BPRs of 10:1, 20:1, and 30:1 for 2 h each, with the as‐received LPSCl powder. After milling, the resulting slurry containing LPSCl, p‐xylene, and milling balls was dried in an oven at 80 °C for 48 h to remove the solvent. The dried material was hand‐sieved to separate the LPSCl powder from the zirconia milling balls. To ensure complete solvent removal, the collected powder was further dried at 80 °C under vacuum for an additional 48 h and stored inside an argon‐filled glove box.

### Samples—Tape Casting

The LPSCl slurry was prepared by mixing 97.5% LPSCl with 2.5% HNBR binder in p‐xylene solvent. The materials were stirred using a magnetic stirrer at 500 rpm for 8 to 9 h to ensure homogeneity. The resulting slurry was then coated onto aluminum foil using the doctor blade method. The coated slurry was initially air‐dried and subsequently heated at 80 °C overnight. After complete drying, the LPSCl tapes were punched into 10 mm diameter discs using an electrode cutter. All procedures were conducted inside an argon‐filled glove box, and the dried SE tapes were stored in the glove box for further experimentation.

### Physical Characterizations—FIB‐SEM Tomography

To prevent atmospheric degradation, samples were transferred from a glovebox to the SEM using a Zeiss sample transfer shuttle. FIB‐SEM tomography was carried out using a Zeiss Crossbeam 550. A protective platinum (Pt) layer was initially deposited on the sample surface using a 30 kV, 7 nA beam, followed by the milling of tracking markers into the Pt layer (30 kV, 100 pA). Subsequently, a thick C layer was deposited on top of the Pt layer (30 kV, 7 nA) to enhance marker visibility, ensure accurate tracking, and further mitigate ion beam damage and surface charging. To minimize charging effects, the protective layer was connected to the conductive silver glue layer by depositing a Pt bridge (30 kV, 700 pA). Field emission scanning electron microscopy (FESEM) imaging was conducted using an acceleration voltage of 2 kV and a current of 1 nA. The FIB‐SEM tomography was carried out over a volume of 20 × 20 × 20 µm^3^, using a pixel size of 10 nm and a slice separation of 25 nm. The ion milling was performed using a 30 kV and 1.5 nA beam to ensure efficient material removal while preserving structural integrity of the sample. FESEM images were captured sequentially after each milling step using a pixel dwell time of 0.6 µs to optimize image contrast and reduce charging effects. For additional information refer to Note S1 (Supporting Information). Post‐tomography, EDS was performed using an Oxford Ultim Extreme detector. The EDS mappings were acquired over an effective area of 592 × 592 pixels, with an acceleration voltage of 3 kV and a beam current of 3 nA to obtain compositional data.

### Physical Characterizations—Powder XRD and Quantification of Amorphization

XRD patterns of LPSCl powder were recorded using a domed sample holder to protect the sample from air and moisture. The sample was loaded under argon‐filled glovebox and transferred to a Bruker D8 A25 DaVinci X‐ray Diffractometer with Cu Kα_1_ (λ = 1.5406 Å) and Cu Kα_2_ (λ = 1.5444 Å) radiation at room temperature. Data were collected over a 2θ range of 10–90° with a step size of 0.024° and a step time of 2 s, resulting in a total scan duration of 100 min. To assess the degree of amorphization after HDM and LWM, LPSCl powders were blended with 50 wt% high‐purity crystalline silicon powder, used as an internal standard due to its sharp, well‐resolved diffraction peaks and minimal overlap with LPSCl signals. XRD measurements were performed over a 2θ range of 10–80°, with a step size of 0.026° and a step time of 1.240 s. The amorphous content was quantified using the internal standard method implemented in Topas.

### Physical Characterizations—TEM

TEM was performed with a cold FEG, JEOL ARM 200F, operated at 200 kV. A Gatan model 648 vacuum transfer double tilt holder was used to load the samples inside a glove box and transfer them inertly into the high vacuum column of the TEM. Selected area electron diffraction and bright field TEM imaging were done with a low dose to avoid electron beam damage.

### Physical Characterizations—SEM

SEM was performed with an FEI Apreo. A working distance of 2 mm was maintained for all samples. The imaging was carried out in SE mode with an acceleration voltage of 5 kV, a probe current of 0.1 nA, and the Everhart–Thornley detector.

### Physical Characterizations—Machine‐Learning Image Segmentation, Pore Connectivity, and TA

To segment pore and material phases within the tomography volumes, a convolutional neural network based approach was utilized. Specifically, an attention residual U‐Net model implemented in the Python Keras library was trained on datasets composed of 512 × 512 pixel images and their corresponding labels, using 100 training epochs on an NVIDIA RTX A5000 GPU. Initial labels were generated through gray‐value thresholding in Python and manually refined using open‐source software.^[^ ^33^ ^]^ This machine‐learning‐based method offered significant advantages over conventional thresholding, particularly in distinguishing phases with similar gray values. For additional details, see Note S4 (Supporting Information). The resulting 3D segmented volumes were used for further quantitative analyses. Following segmentation, pore connectivity was assessed using a 3D connected‐component labelling algorithm based on 26‐connectivity, wherein voxels sharing faces, edges, or corners were considered connected. Each distinct pore cluster was assigned a unique label. Pore structures that appeared in both the top and bottom slices of the volume were identified as fully connected pores, representing continuous transport pathways through the sample thickness. From this, a connected pore mask was generated to quantify the fraction of total pore volume that is interconnected—an essential parameter for evaluating permeability and transport behavior. To complement the connectivity analysis, tortuosity was computed by evaluating the geodesic distance through the connected pore network. A binary mask of the pore phase was first generated, and the shortest path through the pores was calculated using the fast‐marching method. The geodesic distance was then normalized by the corresponding Euclidean distance to determine a tortuosity value for each voxel. For each slice, only tortuosity values ≥ 1 were considered in computing the average tortuosity. This analysis was performed along the top‐to‐bottom (z) direction, which aligns with the primary transport axis of the material. Final 3D rendering and visualization were carried out using Avizo 3D (version 2023.2).

### Physical Characterizations—PSD Analysis

The obtained SEM images were processed using ImageJ, a Java‐based, open‐source image processing and analysis software developed by the National Institutes of Health (NIH), USA.

### Physical Characterizations—Optical Microscopy

Surface roughness was measured through Alicona – Infinite focus. The samples were vacuum sealed in a sample holder inside argon filled glovebox. For acquisition of images, IF‐ ObjectiveRL 10x magnification lens was used. The software used for image viewing and analysis was Alicona‐ Laboratory Measurement Module 6.6.9.

### Electrochemical Characterization—EIS

For measuring Li‐ion conductivity of powder, 10 mm PEEK cell was employed, and 100 mg of powder was used for each measurement. The powders were first compacted into pellets by applying a uniaxial fabrication pressure of 375 MPa for 3 min using a hydraulic press. Afterward, the applied pressure was completely released. For impedance measurements, a constant stack pressure of 100 MPa was applied to ensure consistent contact during the measurement process. The EIS measurements were conducted using a BioLogic potentiostat, with the frequency range extending from 7·10^6^  to 10⁻^2^ Hz. The data obtained were used to determine the ionic conductivity of the pellets. The Li‐ion conductivity of LPSCl tapes was measured using a CompreDrive automated die press (RHD Instruments) equipped with a 12 mm die configuration. Impedance measurements were conducted under stack pressures of 0, 100, 200, 300, 400, 500, 600, 700, 800, 900, and 1000 MPa. A Biologic VMP300 was employed for the impedance analysis.

### Numerical Modeling and Analysis—EA

Electrochemical modeling was performed to investigate the impact of milling time and compressing pressure on the reaction current density on the SE/electrode interface. The model was implemented using FEM integrated within COMSOL Multiphysics (Version 6.1). To develop the model, the following governing differential equation (GDE) was employed:

(1) $$\nabla \cdot \left(k_{\mathrm{SE}}\nabla \phi_{e}\right)=0$$

where k_SE_ was the ionic conductivity and ϕ_e_ represented the electric potential of the SE. The electrochemical reaction at the electrode/SE interface was modelled using the Butler‐Volmer equation:

(2) $$i_{\mathrm{BV}}=i_{0}\;\left[\exp\left(\frac{\alpha_{a}F}{\mathrm{RT}}\eta \right)-\exp\left(-\frac{\alpha_{c}F}{\mathrm{RT}}\eta \right)\right]$$

In this equation, i_0_ was the exchange current density, T was the absolute temperature α_a_, α_a_ were the anodic and cathodic charge transfer coefficients (both set to 0.5), F was Faraday's constant, R was the universal gas constant, η was the activation overpotential. The overpotential is defined as η  = ϕ_s_  − ϕ_e_ − *U_Li_*, where ϕ_s_ is solid phase potential and *U_Li_* is open circuit potential. ϕ_s_ is set to zero owing to sufficiently high Li metal conductivity. Since the open‐circuit potential of Li versus Li is zero, the overpotential simplifies to η  =   − ϕ_e_.

Assuming a system similar to the one depicted in Figure S12 (Supporting Information), where the SE layer (blue) lied on top of electrode (red) with a conformal contact, the boundary conditions will be described as follows:

(3) $$-k_{\mathrm{SE}}\nabla \phi_{e}\cdot n\;=i_{\mathrm{app}}\;\left(\mathrm{at}\;\mathrm{the}\;\mathrm{top}\;\mathrm{face}\;\mathrm{of}\;\mathrm{SE}\right)$$

(4) $$-k_{\mathrm{SE}}\nabla \phi_{e}\cdot n\;=i_{\mathrm{BV}}\;\left(\mathrm{at}\;\mathrm{the}\;\mathrm{SE}/\mathrm{Electrode}\;\mathrm{interface}\right)$$

(5) $$\nabla \phi_{e}\cdot n\;=0\;\left(\mathrm{at}\;\mathrm{the}\;\mathrm{left}\;\mathrm{and}\;\mathrm{right}\;\mathrm{boundaries}\right)$$

Here, i_app_ represented the applied current density entering the domain and n is the unit vector normal to the surface, respectively. The required parameters for solving the model are summarized in Table S7 (Supporting Information).

The electrolyte was modelled as a square domain with dimensions of 25 × 25 µm, where the lower rough surface represents the SE/electrode interface. As mentioned earlier, the interface morphologies were constructed using SEM images, which captured the surface characteristics as functions of milling time and pressure. This approach linked milling time and pressure to surface roughness, and subsequently, to the reaction heterogeneity at the interface.

To generate the morphology, a sampling line was assumed on the SEM image and its intersection with void and matrix regions determined the surface roughness at the interface (Figure S11, Supporting Information). Since the resulted morphology can vary depending on the sampling line, the process was repeated at least five times across different parts of the image. The results were then averaged, and the variations were reported to ensure a reliable characterization of the interface for each case.

Triangular elements were employed to discretize the electrolyte domain, with mesh refinement concentrated near the interface. This ensured high‐precision modeling in this critical region and enabled accurate capture of the reaction current's variations while maintaining low computational costs. The mesh was refined iteratively until mesh independence was achieved, which occurred at ≈100000 elements.

### Numerical Modeling and Analysis—Effective Conductivity and TA

In order to analyze the segmented 3D FIB tomographies with respect to effective conductivity, tortuosity, and current distribution, virtual conductivity measurements were conducted with the ConductoDict module of the software GeoDict. The simulation geometries were rescaled to 25 nm voxel size. Assuming a homogeneous, isotropic solid phase obeying Ohm's law, and applying a fixed potential difference Δϕ_*e*,ext_ between opposite boundaries, the virtual measurement can be modelled by the following set of PDEs: the Poisson equation, Equation (1), is applied to the domain Ω (i.e., the solid phase). This is coupled with Dirichlet boundary conditions on the edge of the plates Γ_1_ and Γ_2_ (i.e., a fixed potential difference) and symmetric boundary conditions on the perpendicular four surfaces, i.e.

(6) $$\phi_{e}\;\Gamma_{1}=\Delta \phi_{e,\mathrm{ext}}$$

(7) $$\phi_{e}\;\Gamma_{2}=0$$

(8) $$\left(\nabla \phi_{e}\cdot n\right)\;\partial \Omega \setminus \left(\Gamma_{1}\cup \Gamma_{2}\right)=0$$

This set of PDEs was solved using GeoDict's LIR solver to obtain a spatially resolved current density distribution for each tomography. The total current *i*_tot_ can then be used to calculate the effective conductivity *k*_SE,eff_ by applying Ohm's law

(9) $$i_{\mathrm{tot}}=-k_{\mathrm{SE},\mathrm{eff}}\;\Delta \phi_{e,\mathrm{ext}}$$

The tortuosity τ_SE_ of each structure can then be evaluated according to

(10) $$k_{\mathrm{SE},\mathrm{eff}}=\frac{\varepsilon_{\mathrm{SE}}}{\tau_{\mathrm{SE}}}\;k_{\mathrm{SE}}$$

where ε_SE_ denotes the solid volume fraction. Further information on the TA simulations is detailed in Note S5 (Supporting Information).

### Numerical Modeling and Analysis—MA

In addition to electrochemical modeling, FEM was employed to investigate the correlation between surface roughness and stress distribution across various samples. Further details are provided in Note S3 (Supporting Information).

## Conflict of Interest

The authors declare no conflict of interest.

## Supporting information



Supporting Information

## Data Availability

The data that support the findings of this study are available from the corresponding author upon reasonable request.
