# Supplementary material for: Uni‐Axial Densification of Slurry‐Casted Li₆PS₅Cl Tapes: The Role of Particle Size Distribution and Densification Pressure
Source: Adv Mater. 2025 May 19;37(30):2501592. doi: 10.1002/adma.202501592 (PMC12306411; doi:10.1002/adma.202501592)
Supplement: Supplementary file 1 — Supporting Information [file ADMA-37-2501592-s001.docx]

**Supplementary Information**

**Uni-Axial Densification of Slurry-Casted Li₆PS₅Cl Tapes: The Role of Particle Size Distribution and Densification Pressure**

Quoc-Anh Tran^1,2,$^, Meenal Agrawal^1,2,$^, Michael Häusler^3^, Johannes Hörmann^4,5^, Mohsen Sadeqi Moqadam^1^, Günther J. Redhammer*^6^*, Ingeborg Sellæg Ellingsen^1^, Mir Mehraj Ud Din^1,2^, Per Erik Vullum^7,8^, Roman Zettl^9^, Timo Danner^4,5^, Arnulf Latz^4,5,10^, Volker Henninge^9^, Roland Brunner^3^, Daniel Rettenwander^1,2,11,^*

*^1^Department of Materials Science and Engineering, NTNU Norwegian University of Science and Technology, Trondheim, Norway*

*^2^Christian Doppler Laboratory for Solid-State Batteries, NTNU Norwegian University of Science and Technology, Trondheim, Norway*

*^3^Materials Center Leoben Forschung GmbH, Leoben, Austria*

*^4^German Aerospace Center (DLR), Institute of Engineering Thermodynamics, Stuttgart, Germany*

*^5^Helmholtz Institute Ulm for Electrochemical Energy Storage (HIU), Ulm, Germany*

*^6^Department of Chemistry and Physics of Materials, University of Salzburg, Salzburg 5020, Austria*

*^7^Department of Physics, NTNU Norwegian University of Science and Technology, Trondheim, Norway*

*^8^Sintef Industry, Trondheim 7034, Norway*

*^9^AVL List GmbH, Graz, Austria*

*^10^Institute of Electrochemistry, Ulm University, Ulm, German*

*Corresponding authors: [d](mailto:d)aniel.rettenwander@ntnu.no

^$^These authors contributed equally

**Note S1. Tomography Preparation**

Figure S5a shows the deposited protective platinum (Pt) layer on the sample surface, which was applied using a 30 kV, 7 nA ion beam. Figure S5b illustrates the FIB-cut cross-section at the end of the tomographic scan with the Pt layer on top of the underlying sample. The cross-section corresponds to the slice plane (x, y- direction) during the tomography.

Following the Pt deposition, tracking markers were milled into the Pt layer (30 kV, 100 pA) to allow precise sample alignment (shown in the red figure inset). A subsequent carbon (C) layer was added to further enhance marker visibility, improve tracking accuracy, and provide additional protection against ion beam damage during the tomography process.

**Note S2. Analysis of maximum currents and critical current densities**

The currents at a 2D-electrode|LPSCl interface in relation to milling time and compacting pressures were investigated. For this, the 3D current distributions obtained by the procedure detailed in Subsection “Effective Conductivity and Transport Analysis” of Section 4.d) of the main manuscript were processed and analysed. Details on the preprocessing of the FIB tomographies as well as simulation parameters are provided in Note S5. In order to analyse the degradation risk at the 2D-electrode|LPSCl interface, the 3D microstructure-resolved current distributions obtained from the virtual conductivity measurements were examined at the surfaces of the structures. The “top” side or interface always refers to the side of the FIB tomographies which corresponds of the top side of the tapes. The “bottom” interface always refers to the opposite side. Heatmap plots the obtained current density distributions at each interface are depicted in Figures S23-S26. A critical current density (CCD) of 1 mA/cm² was chosen based on previous work by Neumann et al.^[1]^. The top as well as the bottom interface were both analysed. Nevertheless, compacting pressures in the compressed samples caused an increasing solid volume fraction (SVF) of the structures from top to bottom. This resulted in generally more homogeneous current distributions at the bottom side.

**a) Nondimensionalization of currents in the analysis**

Throughout the analysis of critical currents, all currents are considered as relative values $i/i_{app}$. Therefore, any absolute current or current density value can be obtained by simply multiplying with the applied current (density). This nondimensionalization is possible because any mathematical solution to the PDE system consisting of eqs. (1) and (6)-(8) for a given potential difference can be transformed to a solution for another arbitrary potential difference by a simple rescaling. The numerical approximations of solutions to the PDE system inherit this property up to numerical accuracy of the applied solver. This was also tested by comparing a variety of numerical solutions for different applied potential differences. Vividly speaking, a conductor obeying Ohm's law has the same property, i.e. current scaling linearly with the applied potential difference. By assuming Ohm's law to hold in every point of the microstructure, this property translates to the complex global solution of the microstructure-resolved simulation. It should be emphasized, that this is only possible because we are not solving the full electrochemistry of the system. For example, effects due to grain boundaries in the SE or electrochemical reactions at the interfaces in contact with electrodes are neglected. Apart from simplifying the relations between a CCD and an applied current, this enables simulations on a much larger domain. In our case this enabled simulations on the FIB-SEM tomographies with only little downscaling and no reducing of the cut-out size allowing focus on the statistical features of the microstructure.

**b) Investigation of maximum currents**

For the investigation of the maximum currents at the interfaces, two different metrics were used. On the one hand, the absolute maximum was calculated at each interface. Note, that these maxima can be influenced by very small SE clusters, including artifacts of the reconstruction process. Therefore, a second property was determined which captures more statistical information for comparison on the other hand. The current $j_{>3\sigma}$ is defined for this purpose. It describes the (normalized) current, such that approximately 99.865% of the area has a normalized current below this value. This value for the area corresponds to 3 times the standard deviation of a normally distributed quantity $X$, i.e. $\Pr\left( X\leq\mu+3\sigma\right)\approx$ 99.865%. In empirical sciences, this is considered as near certainty. $j_{>3\sigma}$ can be therefore considered as a statically significant maximum value for comparison. The extracted values for the top and bottom interfaces are shown in Tables S11 and S12, respectively.

When looking at the maximum values $\max\left( j \right)$, it can be seen that an increase of applied compacting pressure does not necessarily decrease the maximum. This indicates that independently of the applied pressure, local currents could exceed the applied one by more than one order of magnitude. In comparison, $j_{>3\sigma}$ correlates reliably with the applied pressure at the bottom layer and is significantly reduced by higher pressures. This improvement of transport is not only due to an increase in SVF but an improved conductive network. By looking at the related average current in the layer, which directly reflects the expected decrease in current due to an increased SVF this can be verified. Nevertheless, even the most favorable cases show quite high currents. That is, for the bottom layers of the 600 MPa pressure samples, $j_{>3\sigma}$ still exceeds the applied current by a factor of 3.4 to 4.5. In order to get a better understanding of the possible implications of the maximal currents on degradation, the analysis was complemented by an investigation of currents in relation to a critical current density.

**c) Investigation of critical current density (CCD)**

Using the information on the determined distributions of normalized currents at each 2D-electrode|LPSCl interface, degradation risk can be assed based on a CCD. By assuming a value for the CCD and an applied current, the area at the interface can be quantified which exceeds the CCD and consequently compromises the cell's stability. Conversely, this can give guidelines on how one would need to restrict applied currents for safe operation with minimized risk of degradation. We determined this “compromised area” for applied currents of 0.1 mA/cm², 0.5 mA/cm², and 3 mA/cm². The extracted areas are given in Tables S11 and S12 together with visualizations in Figures S25-S28. When applying a current of 3 mA/cm², a majority of the area lies beyond the CCD (more than 85%), which is expected for an applied current exceeding the CCD. For 0.5 mA/cm², clear trends can be observed. By increasing pressure and to a smaller degree by increasing milling time, the compromised area can be reduced significantly. These positive effects on the area are only consistently achieved on the bottom interface, which is in agreement with our analysis of maximum currents. From around 30% in the worst-case, the compromised area can be reduced up to 2 to 3 %. On the one hand, this shows the tunability of compromised area by the LPSCl processing parameters. On the other hand, it is also evident that a non-negligible area still persists above the CCD critically compromising the cell's stability. Finally, by going as low as 0.1 mA/cm², the area approaches 0 for increasing milling times and pressure. Especially the bottom side cases show promising scenarios with an area even equal to exactly zero.

**Note S3. Numerical modeling of stress distribution at the interface**

To investigate the effect of surface morphology on stress distribution, it is essential to first map the roughness of various samples. This mapping is accomplished through microscopic imaging using an Alicona microscope at a magnification of 50X. The resulting images quantify the electrolytes' roughness at a scale suitable for subsequent mechanical analysis.

Figure S19 presents a microscopic image of one such sample. Notably, the variation along the z-axis is exaggerated in the figure to enhance visualization. Two distinct types of surface variations are evident: (1) **local variations**, represented by sharp peaks protruding from the structure, and (2) **global variations**, indicated by color gradients corresponding to surface inclination (tilting). In this image, the right side of the sample exhibits a higher elevation compared to the left.

This study focuses on understanding the effects of stress variations caused specifically by the surface roughness.

**a) Model Geometry:**

The model geometry consists of two parts: the electrolyte block and the pressing block, which are initially separated. The electrolyte block measures 1000×1000×100 μm and features flat surfaces, except for its top face, where roughness data obtained from microscopic measurements is applied. The pressing block, with identical dimensions and flat surfaces, is designed to apply stress to the electrolyte from the top (Figure S20).

The system is discretized using triangular elements, with mesh refinement concentrated in the region near the roughened surface to ensure accuracy in this area. As with the previous case, mesh refinement is performed iteratively until mesh independence is achieved.

The stress-strain constitutive equations for elastic materials are used to calculate the stress distribution at the interface^[2]^. The boundary conditions are given in Table S8. The mechanical properties of the electrolyte and pressing block are also summarized in Table S9.

**b) Stress at Peaks and Valleys of the surface**

As discussed, surface roughness and overall surface inclination create areas of varying elevations. An electrolyte with a simplified roughed surface is shown in Figure S21. The pressing block applies stress to the electrolyte from the top, resulting higher stress on the peaks related to the valleys. Comparing the stress of the neighbouring peaks and valleys can be used as a criterion to find the optimum microstructure with lowest stress variation. Here, *peak-to-valley stress ratio* ($T_{ratio}={T_{Peak}}/{T_{Valley}}$) is used to quantity the local stress variation. Since peaks and valleys are in neighbouring areas, the ratio is unaffected by the overall surface inclination.

**c) Stress distributions and Peak/Valley ratios for various samples**

Figure S22 presents microscopic images of samples processed under different conditions, alongside their corresponding stress distributions calculated via finite element method (FEM). The stress distribution figures clearly distinguish regions of high and low stress. Therefore, the peak-to-valley stress ratio can be calculated for neighbouring points. To account for variability, multiple sampling points from different areas are analysed, and the average values and standard deviations are reported.

In some cases, such as the pristine sample processed at 0 MPa, high surface inclination results in certain lower-elevation areas not experiencing the stress. For these samples, the peak-to-valley ratio is calculated in regions where stress is predominantly applied.

**Note S4. ML-based segmentation of FIB SEM tomography image**

An attention-residual U-Net model from the Python Keras library was used for the image segmentation task. It is based on the U-Net structure^[3]^, incorporating residual blocks to ease gradient flow and improve feature reuse. Attention gates are introduced at the skip connections to suppress irrelevant features and enhance salient regions in the feature maps, improving segmentation accuracy. Batch normalization and dropout are employed to stabilize training and prevent overfitting. The model was trained using 12 images and their corresponding manually created labels (e.g. Figure S6). For each sample, a representative slice was selected and a region of 1024x1024 pixel was extracted. These images were further divided into four images of 512x512 pixel for model input.

To expand the training dataset, the Albumentations library was employed to apply data augmentation. Each image was augmented four times using various random transformations, with a probability parameter (p) controlling the likelihood of each augmentation being applied to the input data, thereby increasing the diversity of the training set:

- HorizontalFlip(p=0.5)
- VerticalFlip(p=0.5)
- RandomRotate90(p=0.5)
- ShiftScaleRotate(scale_limit=0.1, rotate_limit=30, shift_limit=0.1, p=0.5)
- RandomBrightnessContrast(brightness_limit=0.05, contrast_limit=0.05, p=0.2)

The used model parameters were the following:

- number of classes: 2
- number of epochs: 100
- optimiser: adam
- loss function: binary_crossentropy
- batch size: 1
- activation function: relu
- decoder block type: upsampling, attention gating, residual connections
- validation percent: 30%
- input shape: (512, 512)

The accuracy achieved during training was 99.74% for training accuracy and 97.18% for validation accuracy. Figure S7 shows a slice image unknown to the model, its manually created ground truth label and the label predicted by the model.

**Note S5. Details on TA simulations**

Before conducting virtual conductivity measurements on the 3D FIB tomography data with the ConductoDict module of the commercial software GeoDict, a preprocessing step was performed on the structures.

**a) Preprocessing of structural input**

Structural downscaling was performed, since GeoDict’s LIR solver requires equal voxel length in each spatial direction and the segmented FIB tomography data had a pixel size of 10 nm with a slice separation of 25 nm. The input size of each tomography was a stack of 400 images with 1800x1000 pixels. Rescaling was executed for each tomography by iteratively downscaling each 2D image via local averaging followed by rounding as well as a cluster check. Therefore, the resulting simulation domains had the dimensions 720x400x400 with 25 nm voxel size. In order to assure good quality of the downscaling, the total fraction of changed voxels was tracked and was always between 2 and 4 %. In the cluster check of the geometries, isolated SE clusters were identified and removed as they can cause numerical stability issues and do not contribute to transport through the structure. Connectivity was checked by considering common faces between neighboring solid voxels. Note that any isolated clusters of the void space were intentionally not removed. The total solid volume fraction of removed clusters was below 0.06 % for all geometries.

**b) Simulation and solver parameters**

The PDE system consisting of eqs. (1) & (6)-(8) was solved with the ConductoDict module of the commercial software GeoDict (GeoDict 2023, Revision 61624). The LIR solver was used in the speed-optimized mode with default settings and an error bound of 0.001. By setting the boundary conditions to “Symmetric (Dirichlet)” in “Potential Flux Direction” and “Symmetric” in “Tangential Directions”, one obtains the above-mentioned PDE system. The potential difference was set to 1 V and the intrinsic ionic conductivity $k_{\mathrm{SE}}$ was set to the values measured by impedance spectroscopy depending on the sample. That is 0.003076 S/cm for the tomographies related to pristine powders and 0.002140 S/cm, 0.001741 S/cm and 0.001270 S/cm for 2 h, 4 h, and 10 h of LWM, respectively. The resulting effective conductivities and tortuosities are listed in Table S10 and an analysis of the current distributions obtained by these simulations is detailed in Note S4.

**Supplementary Figures**


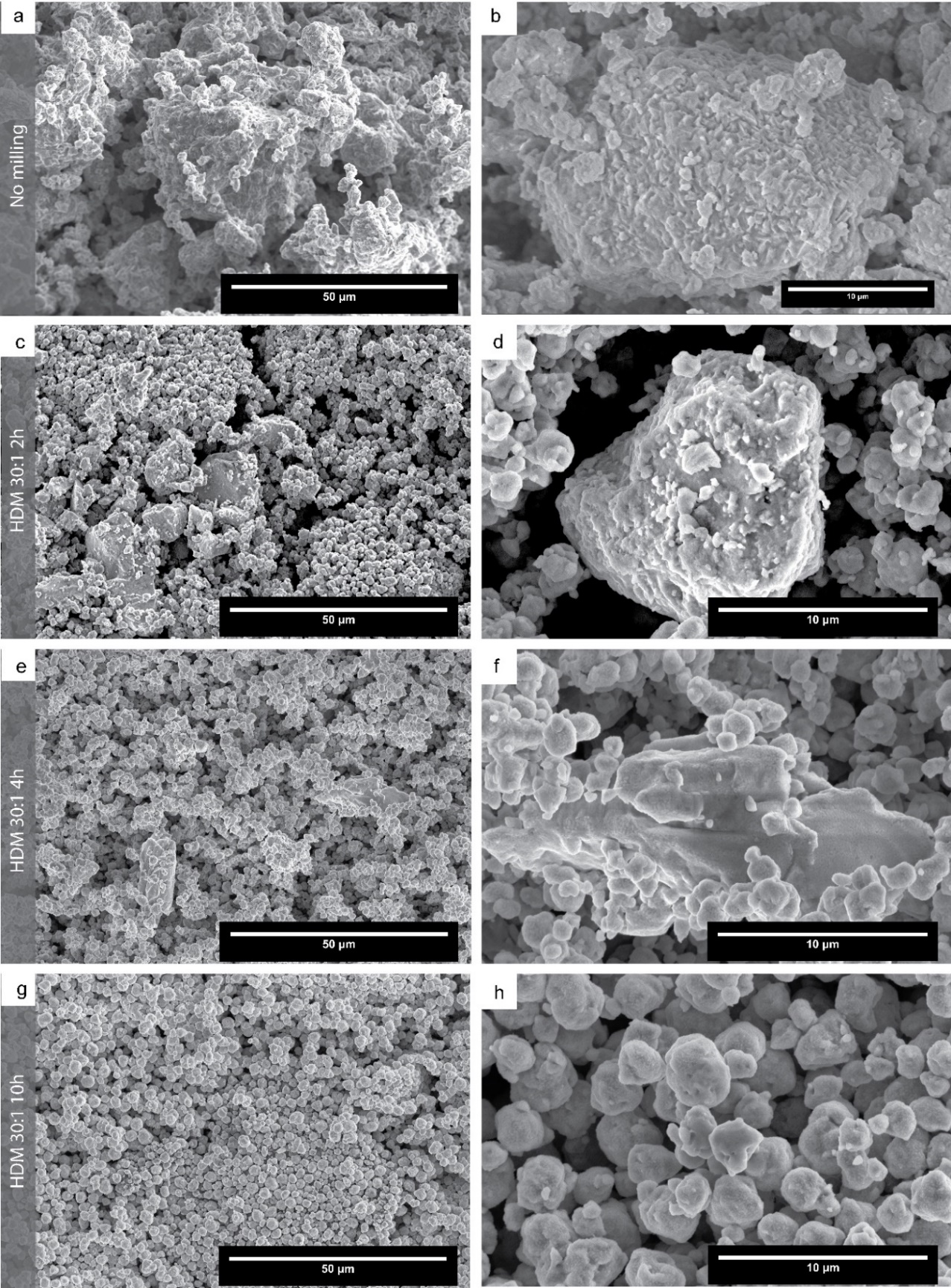


**Figure S1**. SEM images LPSCl powder after high-energy dry milling (HDM) at 600 rpm with a ball-to-powder ratio of 30:1 for milling durations of 2 hours, 4 hours, and 10 hours, illustrating the influence of the milling time used during HDM on the particle size distribution of the particles.


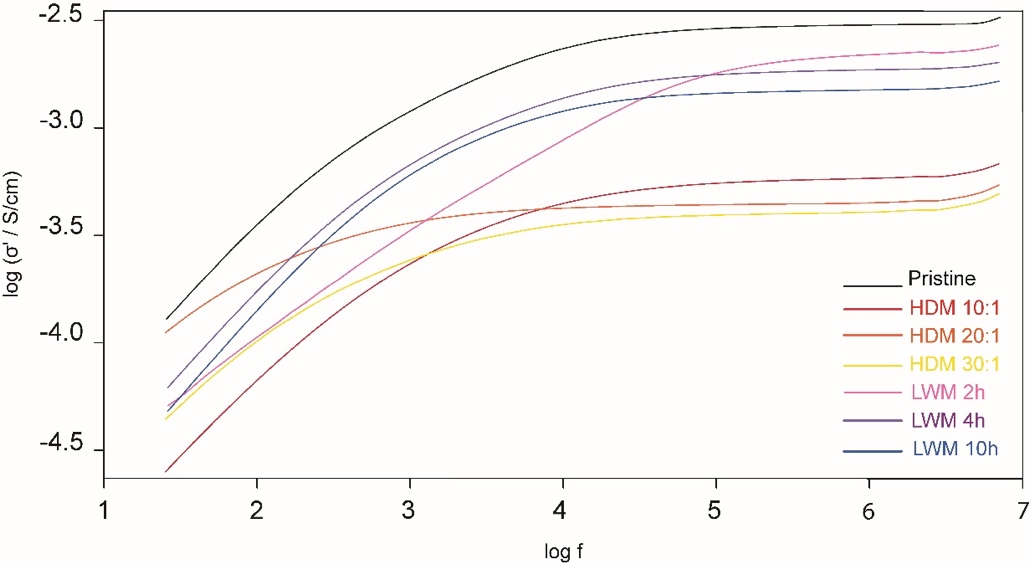


**Figure S2.** Bode plot (log σ vs. log f) derived from EIS measurements for pristine LPSCl powder, high-energy dry milling (HDM) at 600 rpm for 10 hours with ball-to-powder ratios (BPR) of 10:1, 20:1, and 30:1, and low-energy wet milling (LWM) at 200 rpm with a BPR of 20:1 for milling durations of 2, 4, and 10 hours. Electrical processes, which appear as semicircles in Nyquist plots (Z′′ vs. Z′), manifest as plateaus in the corresponding conductivity isotherms. The electrolyte conductivity is extracted from the turning point of the plateau region.


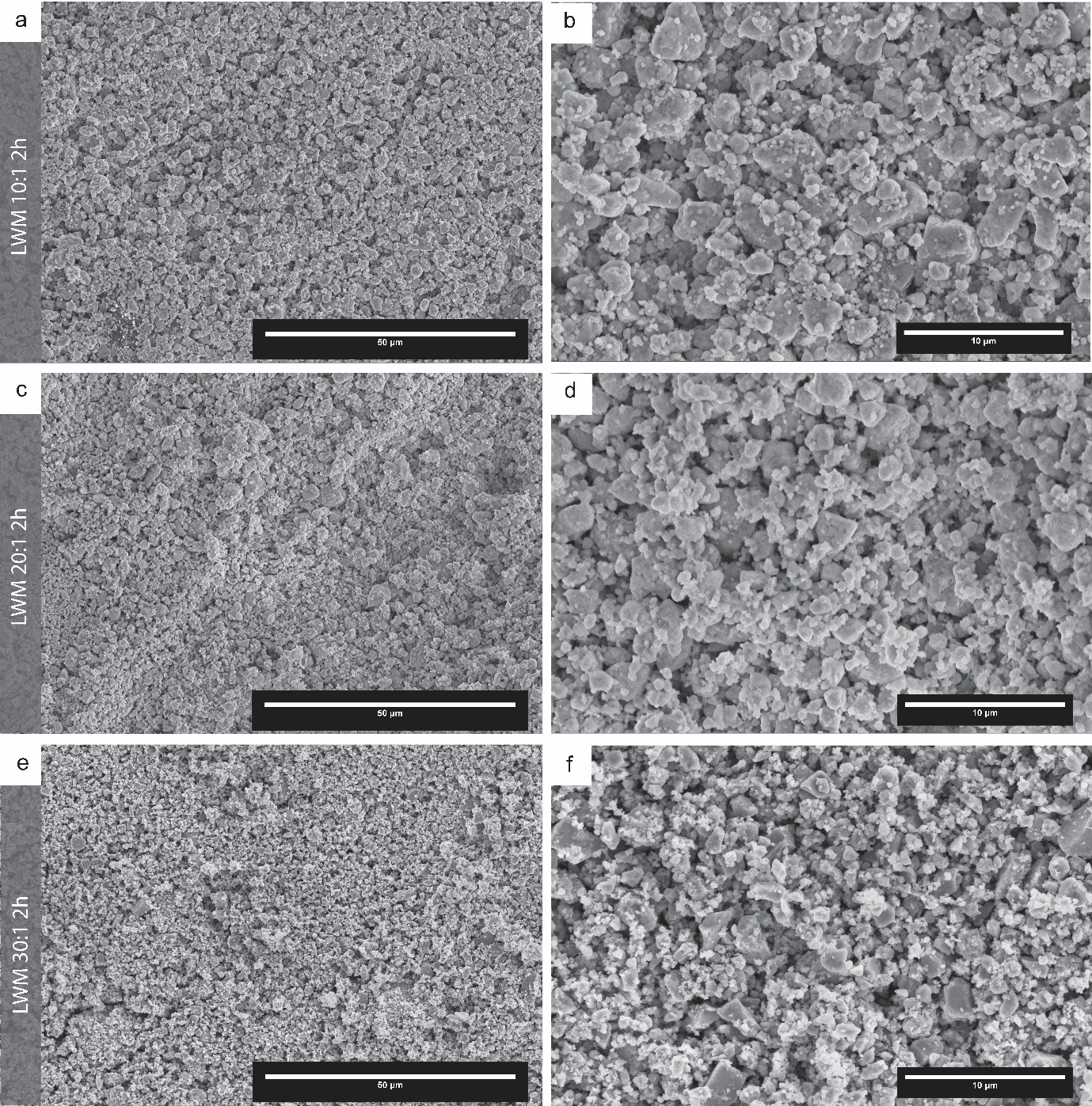


**Figure S3**. SEM images of LPSCl powder after low-energy dry milling (L) at 600 rpm for 10 hours with ball-to-powder ratios (BPR) of 10:1, 20:1, and 30:1, illustrating the influence of BPR used during LWM on the particle size distribution of the particles.


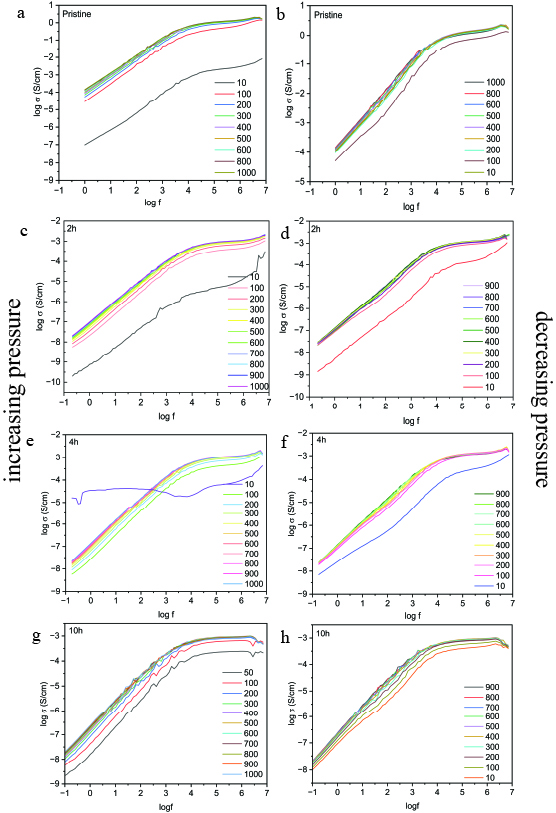


**Figure S4.** Bode plot (log σ vs. log f) derived from EIS measurements for (a,b) pristine, (c,d) 2h, (e,f) 4h and (g,h) 10h milled sample. Electrical processes, which appear as semicircles in Nyquist plots (Z′′ vs. Z′), manifest as plateaus in the corresponding conductivity isotherms. The electrolyte conductivity is extracted from the turning point of the plateau region. For 4h and 10h milled sample, LPSCl particle size is in nano-range giving thinner tapes which leads due poor electrode contact with plunger.

**
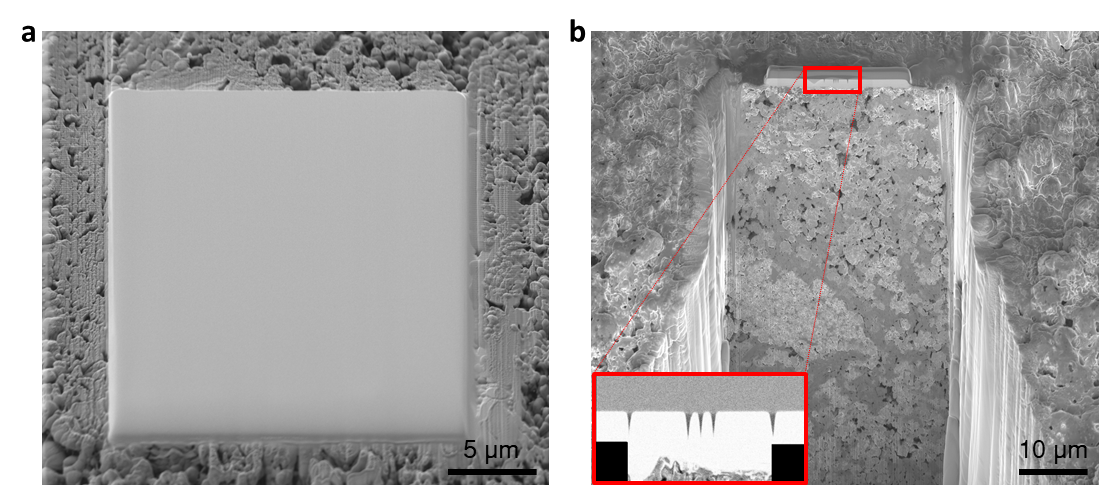

Figure S5.** (a) Sample surface with deposited protective platinum (Pt) layer. (b) FIB-cut cross-section with the Pt + C layer on top. The red box inset highlights the tracking marker within the Pt layer. The scalebars correspond to 5 µm and 10 µm, respectively.

**
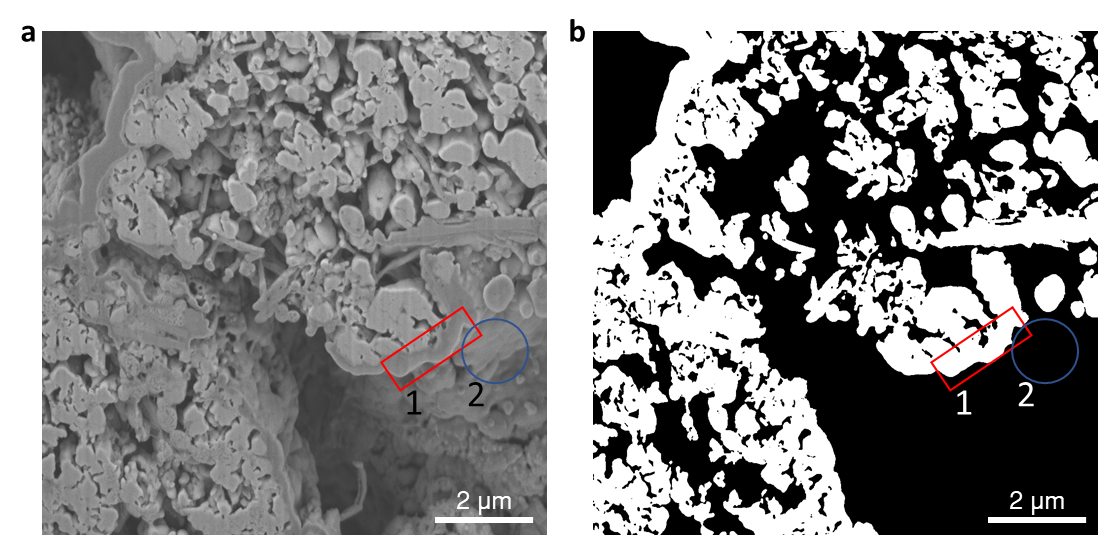
**
**Figure S6.** (a) Secondary electron FESEM image (1024x1024 pixels) and (b) it’s corresponding training mask used for training of the model. Black labelled areas represent pore areas (background regions) and white the material phase. The red box (1) exemplarily shows a material phase region which exhibits the same grey value as the background highlighted within the blue square (2). The scalebars correspond to 2 µm.

**
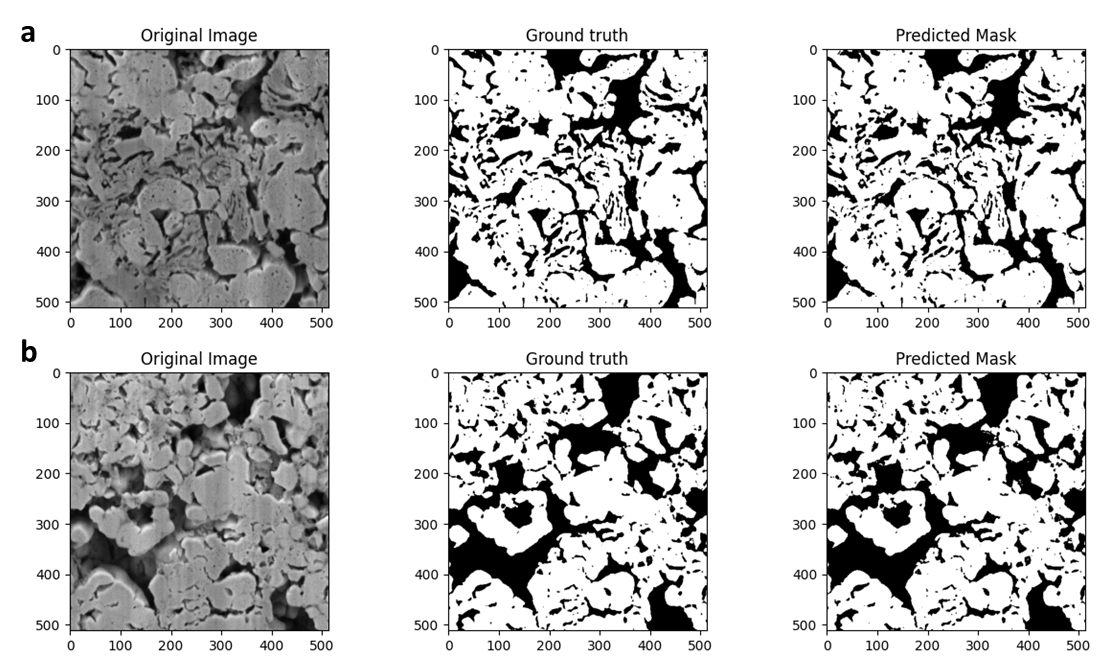
**

**Figure S7.** Original FESEM images, manually created masks (ground truth) and ML-predicted masks (from left to right). Black areas represent pore regions (background regions) and white areas the material phase (foreground). The individual accuracies are 97.09% and 99.01% for the predicted mask for a and b, respectively. The axis unit represents pixel, with one pixel equals 10 nm.

**
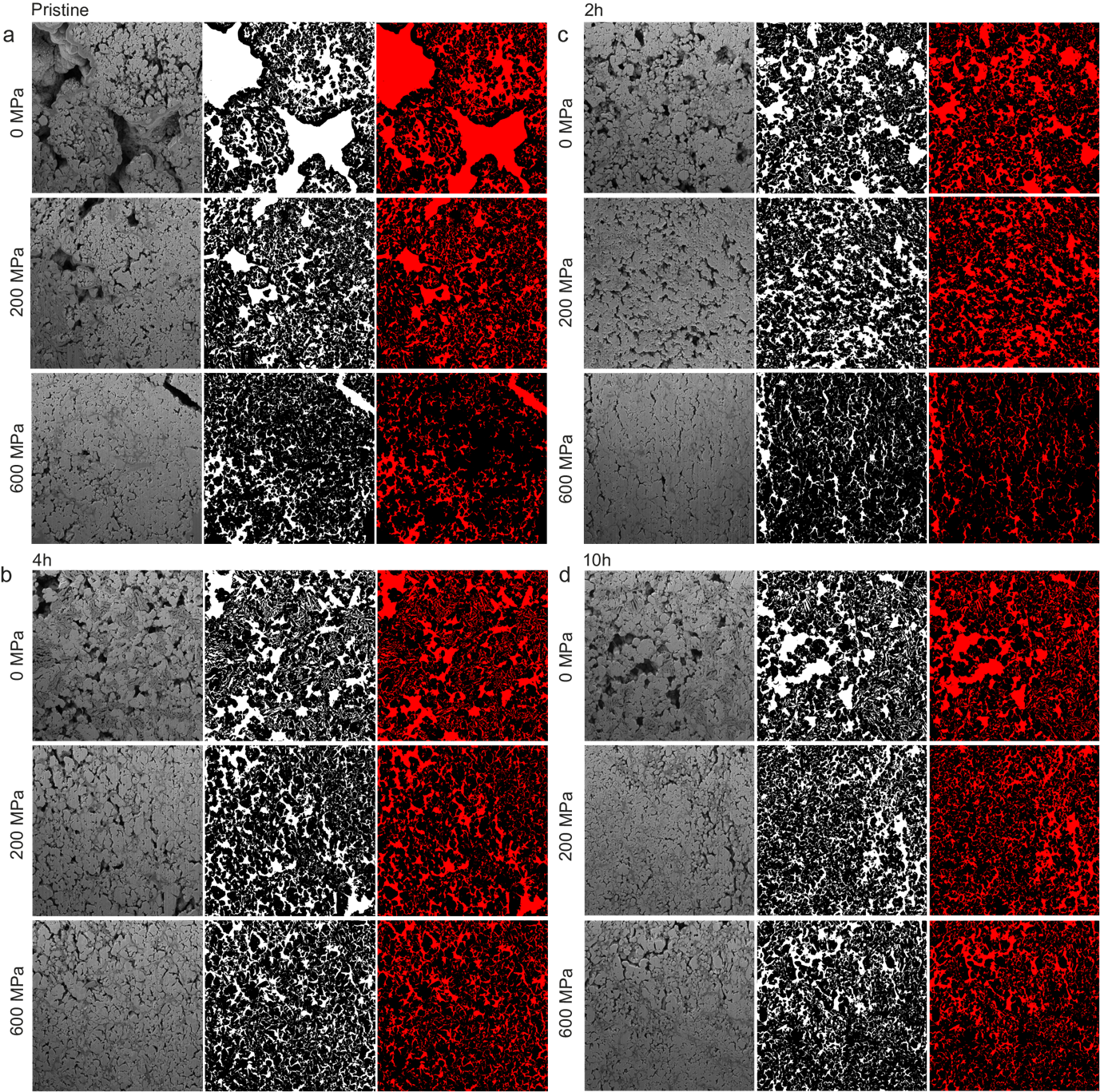
**

**Figure S8.** FIB-SEM micrographs illustrating the microstructural evolution of LPSCl solid-state electrolyte under varying pressures (0 MPa, 200 MPa, 600 MPa) and milling durations (Pristine, 2 h, 4 h, 10 h) (a–d). Each set includes FESEM images (gray), phase-segmented visualizations (middle: black – LPSCl, white – all voids), and connected-void segmentations (right: black – material, red – open, connected pores within the volume of interest). The visualizations reveal progressive densification and a marked reduction in both total and connected porosity with increasing pressure and milling time. Notably, the pristine LPSCl sample at 0 MPa exhibits a significantly higher void fraction, highlighting the effect of mechanical processing and pressure on the microstructure.


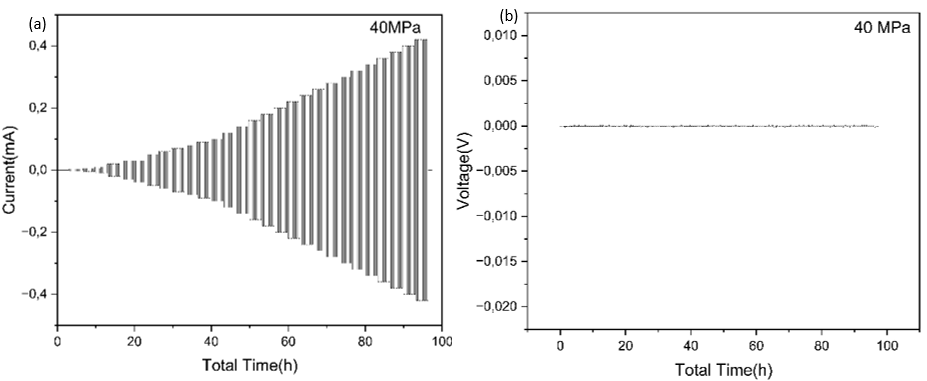


**Figure S9.** Critical current density (CCD) evaluation of a Li|LPSCl|Li cell assembled with a separator pre-densified at 200 MPa. (a) Applied current profile and (b) corresponding voltage response. During testing, the separator was sandwiched between lithium metal electrodes and subjected to an additional stack pressure of 40 MPa. Immediate short-circuiting occurred upon pressure application, preventing any measurable voltage response under current load—indicating mechanical or structural failure of the separator prior to electrochemical cycling.


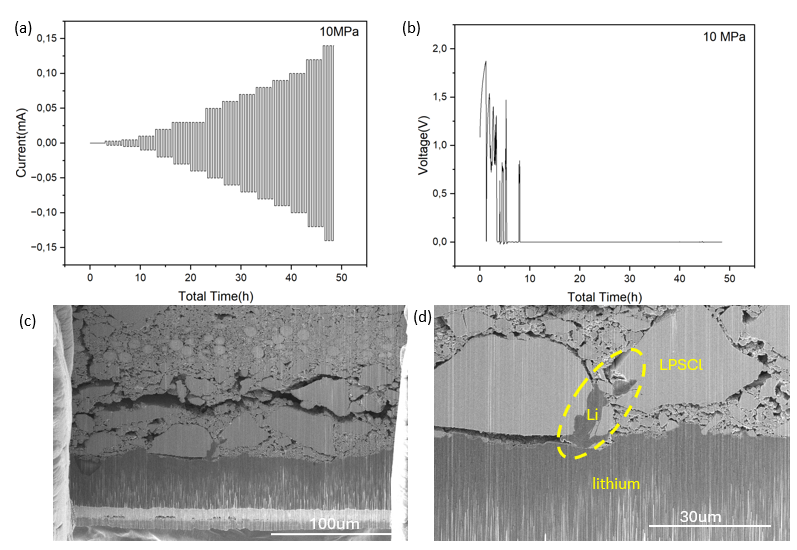


**Figure S10.**Critical current density (CCD) test of a Li|LPSCl|Li symmetric cell using a separator pre-densified at 200 MPa. (a) Applied current profile and (b) corresponding voltage response. After assembling the cell with lithium metal electrodes, a stack pressure of 10 MPa was applied. The cell exhibits an unstable and noisy voltage signal initially, followed by short-circuiting after approximately 6 hours of operation. (c) PFIB-SEM cross-section of the Li|LPSCl interface after testing under 10 MPa stack pressure, revealing interfacial degradation and possible filament growth. (d) Higher magnification image of the region marked in (c), highlighting morphological features at the failure site.


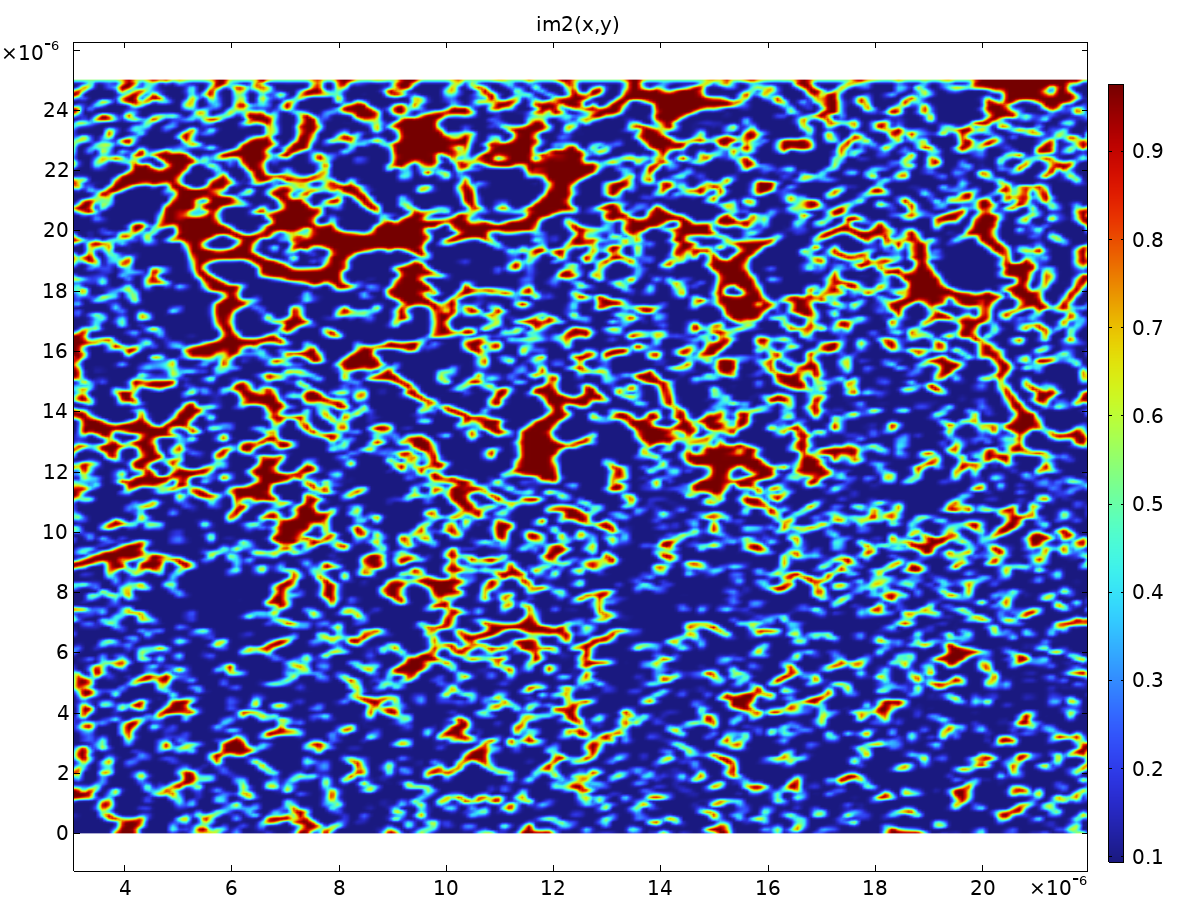
+

Sampling line

**Figure S11.** A typical SEM image of the solid electrolyte used to determine the morphology of the interface. Here, the relative depth of each point on the surface is correlated with colours shown in label. The void and matrix are represented by red and blue, respectively. A sampling line is assumed, and the interface is constructed based on the areas intersecting the line.


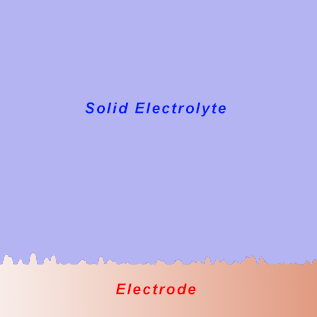


**Figure S12.**  Schematic representation of the system comprising the solid electrolyte (SE) and electrode. The rough SE/electrode interface is the focus in the modelling study.


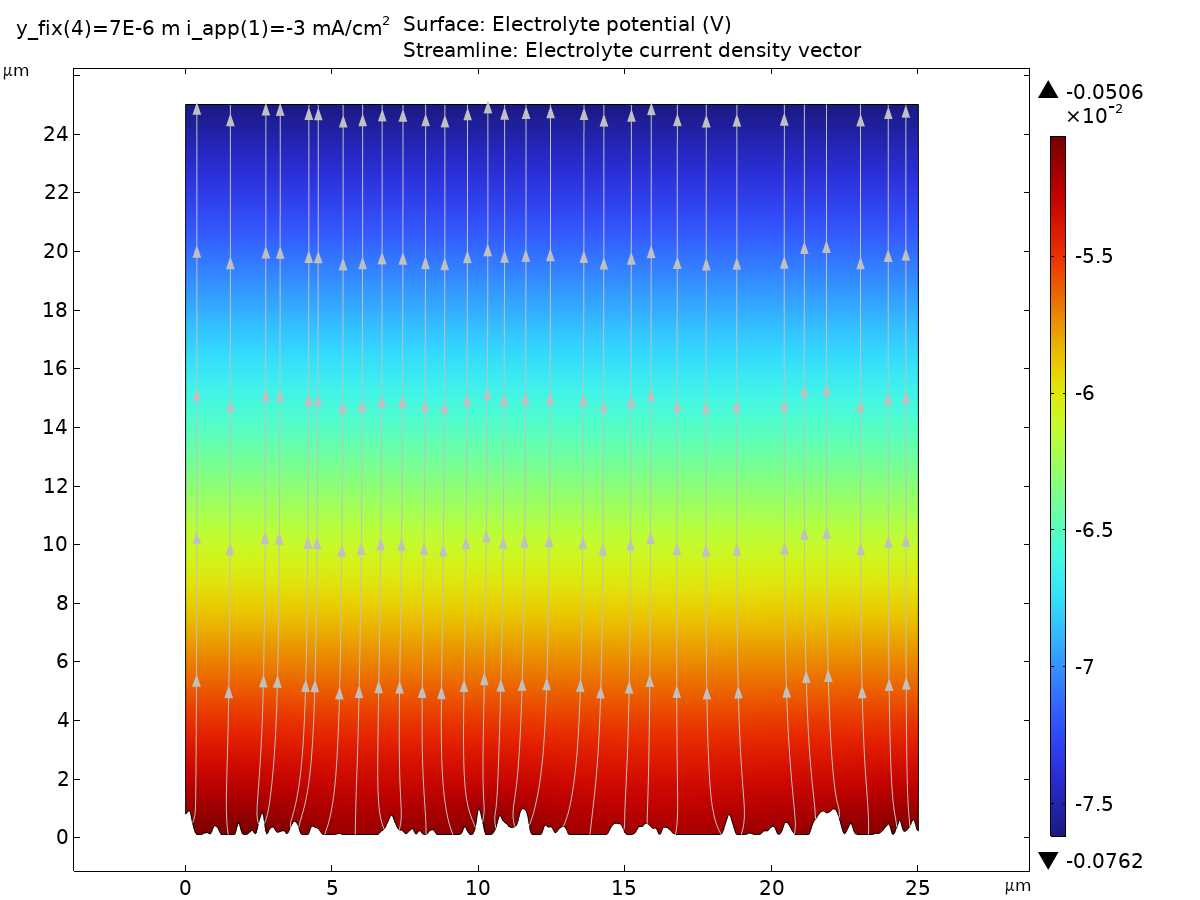


**Figure S13.** Electric potential distribution in the SSE under an applied current density of i_app_=-3 mA/cm^2^. The current streamlines exhibit distortion near the roughened interface, highlighting the influence of interface morphology on the electric potential.

**Figure S14.** Distribution of reaction current density (i_BV_ in Eq.2) for a typical sample. The maximum and minimum current densities are indicated in the figure. The values are used in the next step for the calculation of the current ratio.

| Milling Time (Hrs) | Pressure (MPa) | Reaction current (Butler-Volmer current) at the interface calculated by model for different samples |  |  |
| --- | --- | --- | --- | --- |
| 0 | 0 |  | Increasing the milling time |  |
| 2 | 0 |  |  |  |
| 4 | 0 |  |  |  |
| 10 | 0 |  |  | Increasing the compressional pressure |
| 10 | 200 |  |  |  |
| 10 | 400 |  |  |  |

**Figure S15.** The distribution profiles of the reaction current for samples prepared with various milling times and compressional pressures.

|  |  |
| --- | --- |

**Figure S16.** Ratio of maximum to minimum currents for different samples processed under various milling time and compressional pressure. The applied current is a) i_app_=-0,5 mA/cm^2^, b)i_app_=-3 mA/cm^2^

**
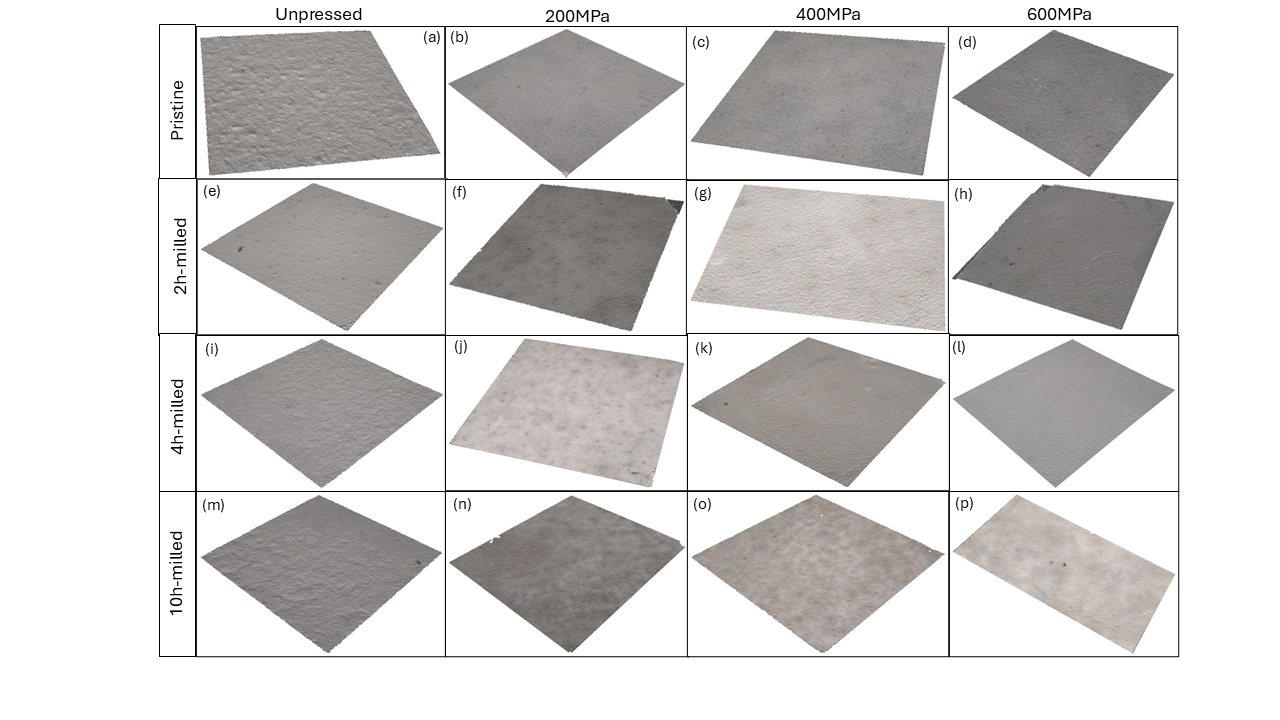
**

**Figure S17.** Optical microscopy images of LPSCl tapes with 200 MPa, 400 MPa and 600 MPa densified and without densification.


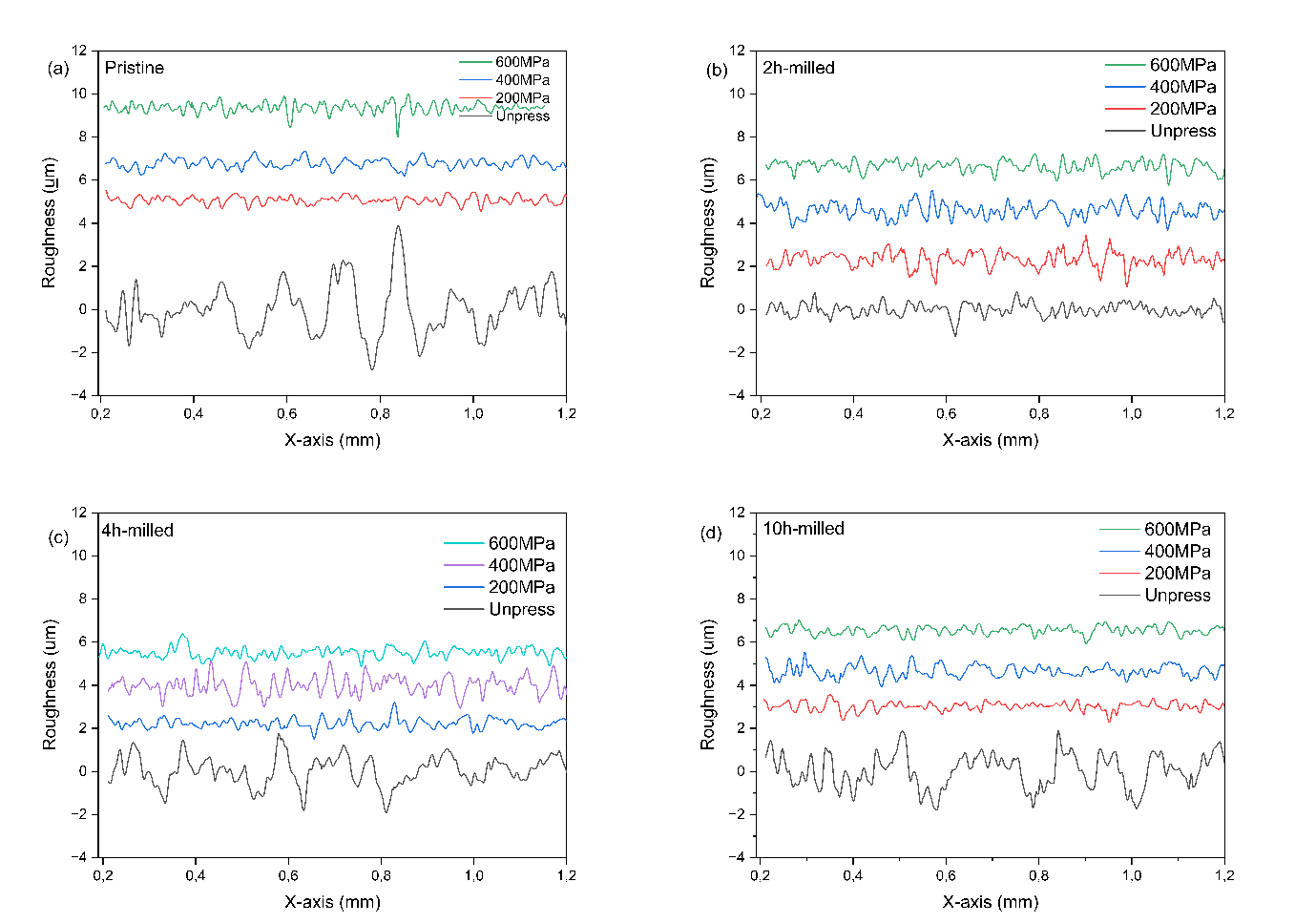


**Figure S18.** Mean roughness values measured along the x-axis for the LPSCl tapes. The pristine, unpressurized sample exhibits the highest roughness (~6 µm), which reduces to approximately 2 µm under a pressure of 600 MPa. For milled particles, the roughness decreases from ~6 µm to nearly 3 µm, and further pressing lowers it to about 1 µm.


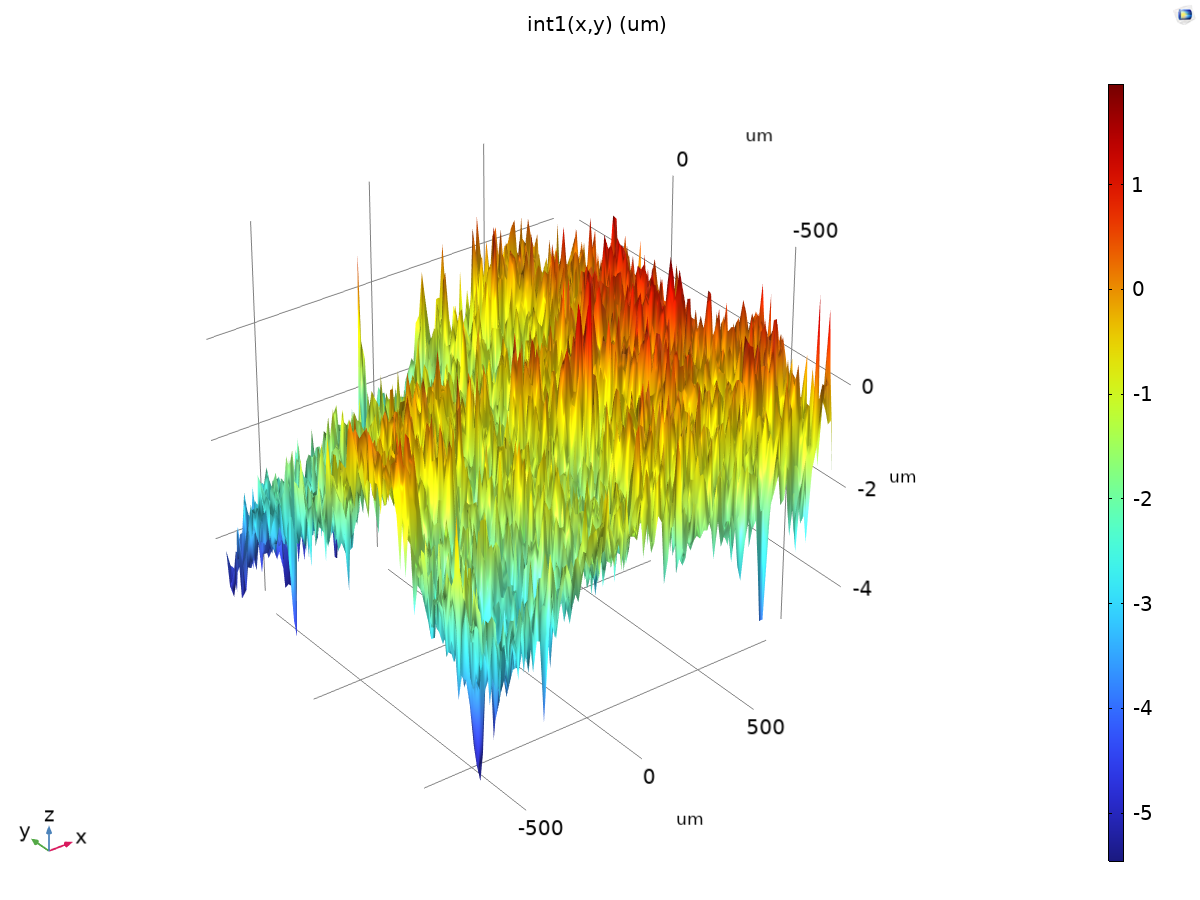


**Figure S19.** A microscopic image illustrating the surface morphology of a sample. The regions with high elevation and depression are depicted by red and blue colours, respectively. While sharp spikes represent local variation of the surface topography, a global inclination towards the edges of the sample (blue areas) can be observed.


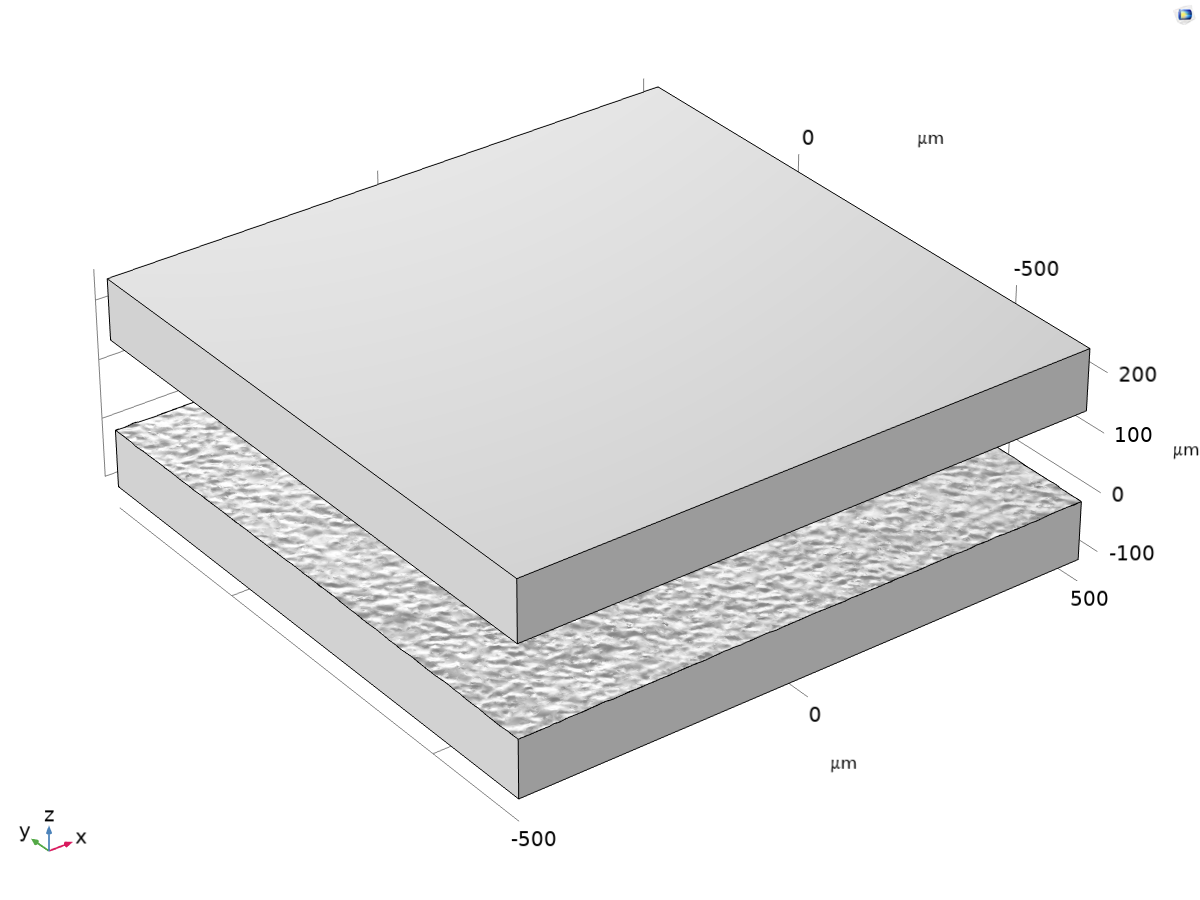


Pressing Block

electrolyte

**Figure S20.** Geometry of electrolyte and pressing block.


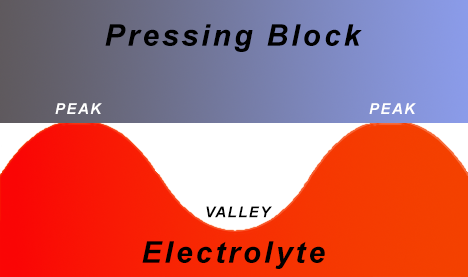


**Figure S21.** A simplified electrolyte block with rough surface being under mechanical stress by a pressing block.

| **Sample** | **Microscopic image** | **Stress distribution (MPa)** | **Sample** | **Microscopic image** | **Stress distribution (MPa)** |
| --- | --- | --- | --- | --- | --- |
| Pristine  0 MPa | 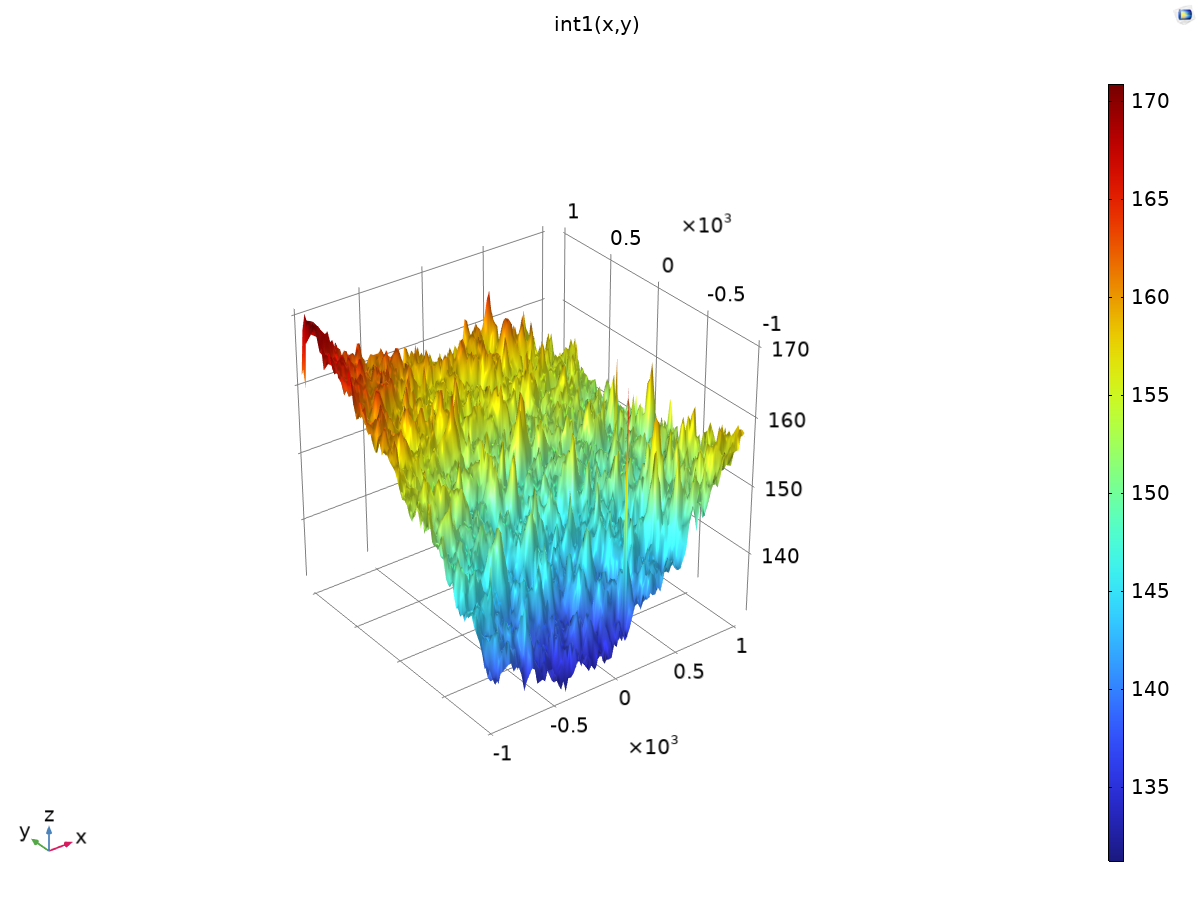 | 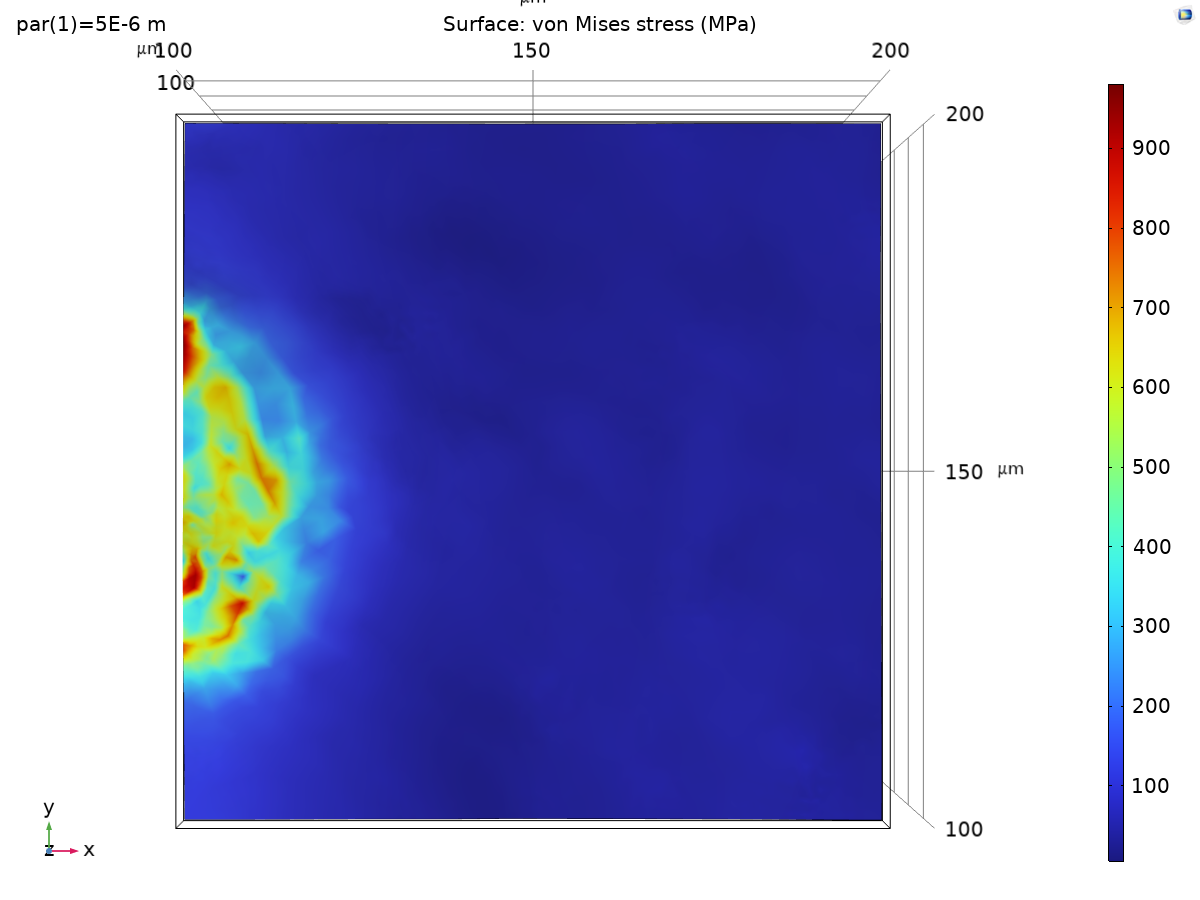 | Pristine  200 MPa | 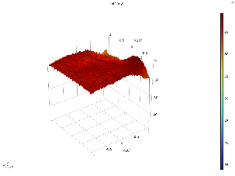 | 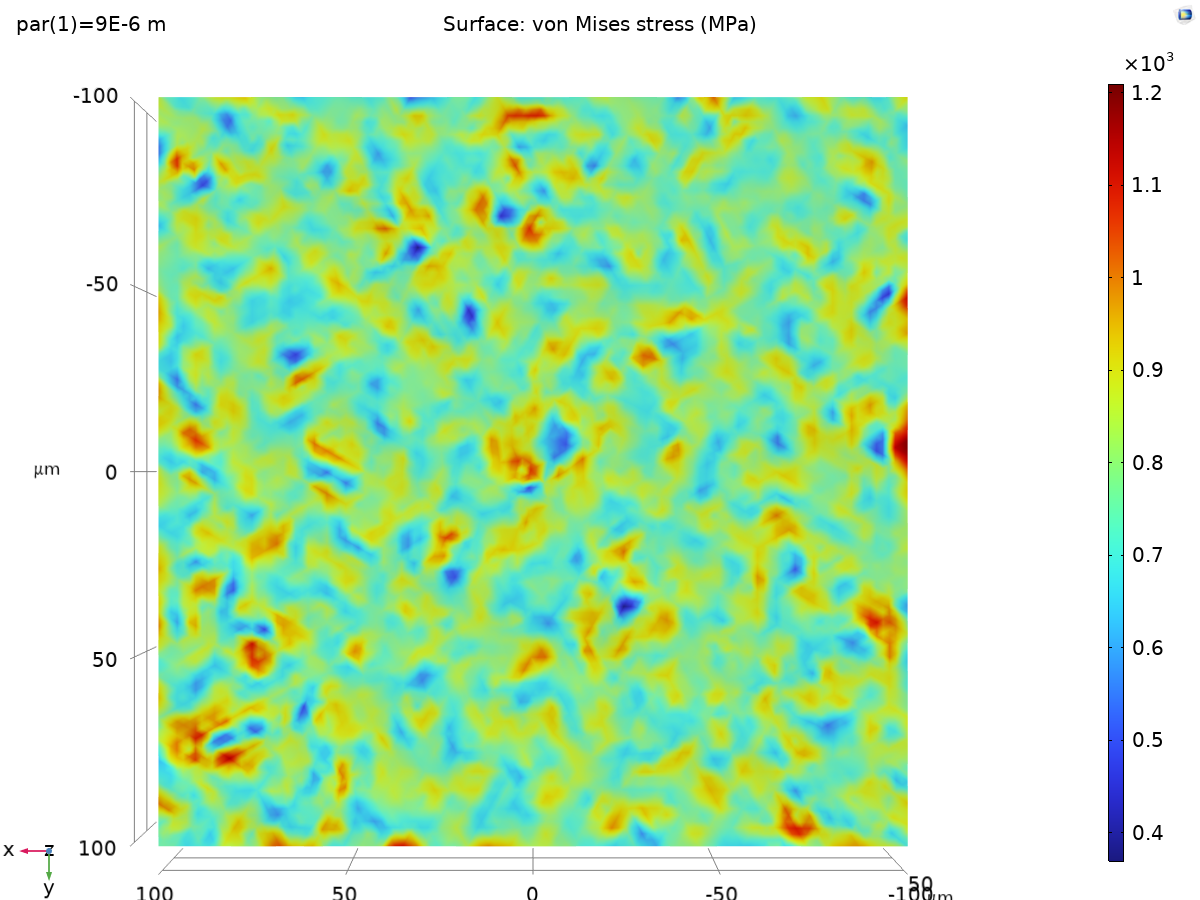 |
| Pristine 400 MPa | 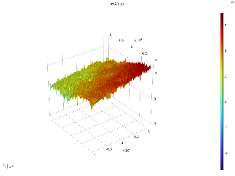 | 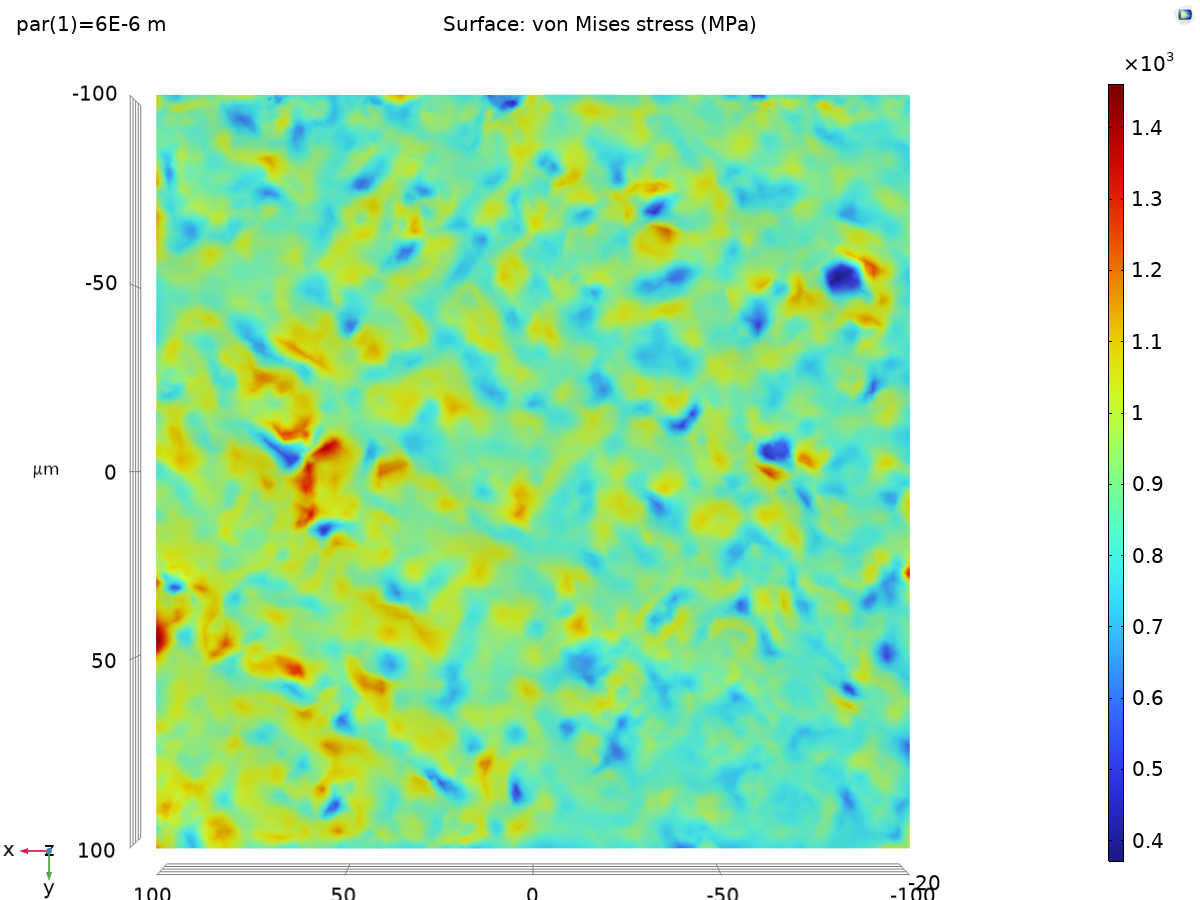 | Pristine 600 MPa | 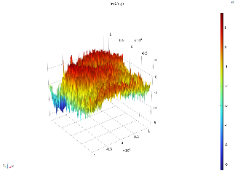 | 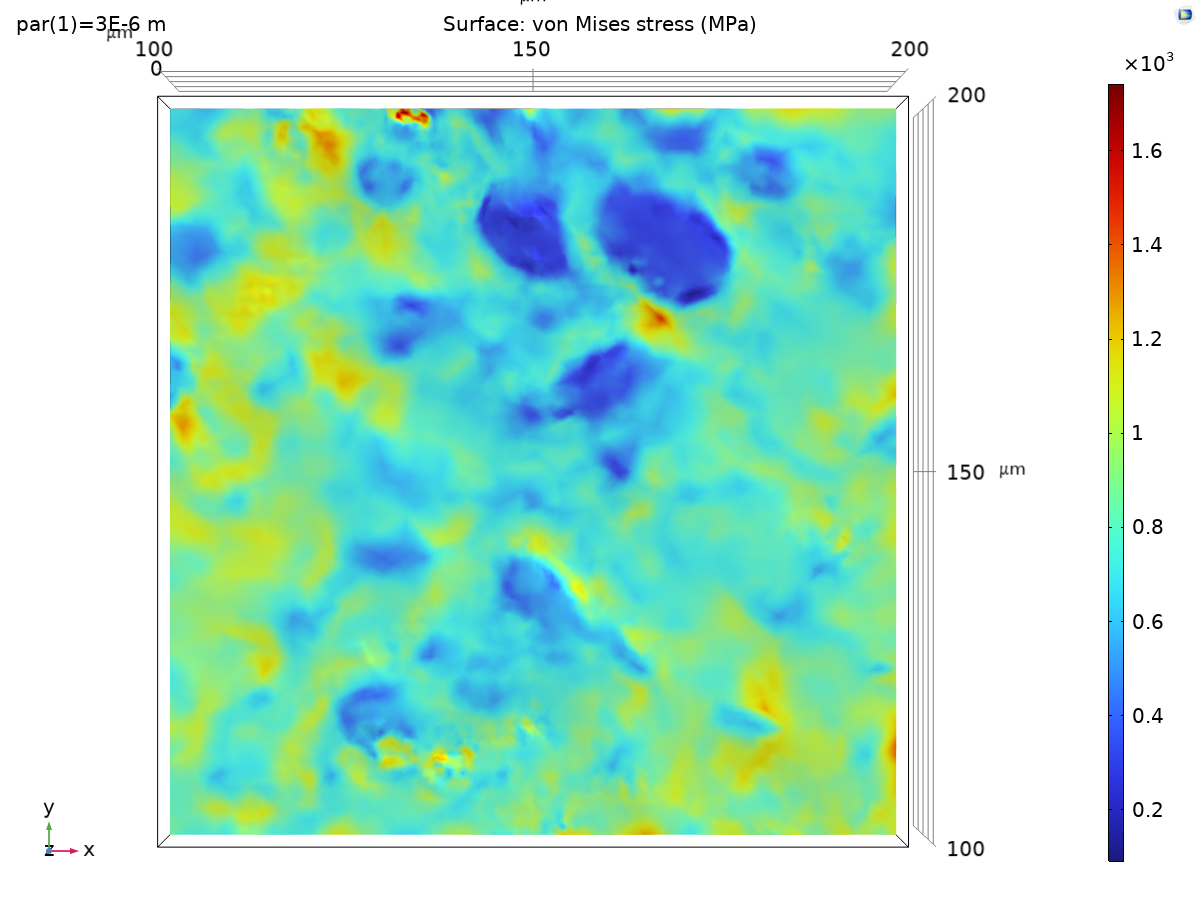 |
| 2hrs  0 MPa | 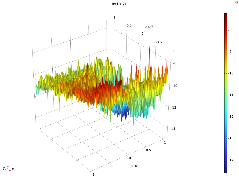 | 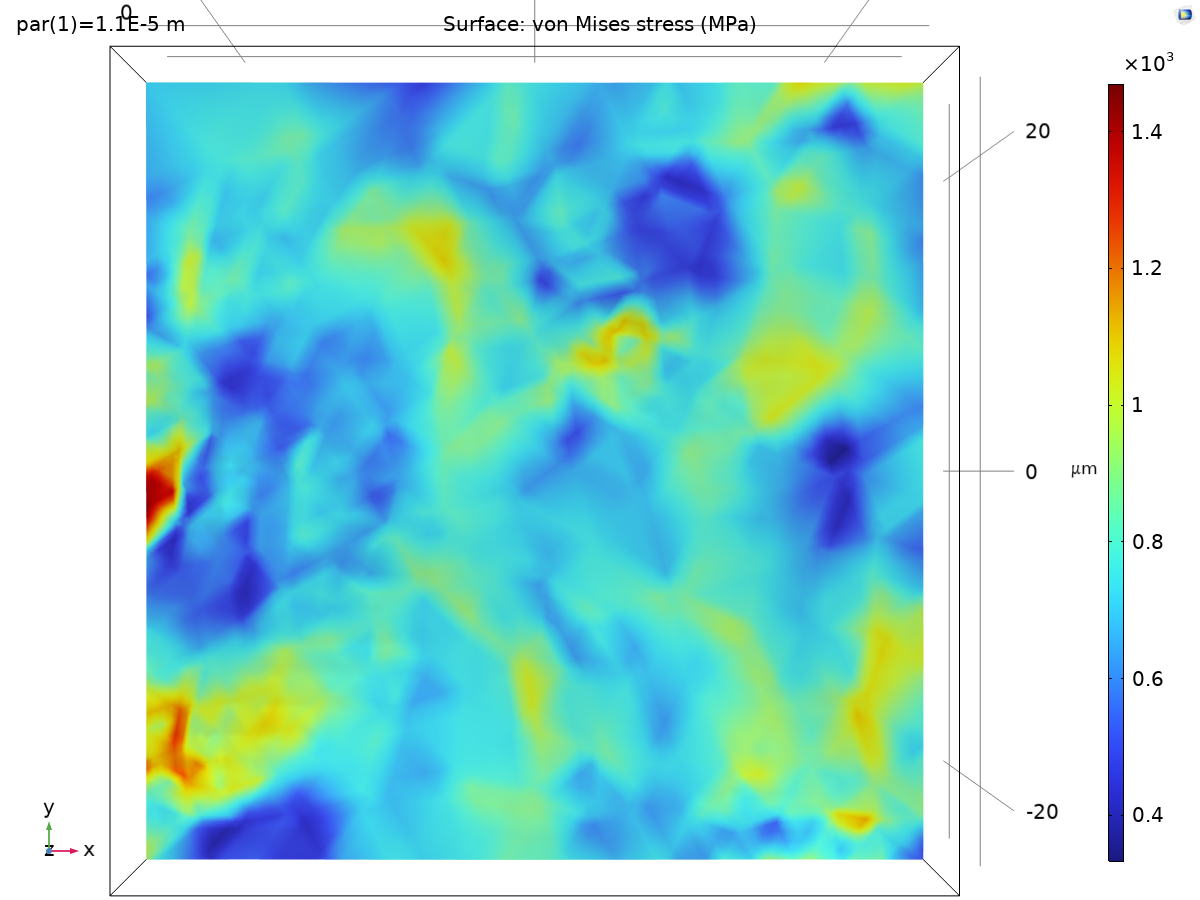 | 2hrs  200 MPa | 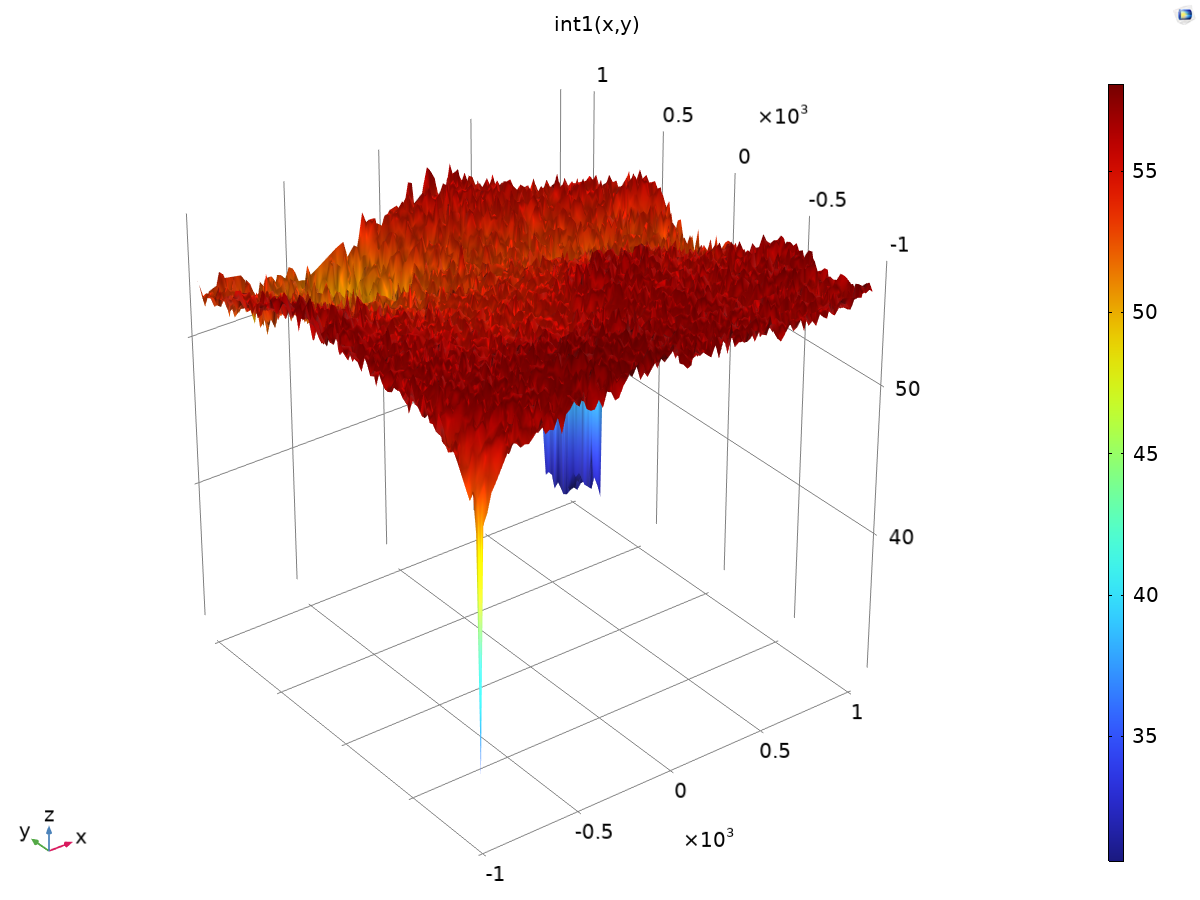 | 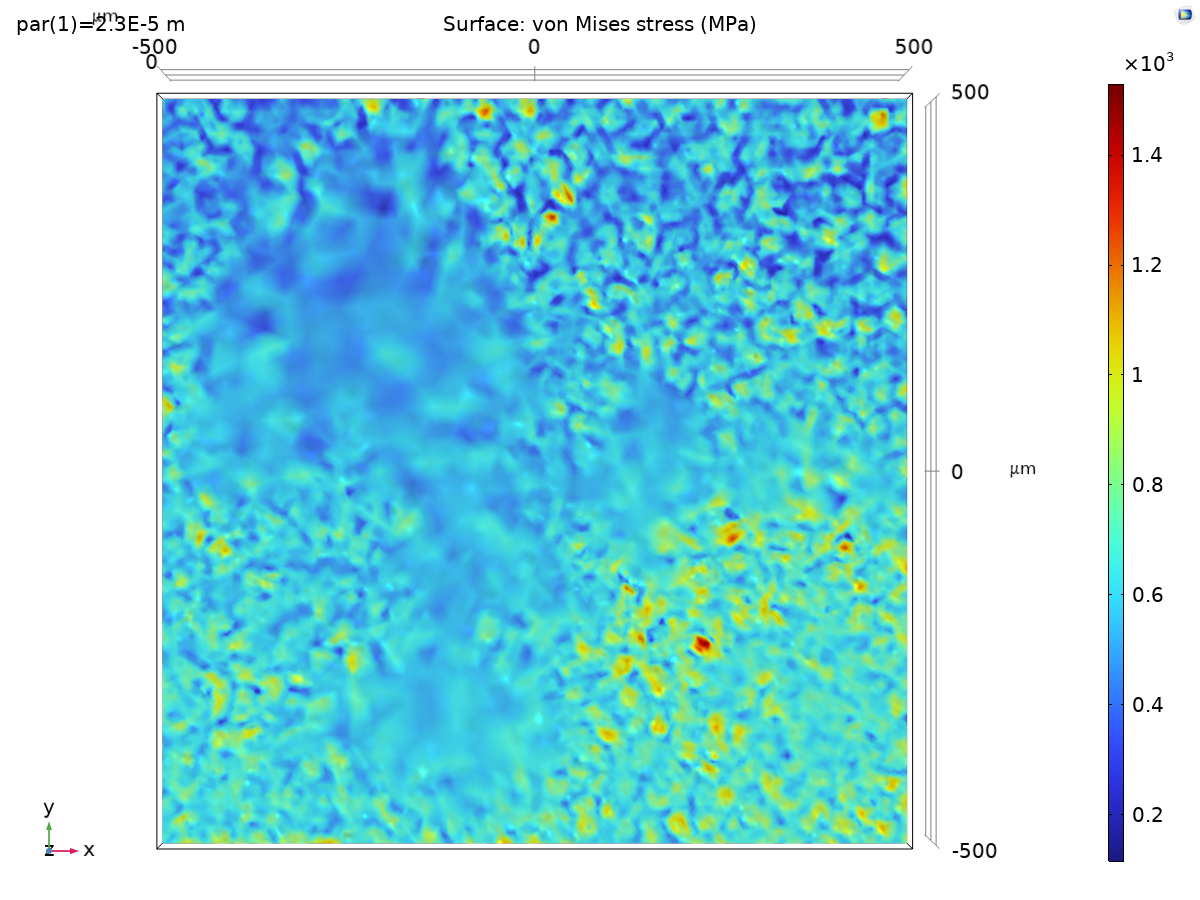 |
| 2hrs  400 MPa | 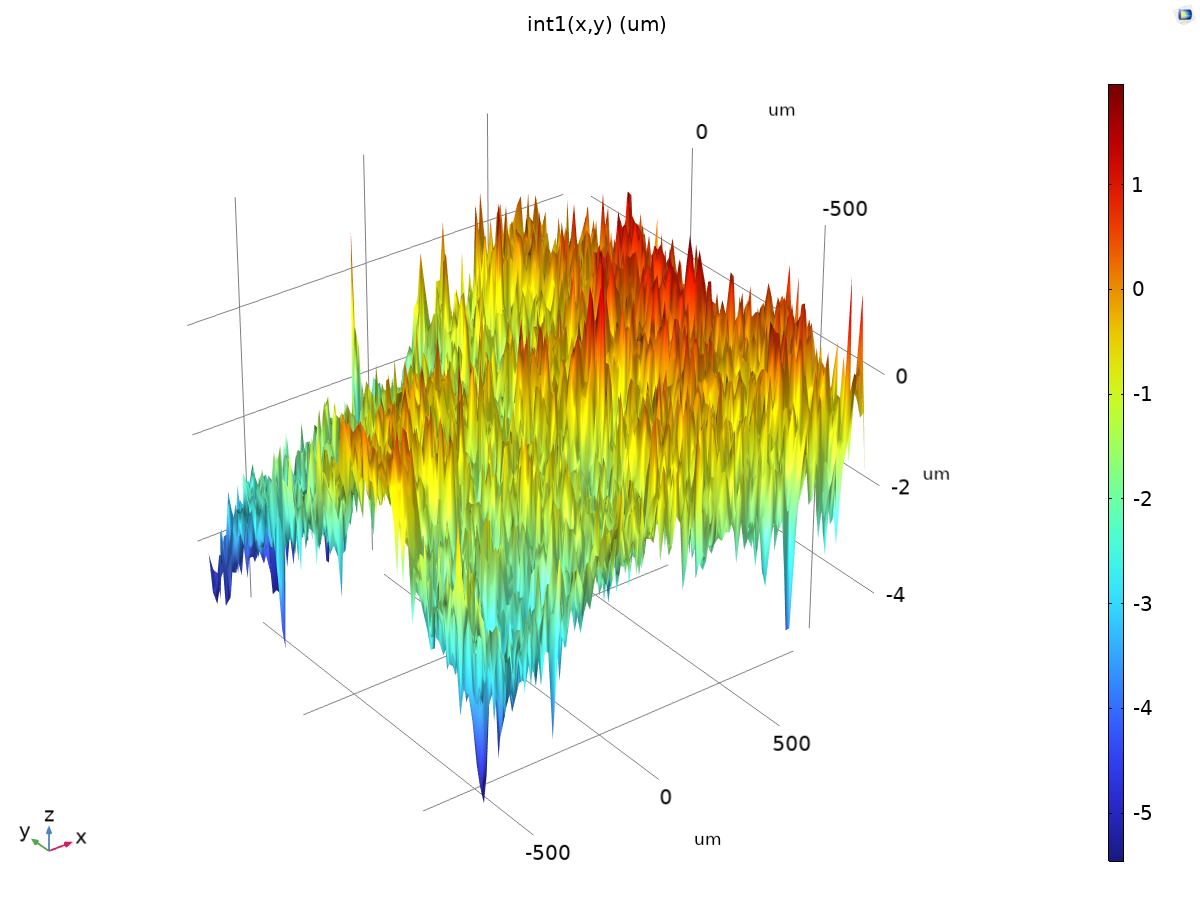 | 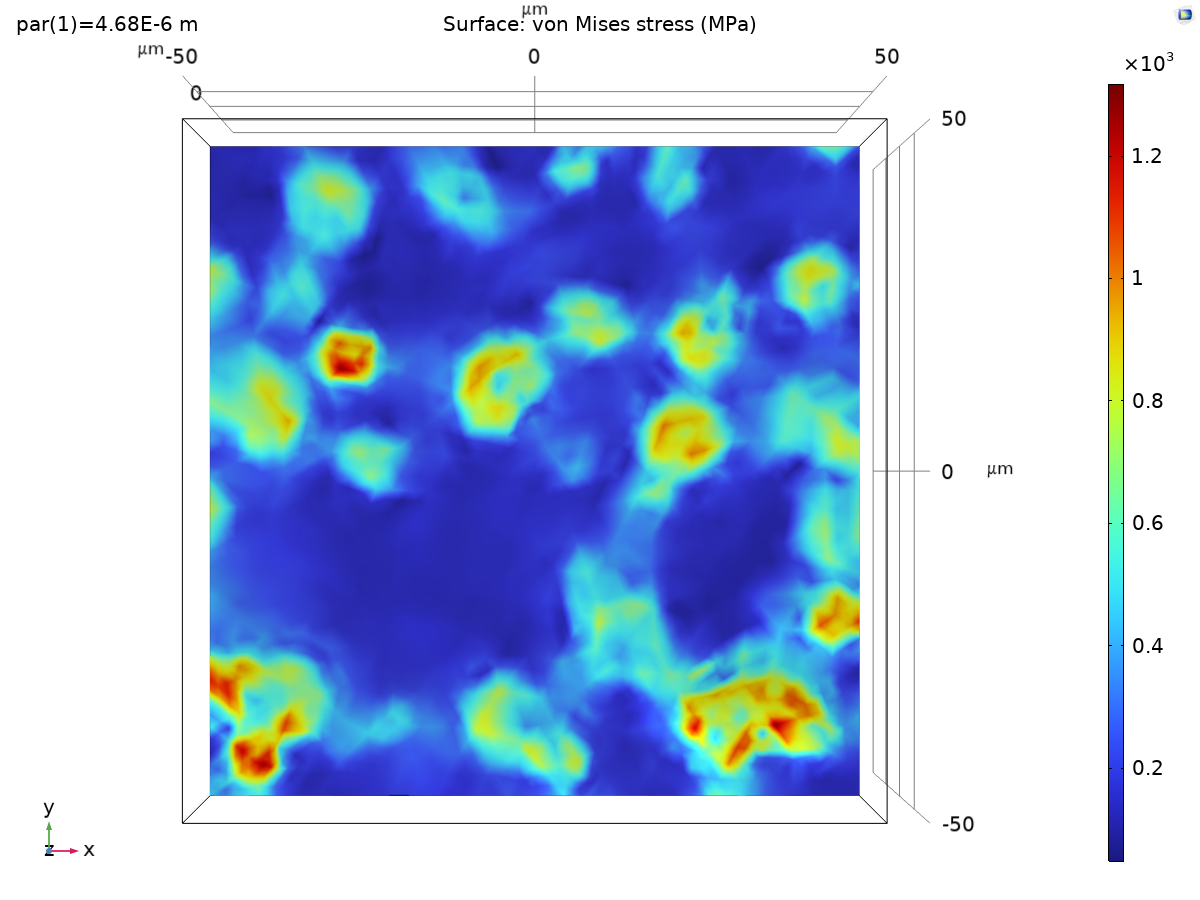 | 2hrs  600 MPa | 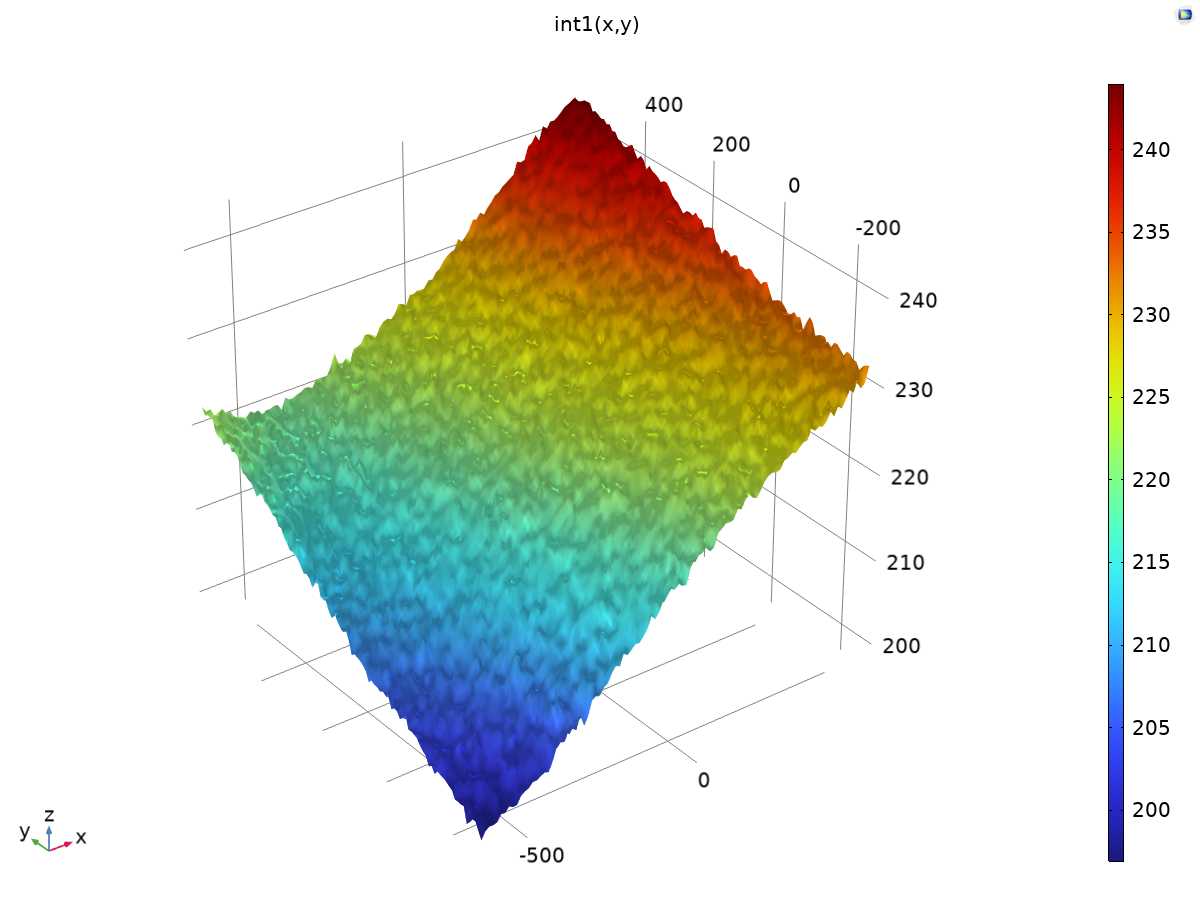 | 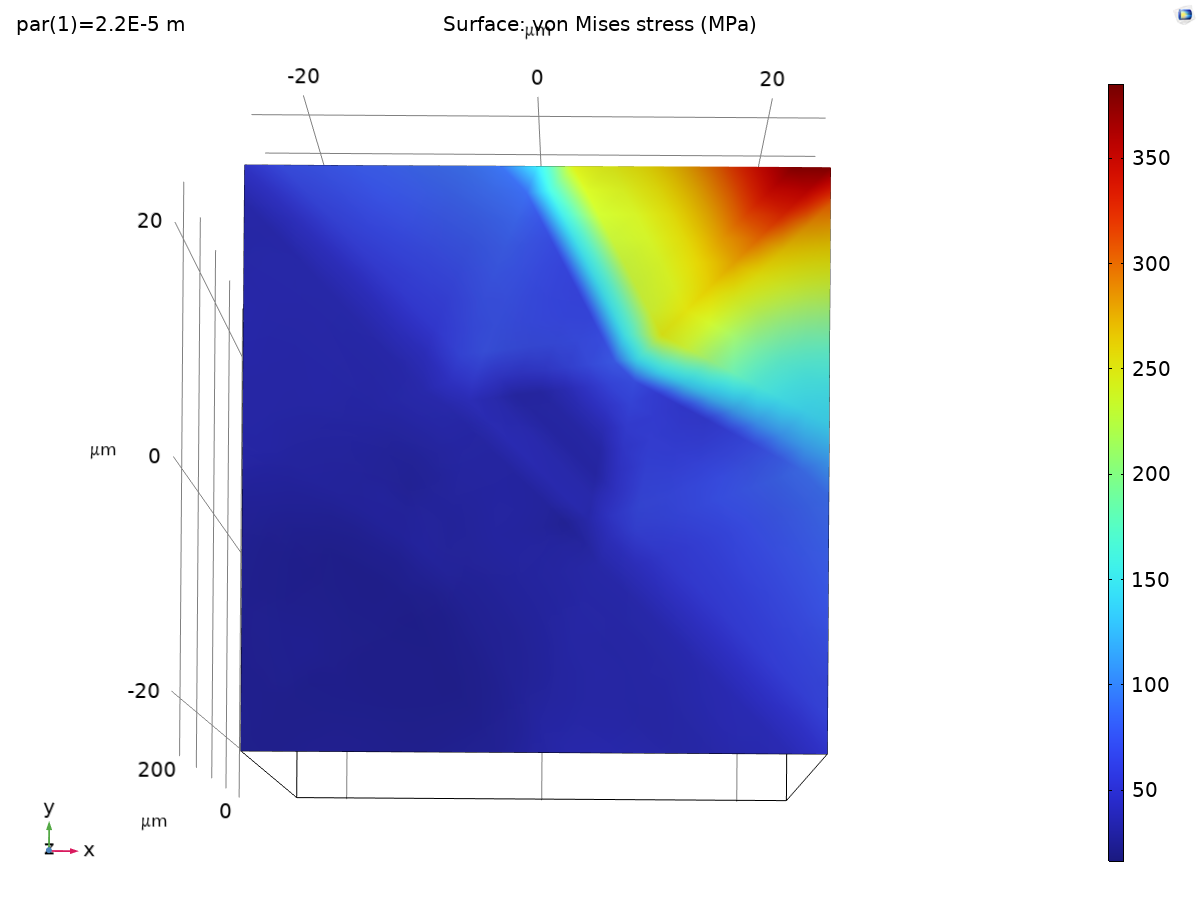 |
| 4hrs  0 MPa | 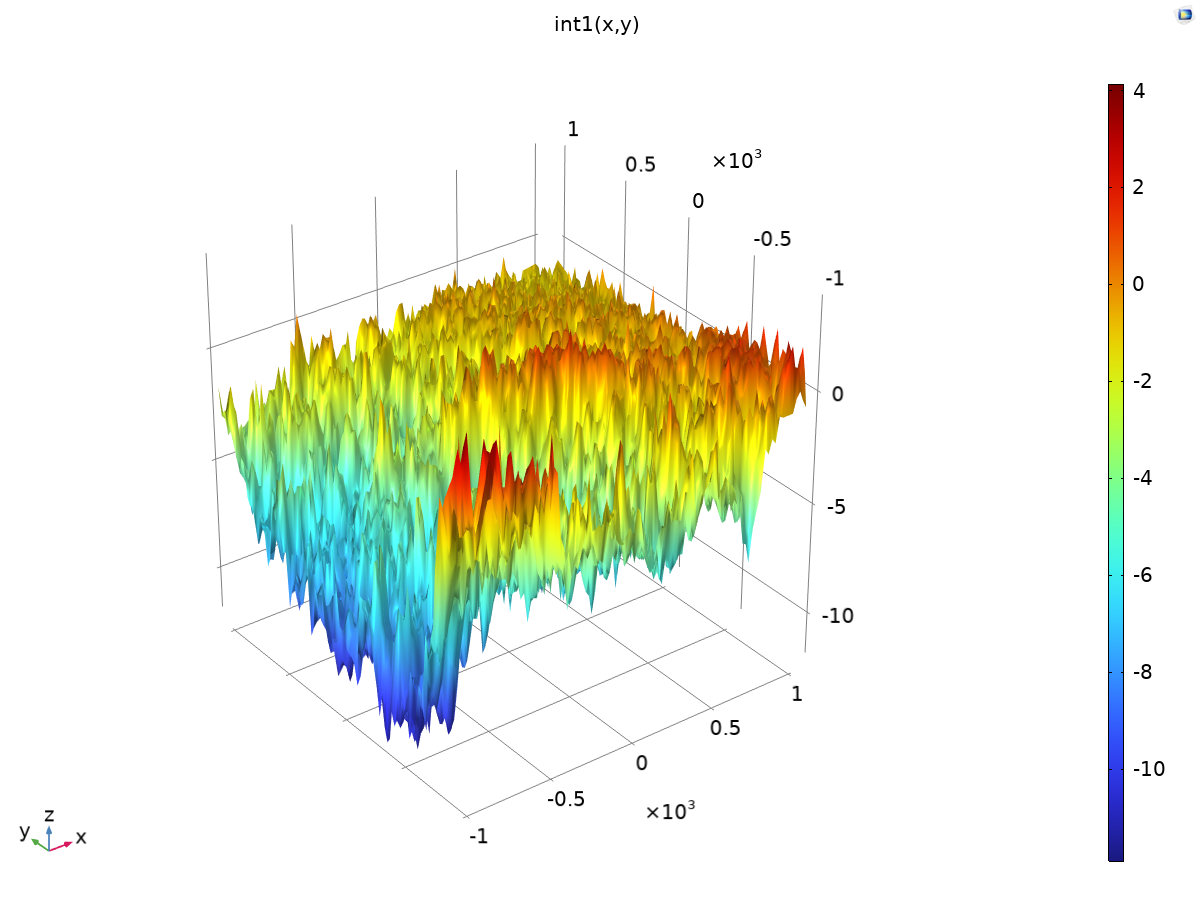 | 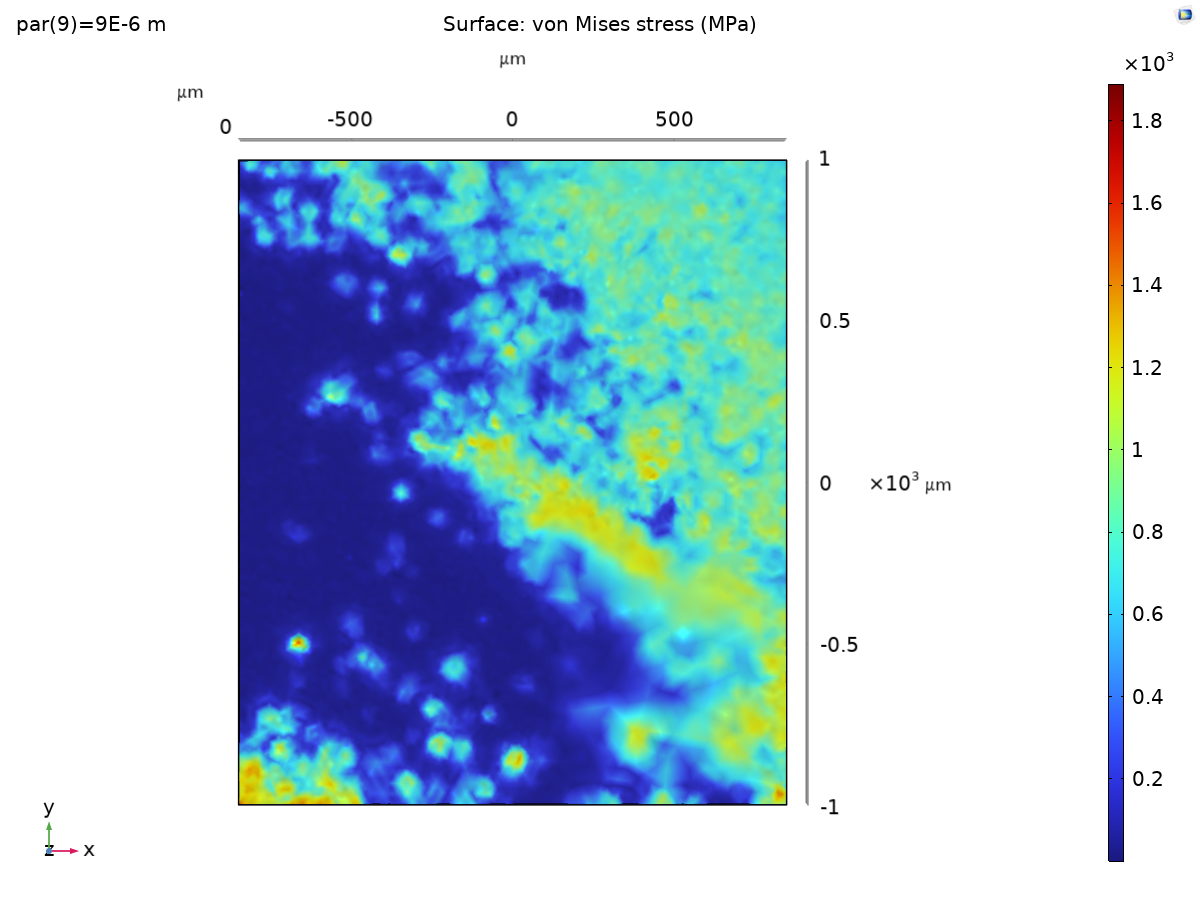 | 4hrs  200 MPa | 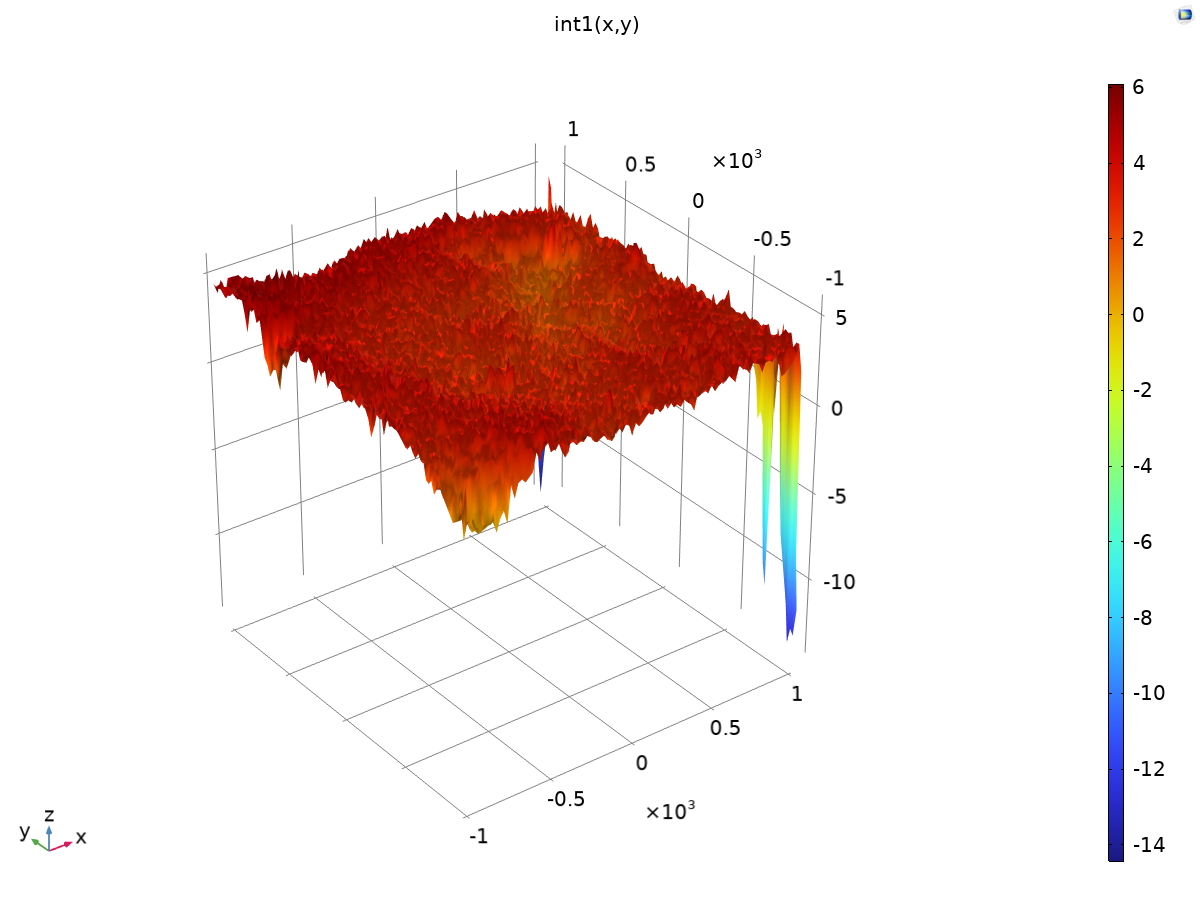 | 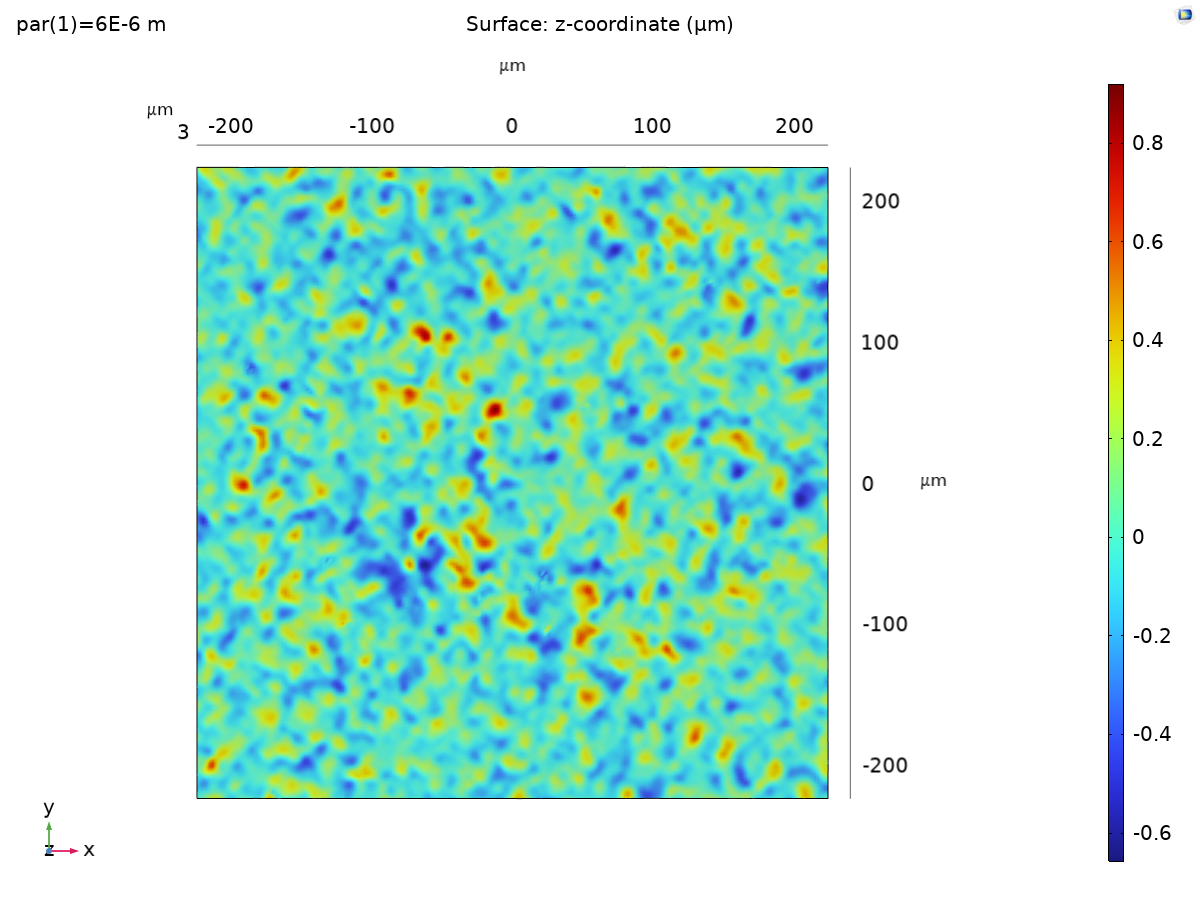 |
| 4hrs  400 MPa | 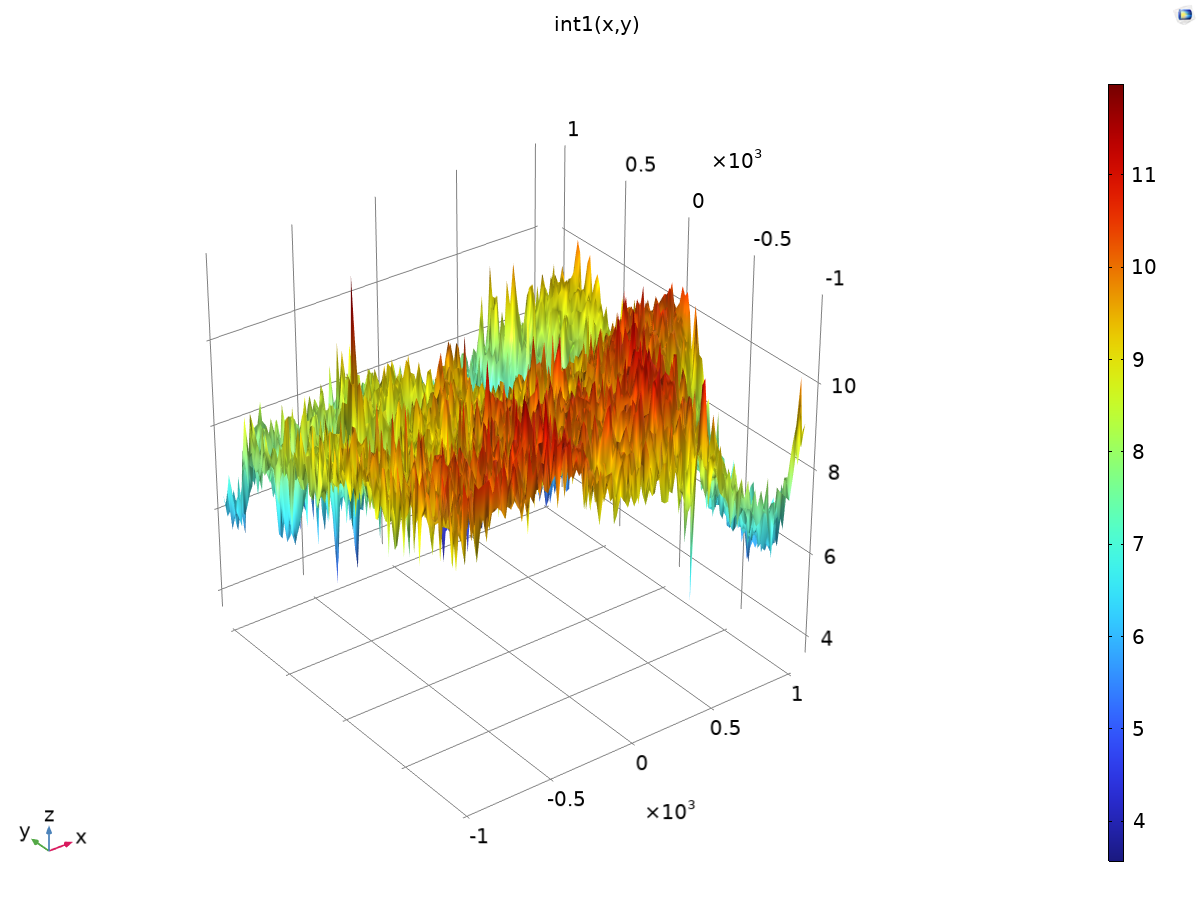 | 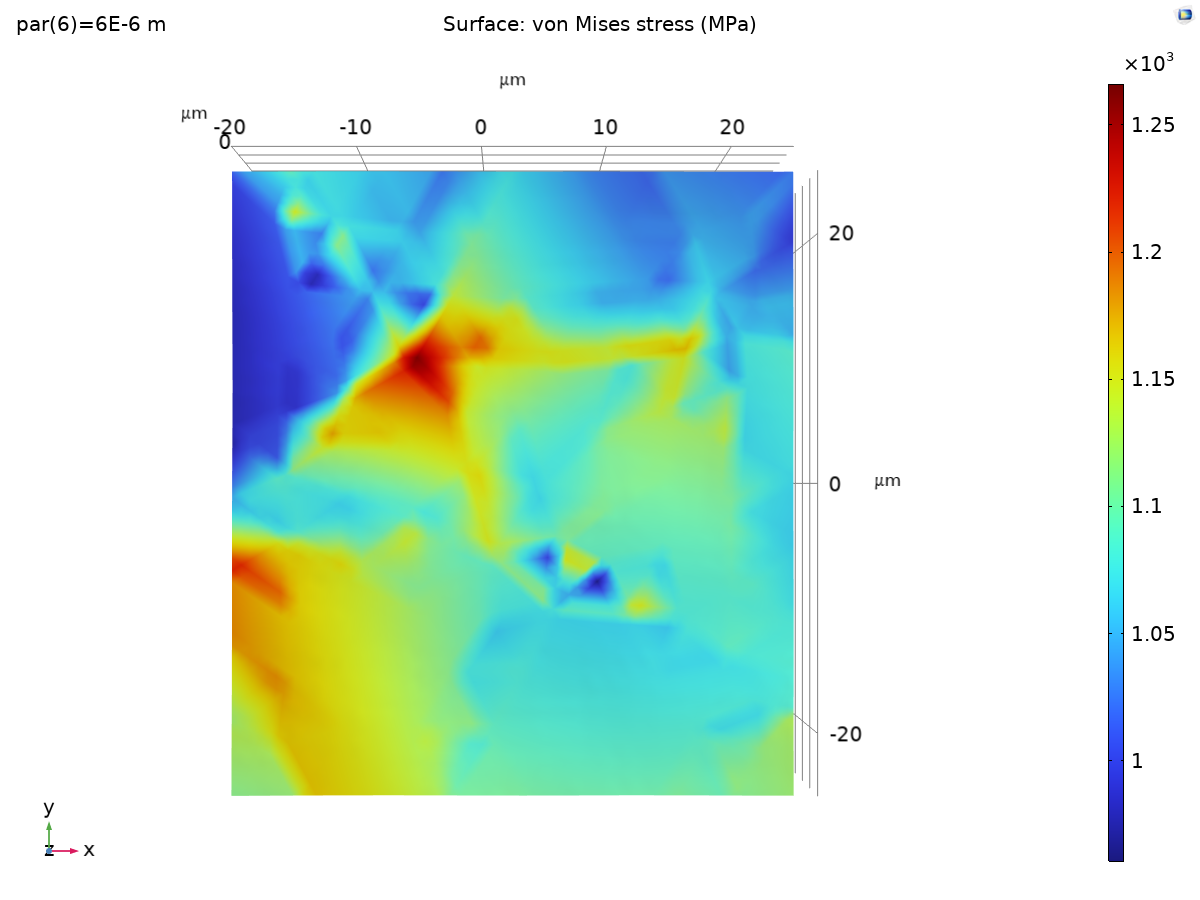 | 4hrs  600 MPa | 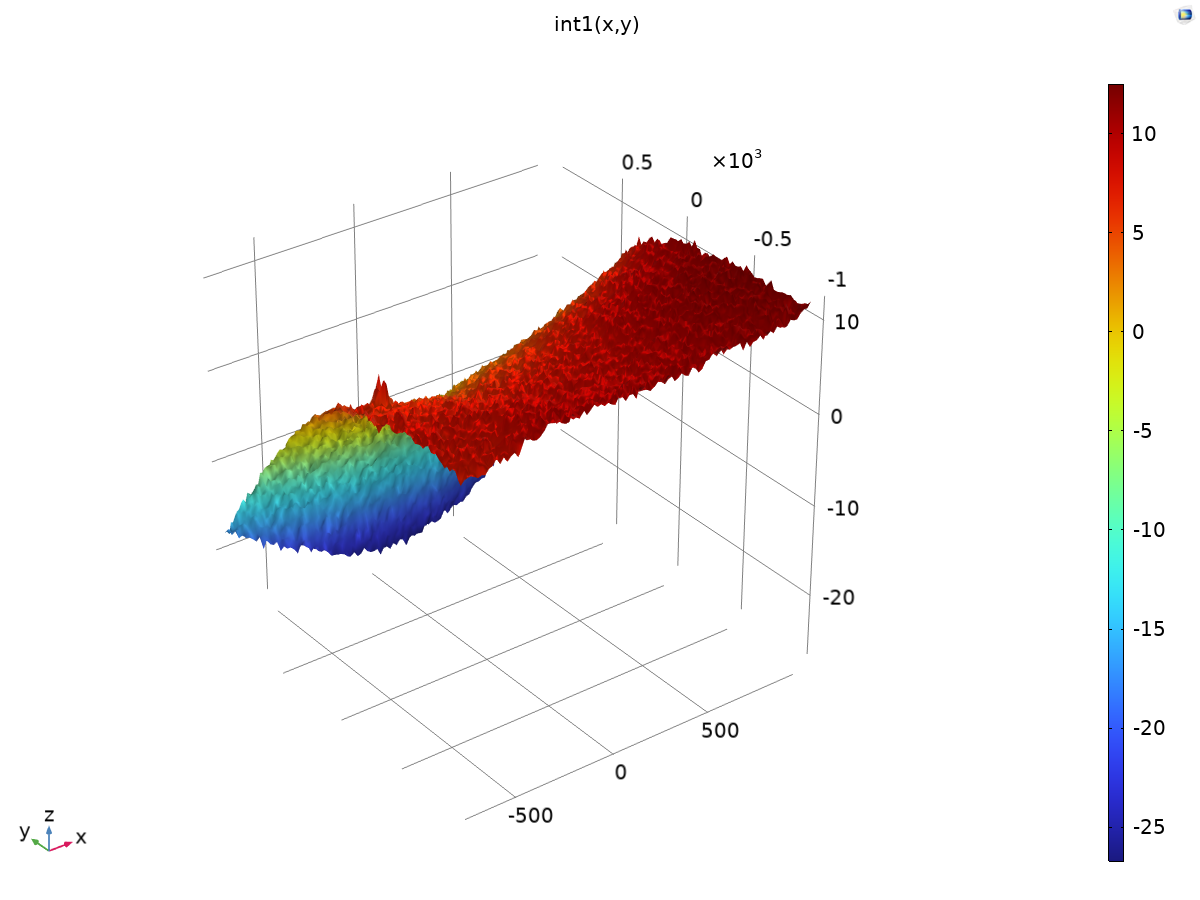 | 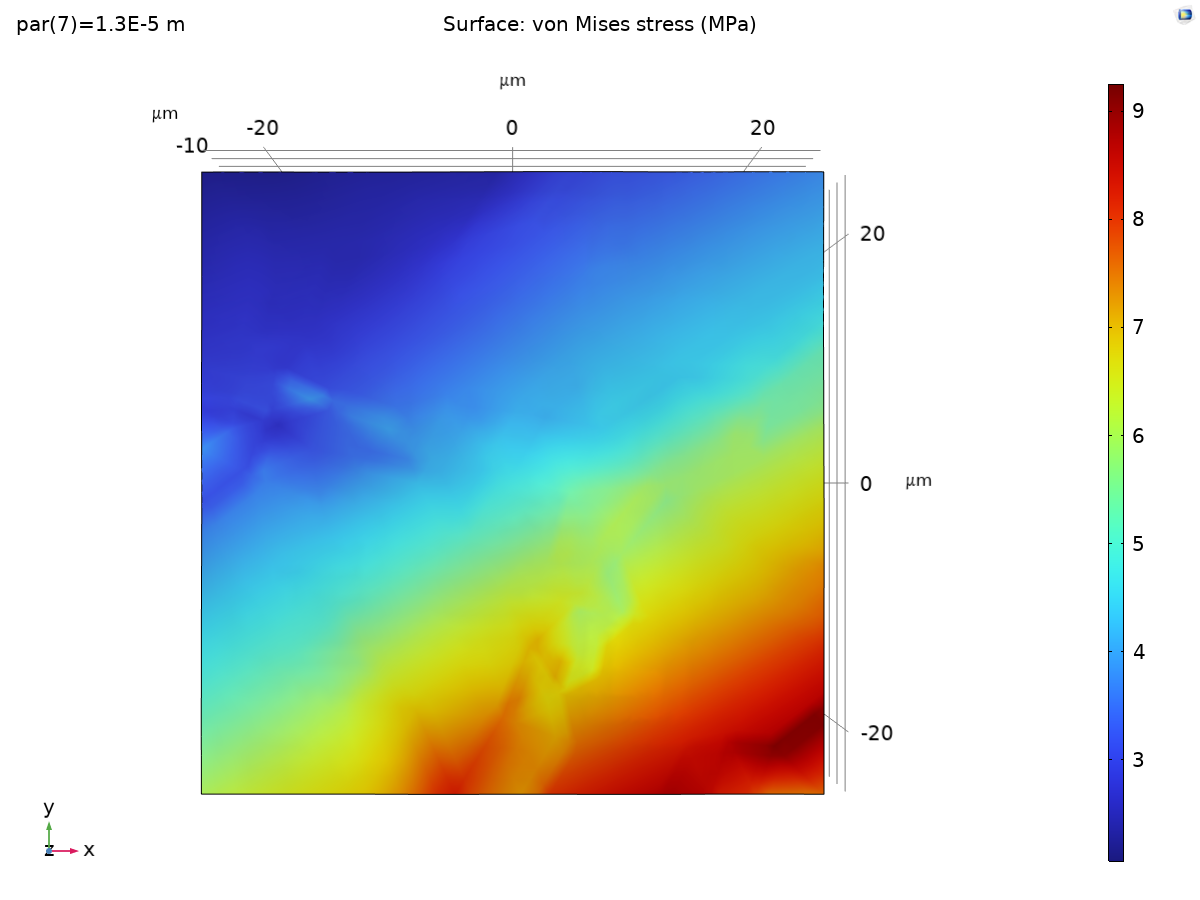 |
| 10hrs  0 MPa | 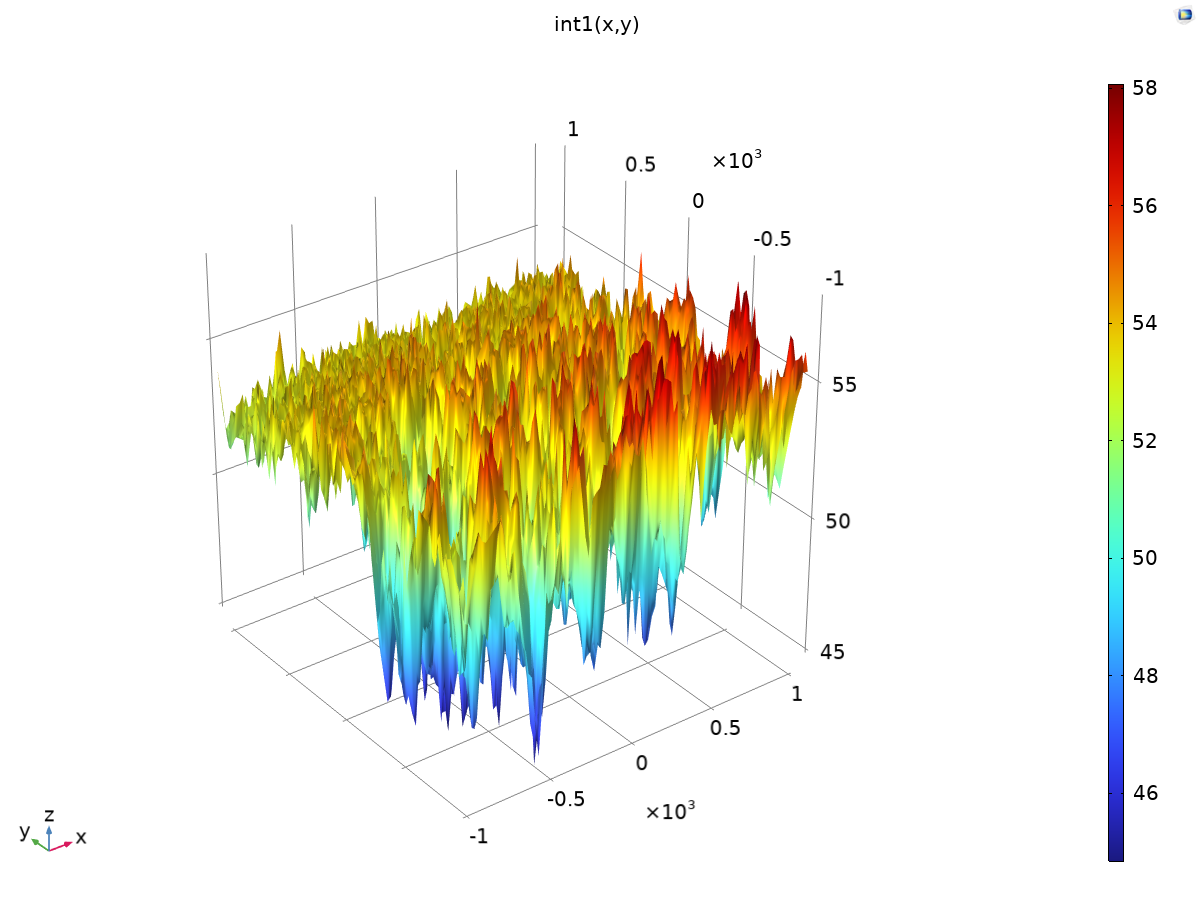 | 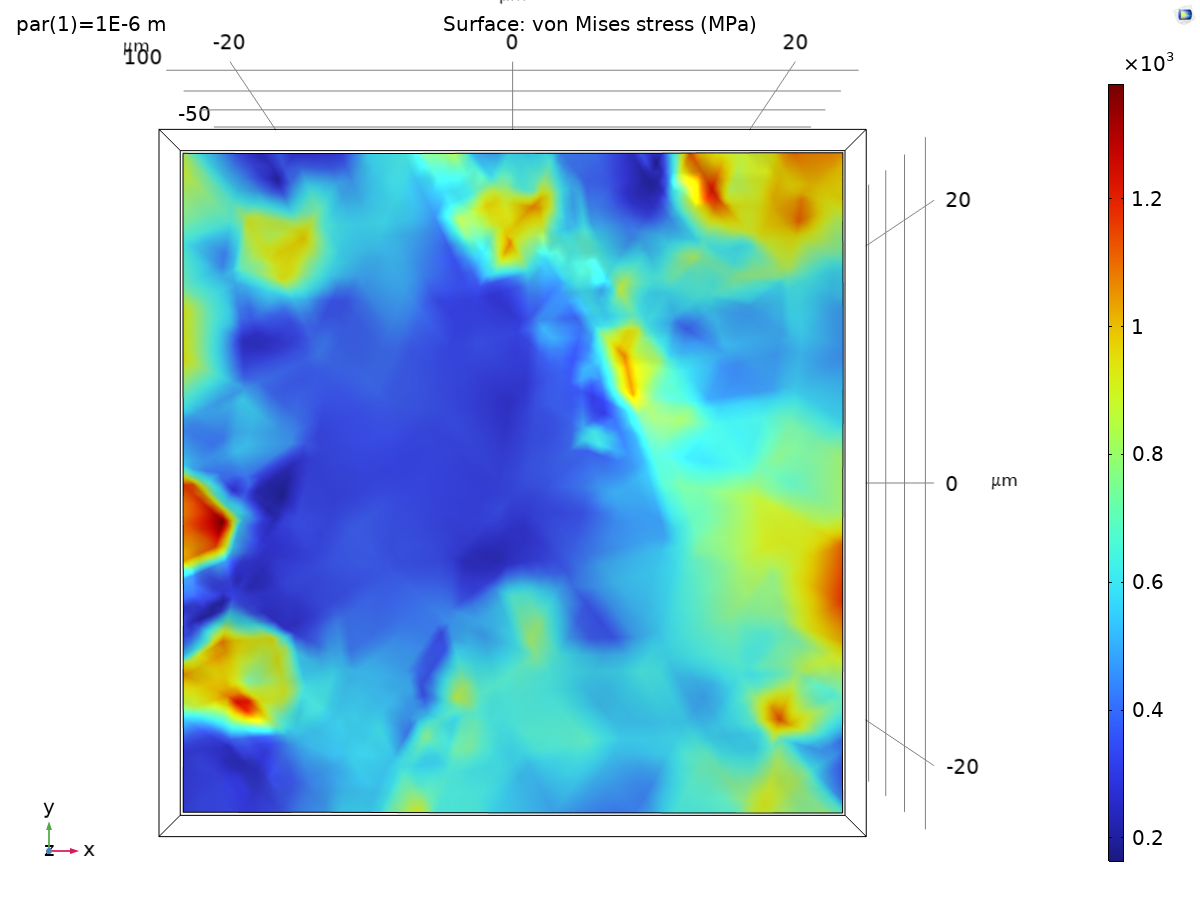 | 10hrs  200 MPa | 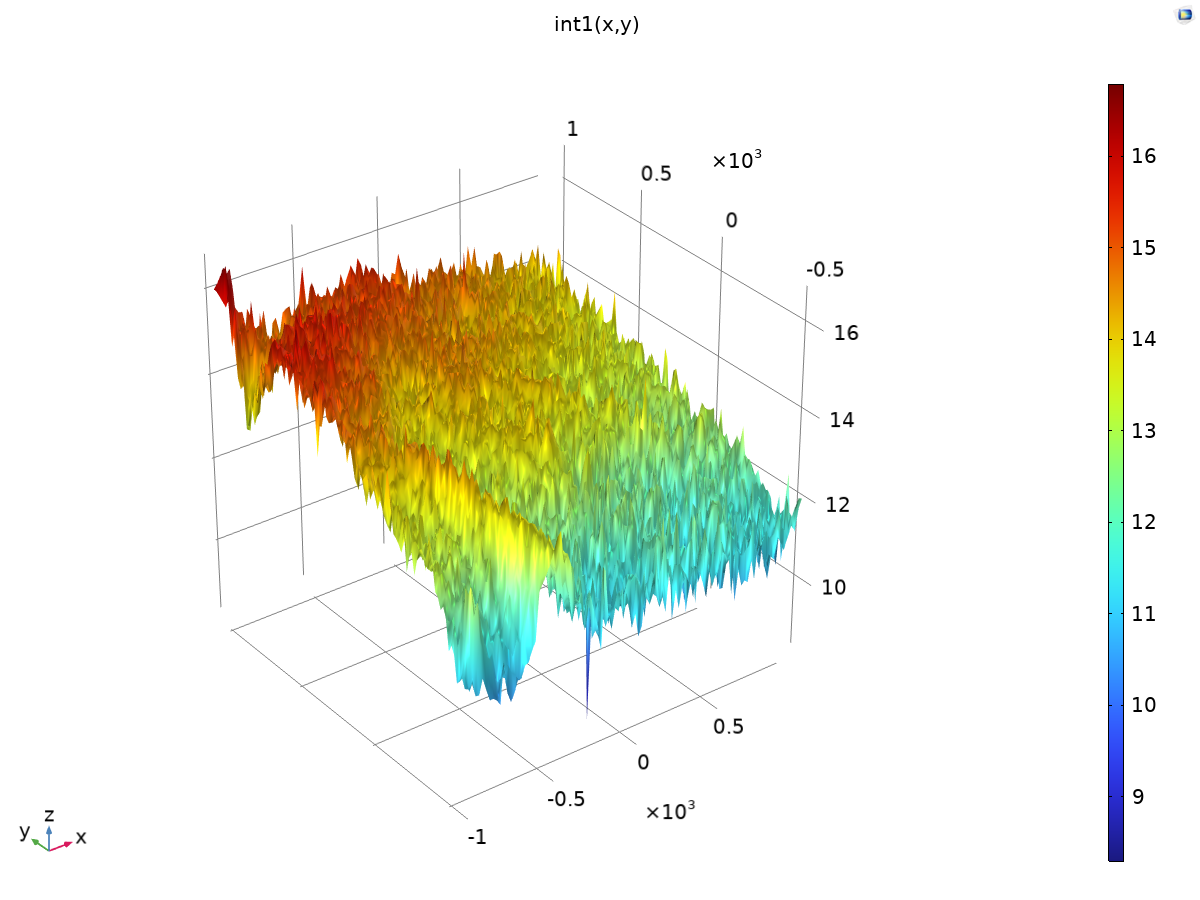 | 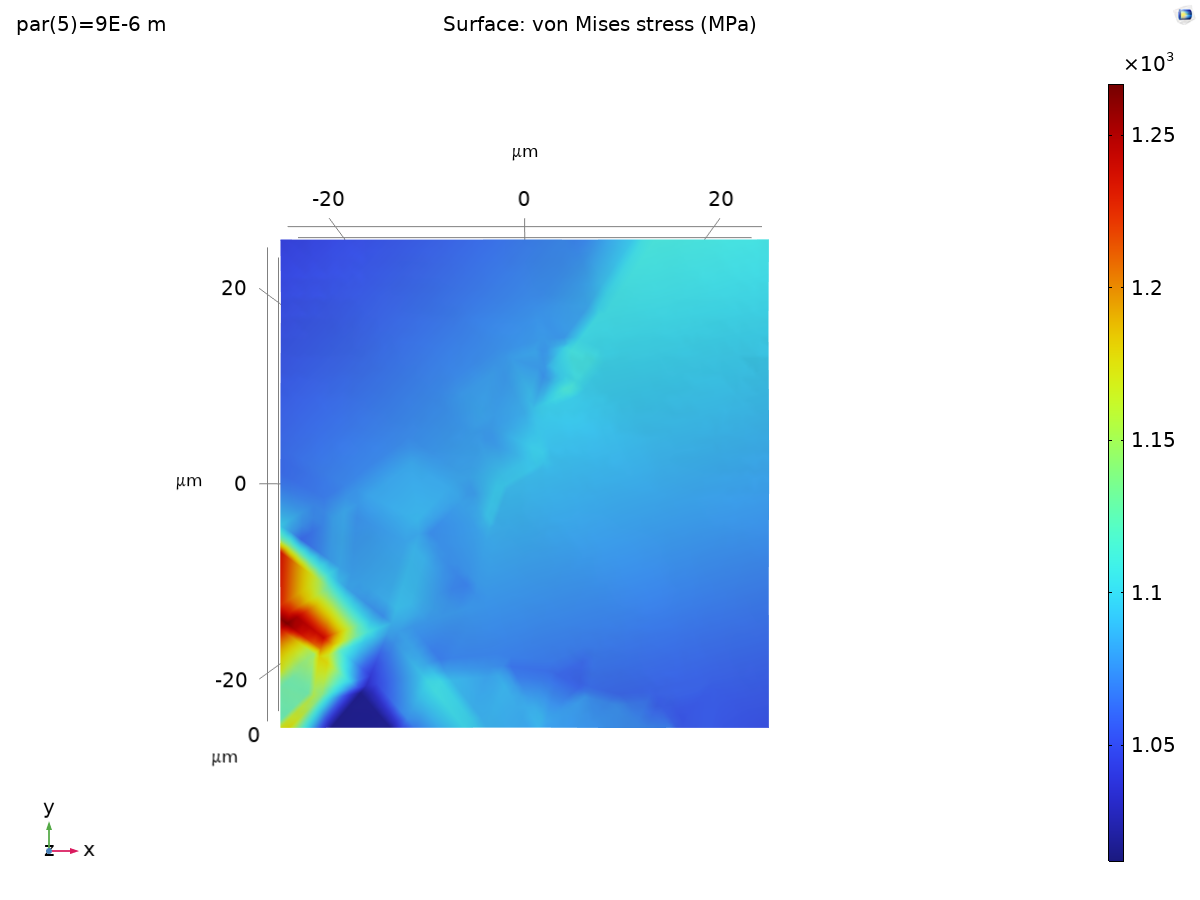 |
| 10hrs  400 MPa | 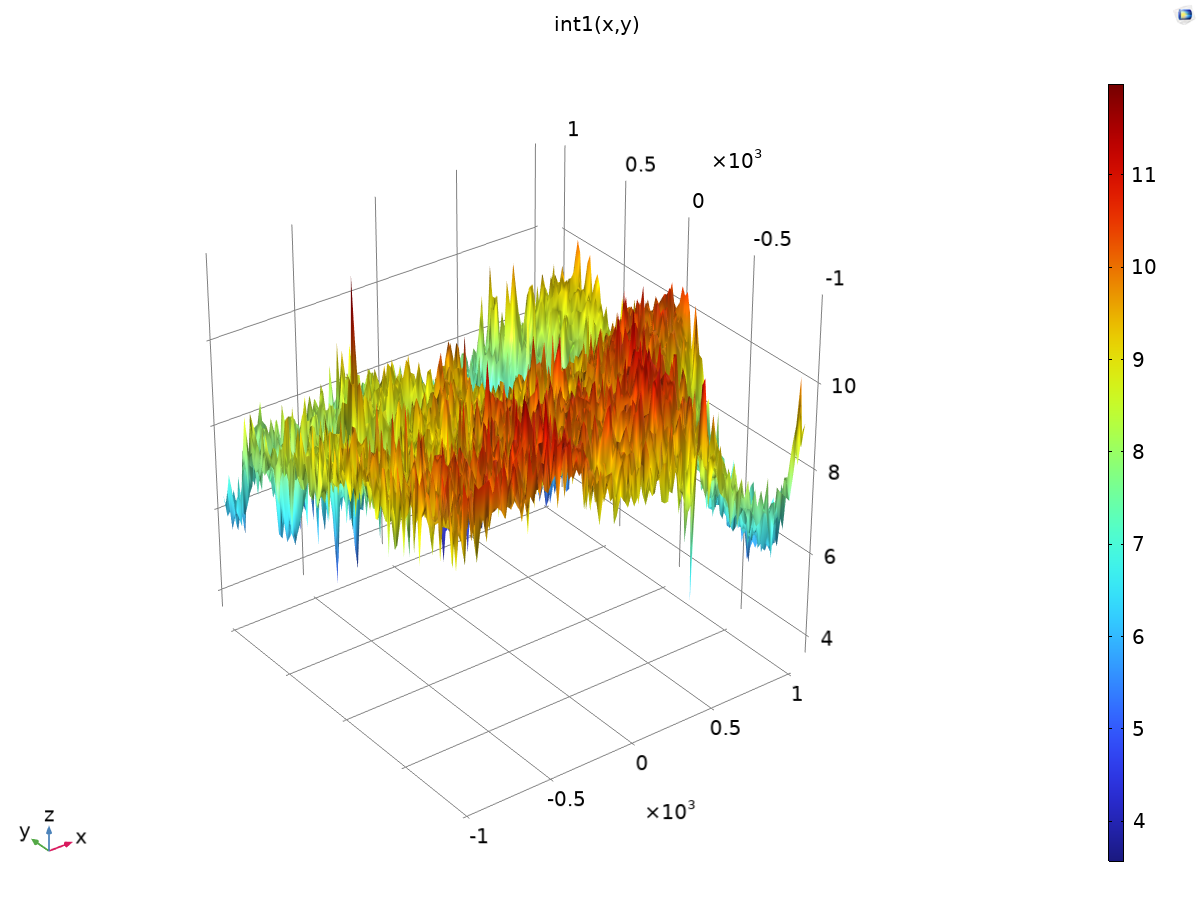 | 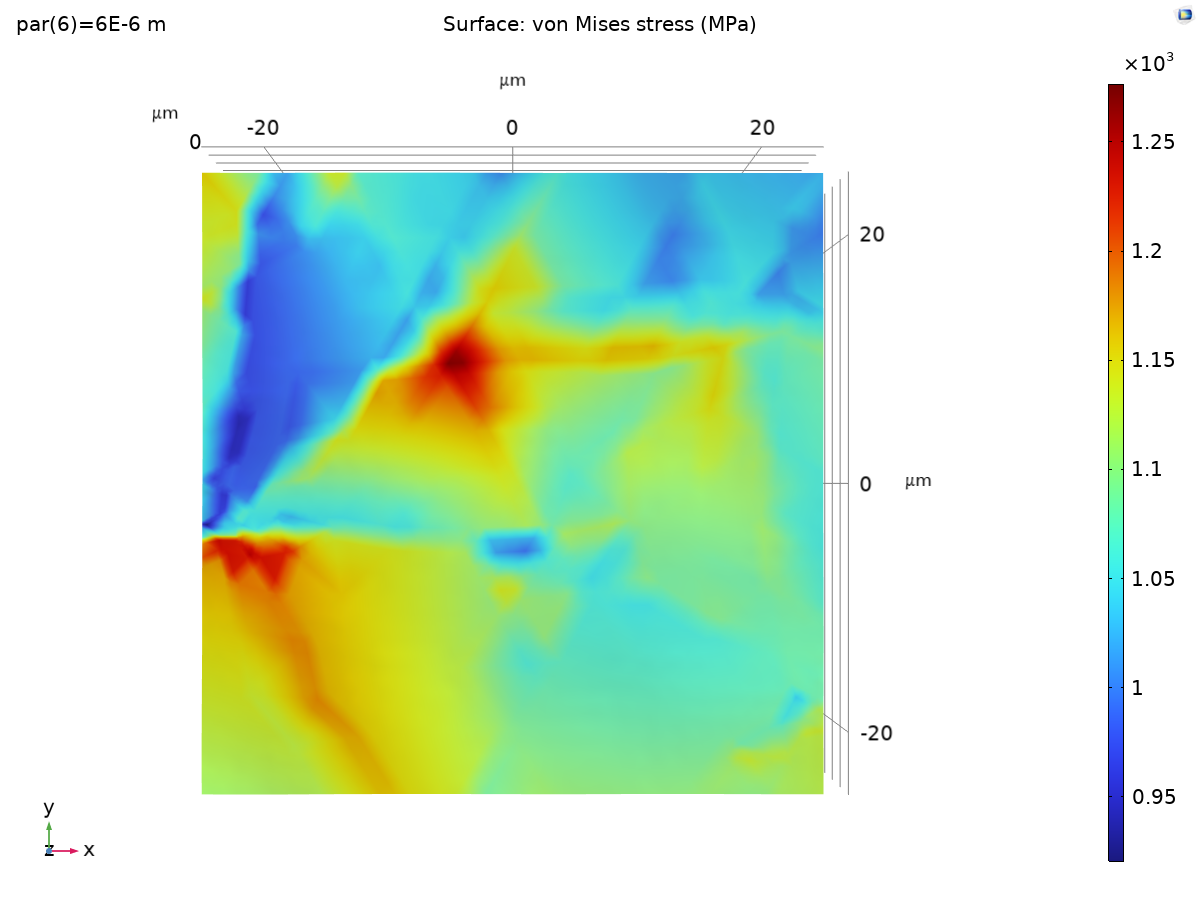 | 10hrs  600 MPa | 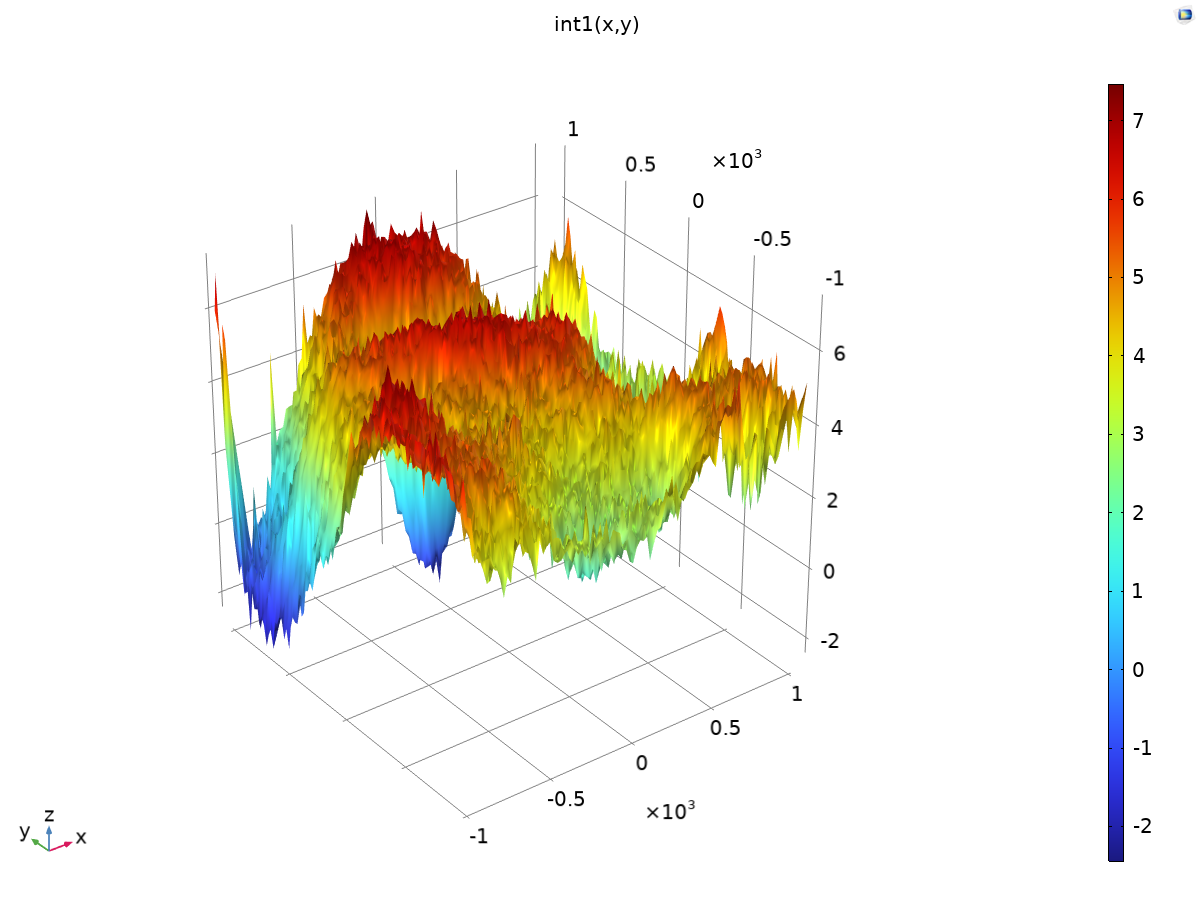 | 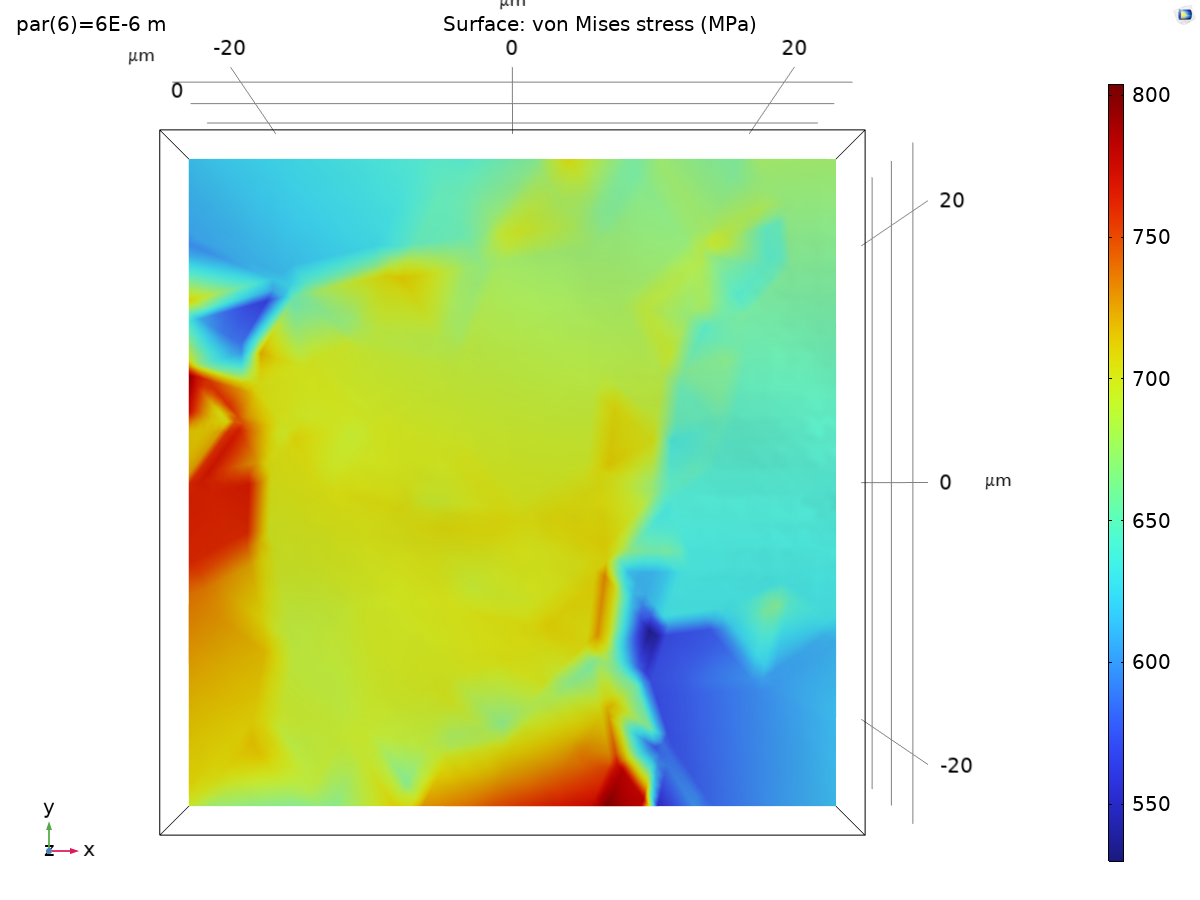 |

**Figure S22:** Variation of surface morphology and the corresponding stress distribution calculated by for different samples.

| 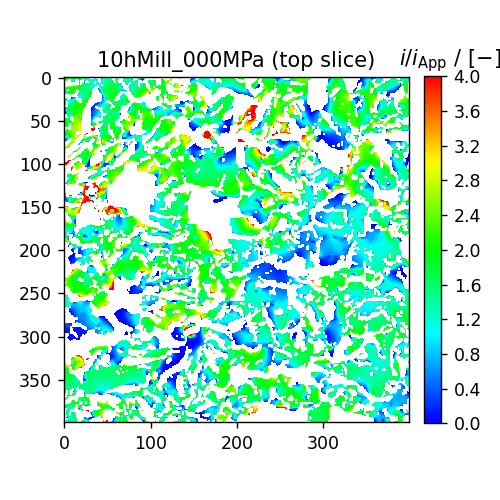**a** | 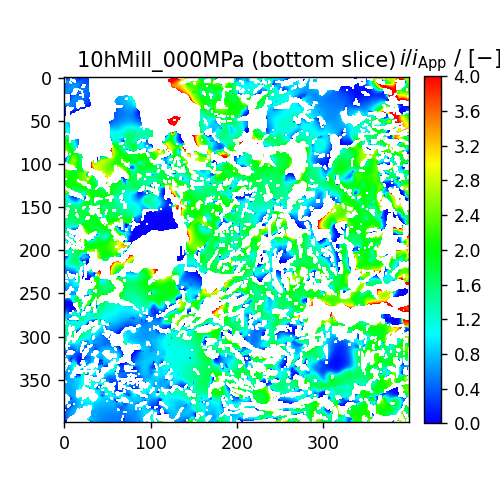**d** |
| --- | --- |
| 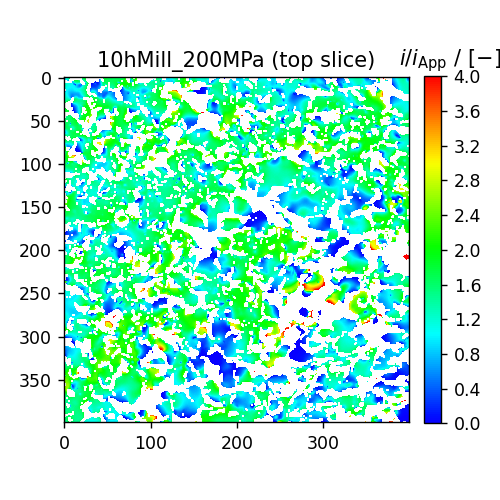**b** | 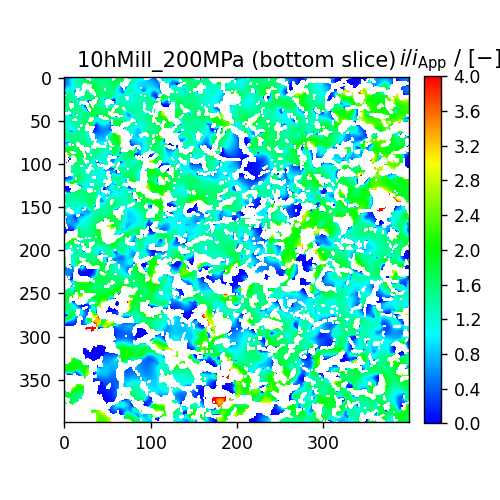**e** |
| 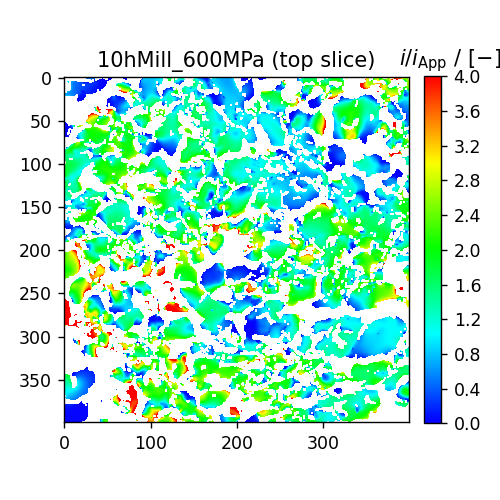**c** | 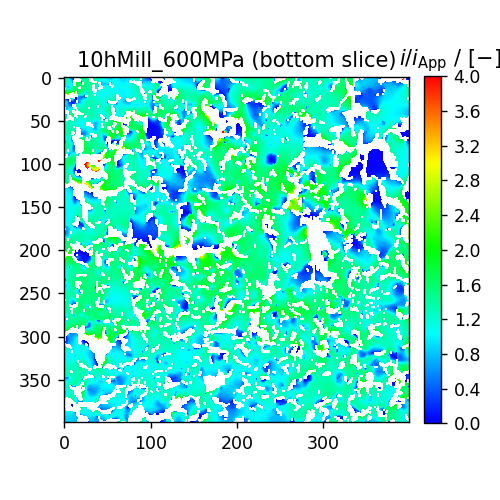**f** |

**Figure S23:** Normalized current density distribution obtained by TA simulations at 2D-electrode|LPSCl interface for 10 h milled samples (a,b,c) at top slice when samples are pressed at 0,200,600 MPa pressure (d,e,f) at bottom slice when samples are pressed at 0, 200, 600 MPa pressure. Detailed explanations are provided in Notes S4 and S5.

| 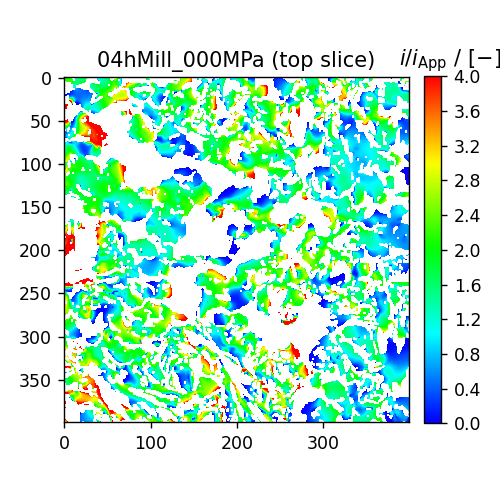**a** | 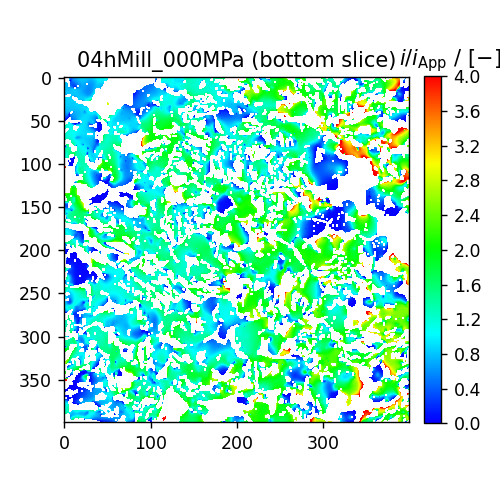**d** |
| --- | --- |
| 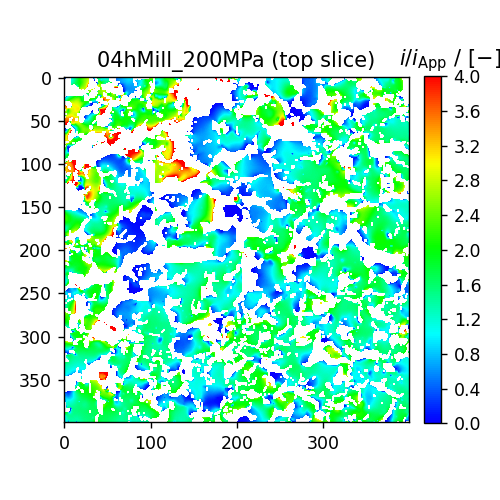**b** | 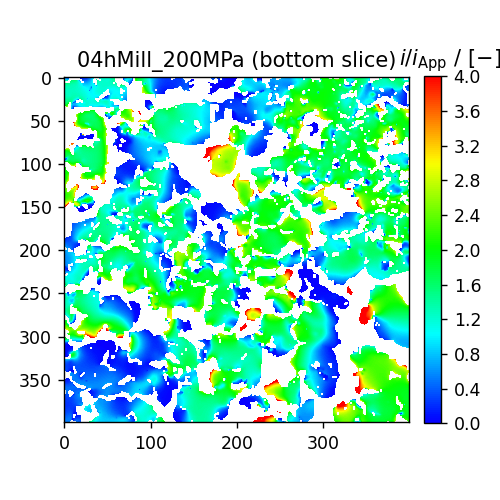**e** |
| 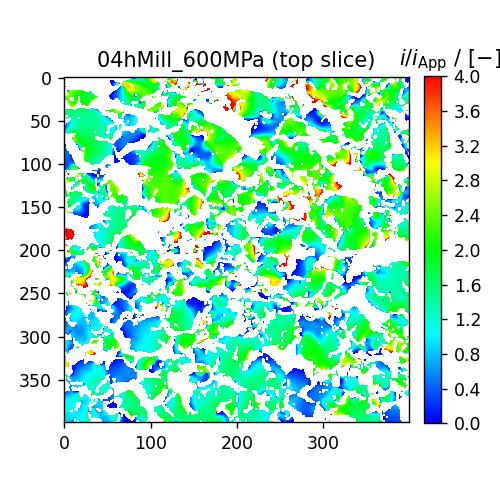**c** | 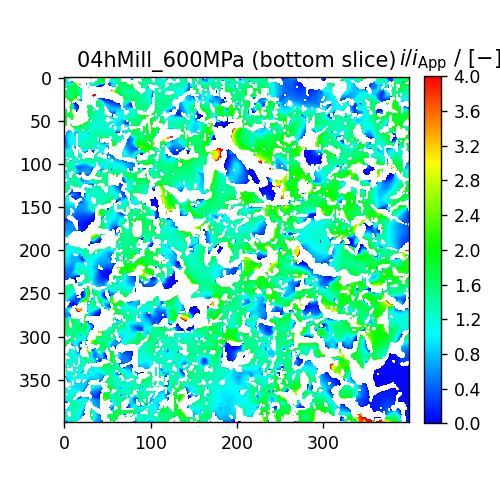**f** |

**Figure S24:** Normalized current density distribution obtained by TA simulations at 2D-electrode|LPSCl interface for 4 h milled samples (a,b,c) at top slice when samples are pressed at 0,200,600 MPa pressure (d,e,f) at bottom slice when samples are pressed at 0, 200, 600 MPa pressure. Detailed explanations are provided in Note S4 and S5.

| 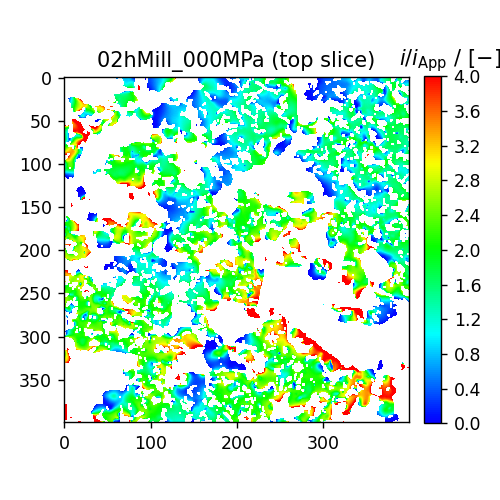**a** | 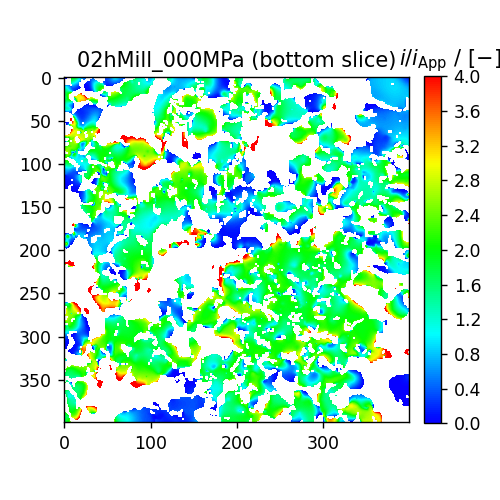**d** |
| --- | --- |
| 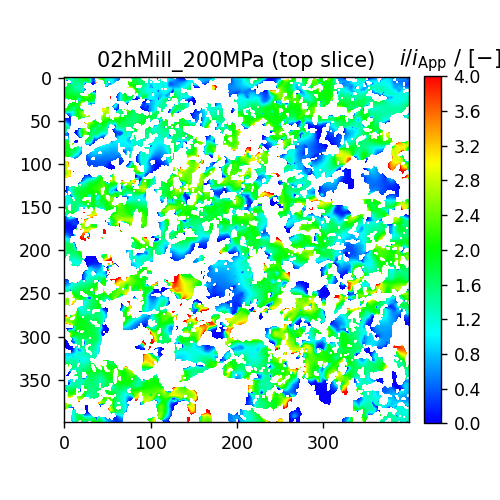**b** | 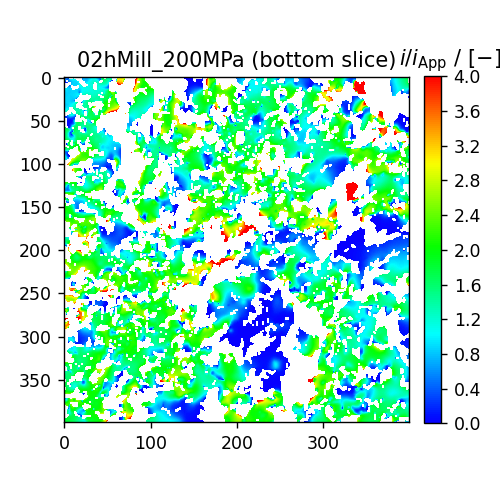**e** |
| 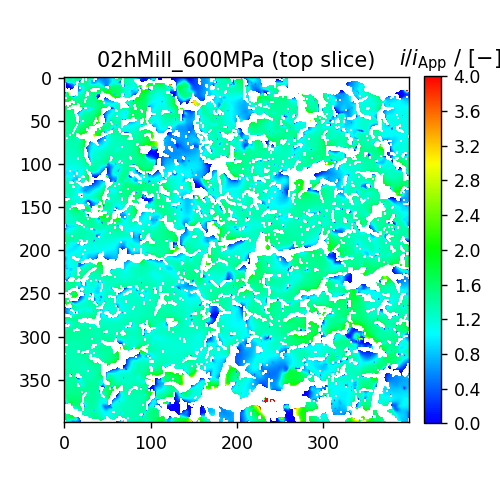**c** | 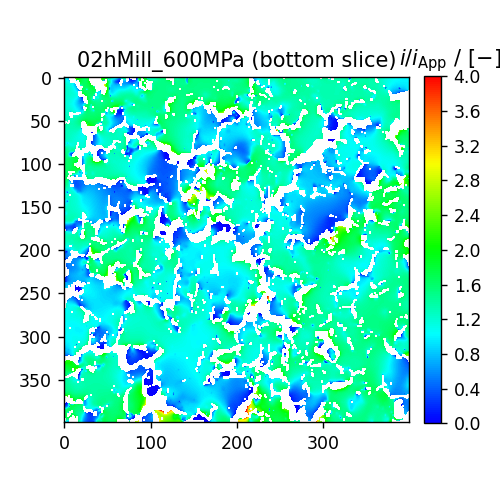**f** |

**Figure S25:** Normalized current density distribution obtained by TA simulations at 2D-electrode|LPSCl interface for 2 h milled samples (a,b,c) at top slice when samples are pressed at 0,200,600 MPa pressure (d,e,f) at bottom slice when samples are pressed at 0, 200, 600 MPa pressure. Detailed explanations are provided in Note S4 and S5.

| 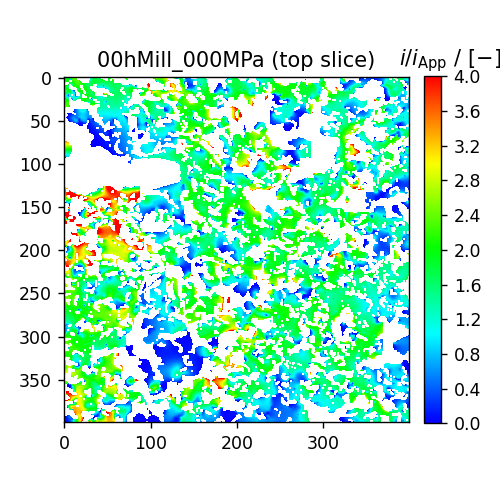**a** | 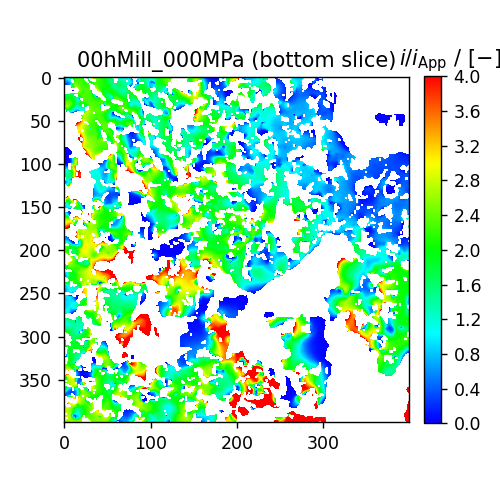**d** |
| --- | --- |
| 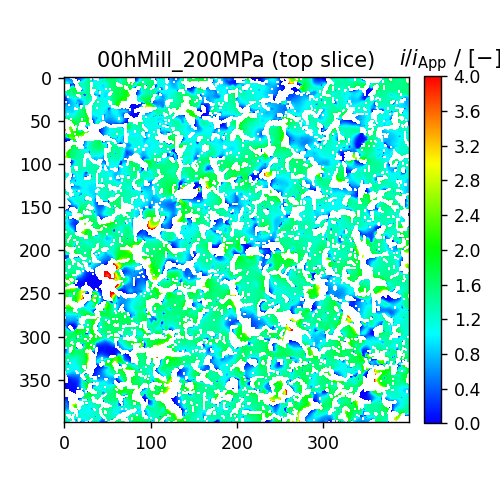**b** | 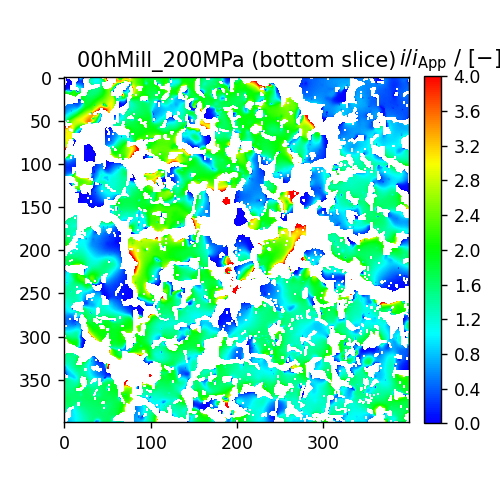**e** |
| 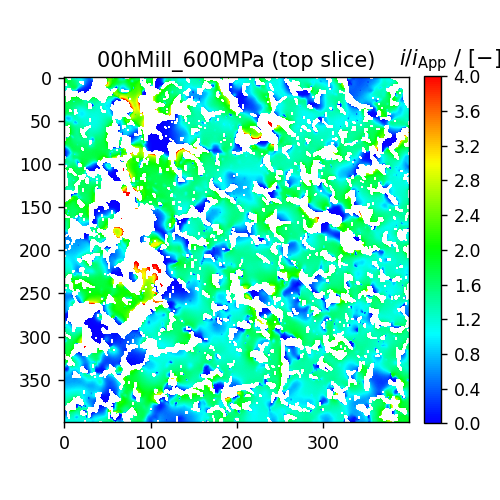**c** | 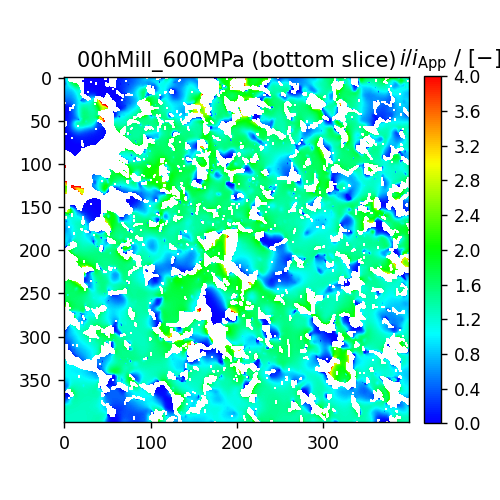**f** |

**Figure S26:** Normalized current density distribution obtained by TA simulations at 2D-electrode|LPSCl interface for non-milled (pristine) samples (a,b,c) at top slice when samples are pressed at 0,200,600 MPa pressure (d,e,f) at bottom slice when samples are pressed at 0, 200, 600 MPa pressure. Detailed explanations are provided in Note S4 and S5.

| 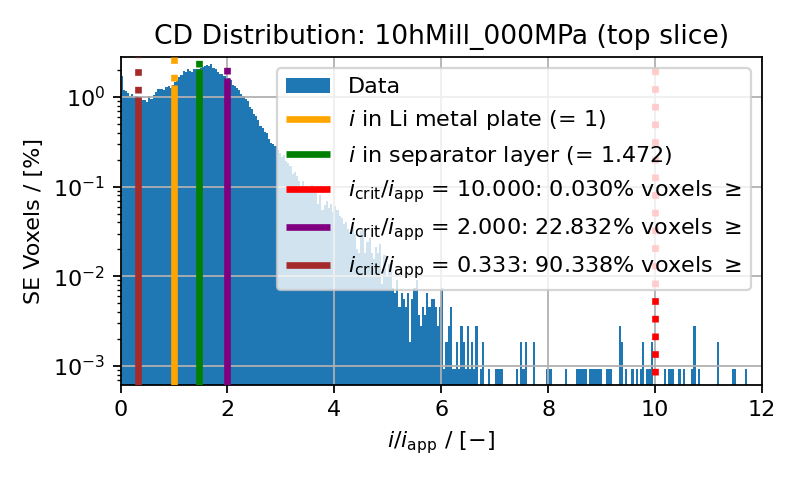 **A** | 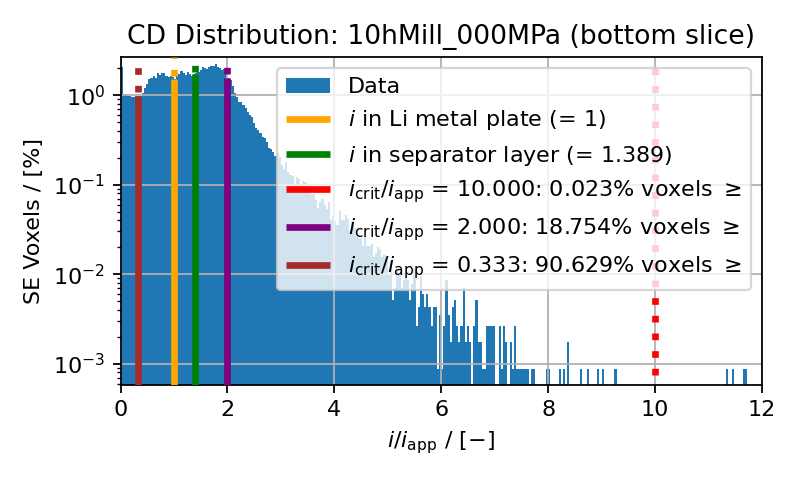 **d** |
| --- | --- |
| 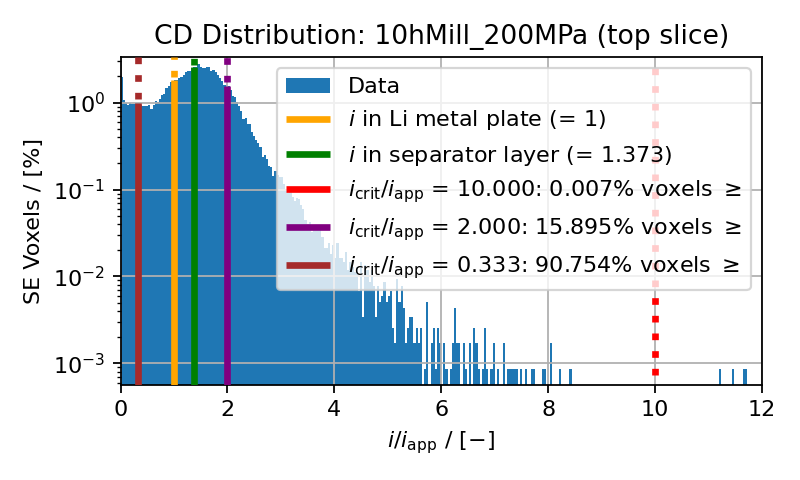 **B** | 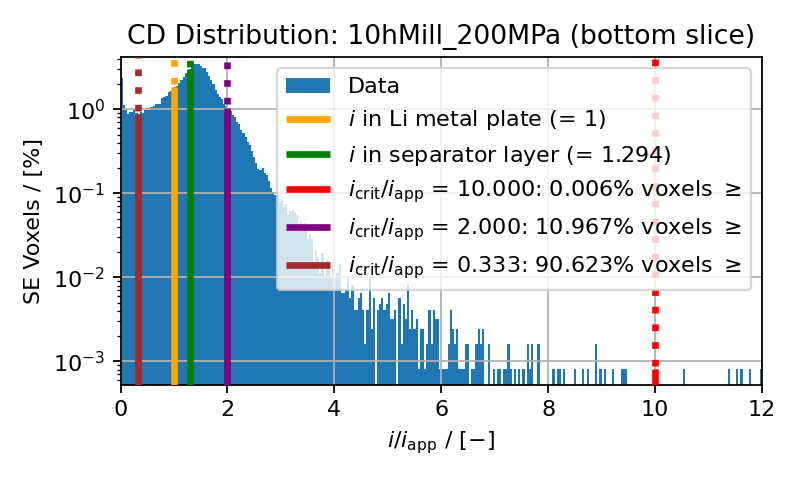**e** |
| 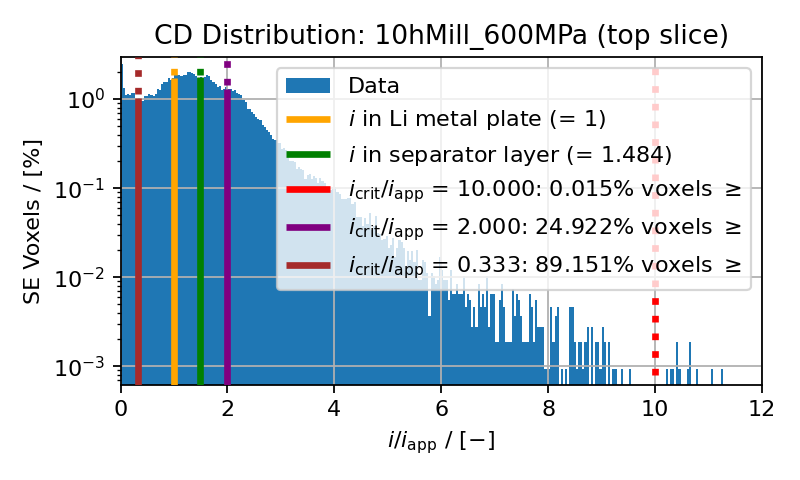 **c** | 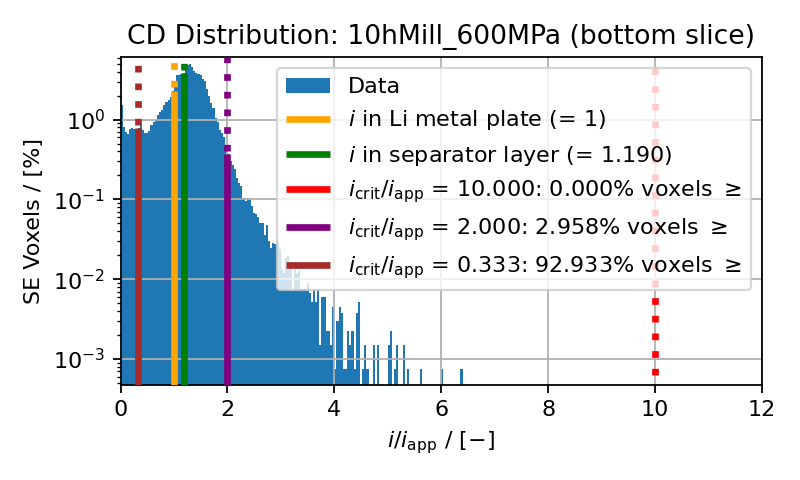 **f** |

**Figure S27:** Normalized current density distribution obtained by TA simulations at 2D-electrode|LPSCl interface for 10 h milled samples (a,b,c) at top slice when samples are pressed at 0,200,600 MPa pressure (d,e,f) at bottom slice when samples are pressed at 0, 200, 600 MPa pressure. The yellow line shows the normalized current density experienced by a lithium metal plate (= 1). Green line represents average current density experienced by the separator layer. This value is different for each sample due to the variations in surface layer porosity. The remaining red, purple and brownish red line show, where the CCD $i_{\mathrm{crit}}$ is 10x, 2x, and 0.333x the applied current density, respectively. This corresponds to the point where a critical current density i_crit_ = 1 mA/cm² is surpassed for applied current densities of 0.1, 0.5 and 3 mA/cm^2^, respectively. Detailed explanations are provided in Note S4 and S5.

| 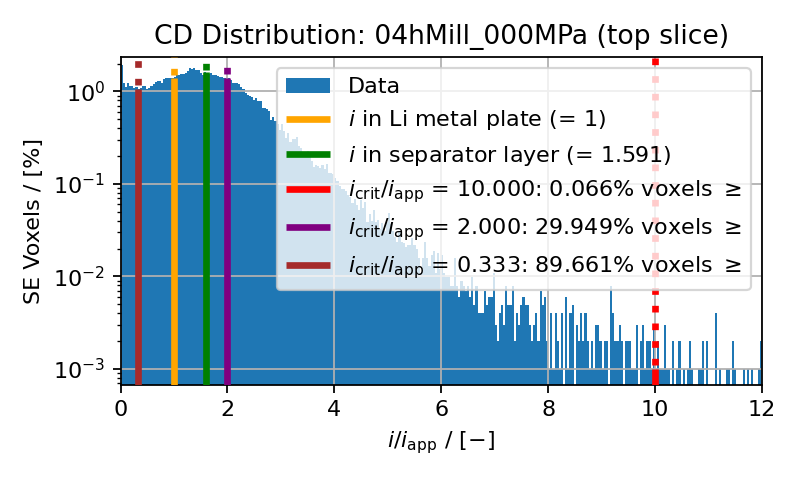 **A** | 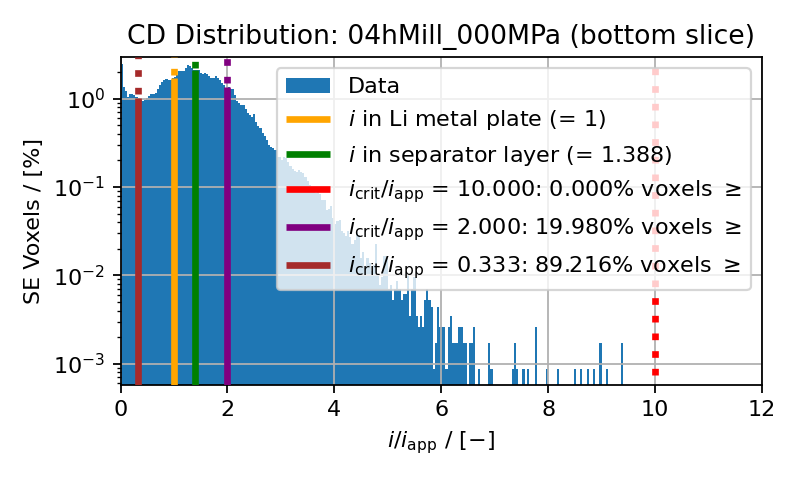 **d** |
| --- | --- |
| 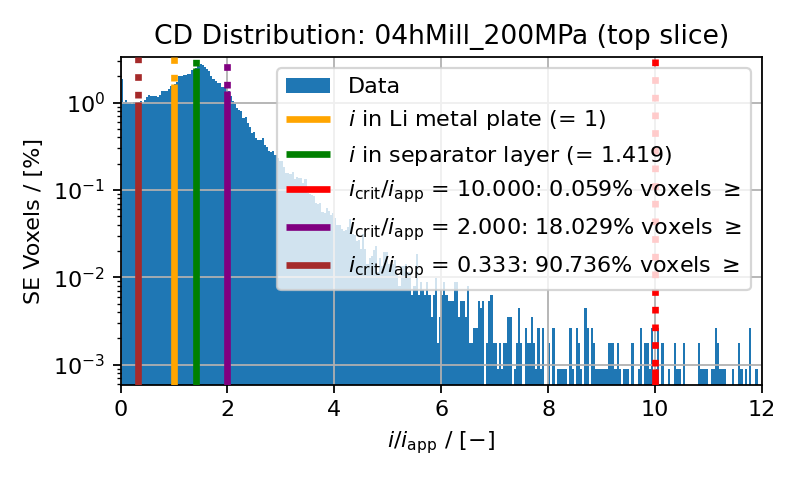 **B** | 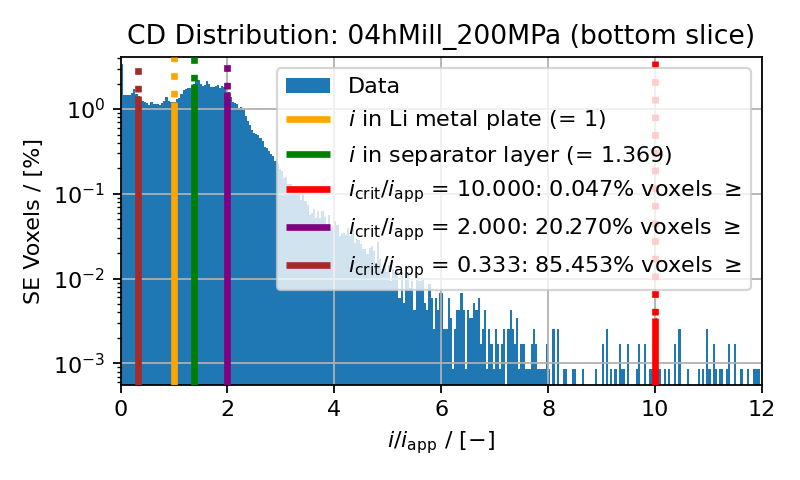**e** |
| 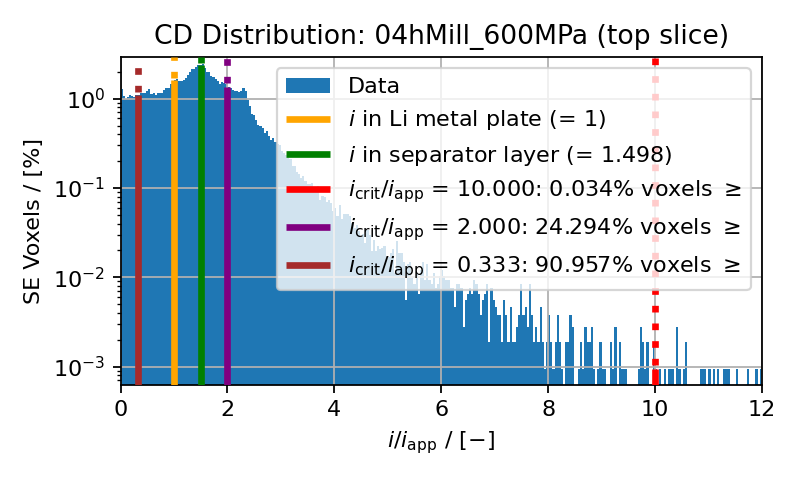 **c** | 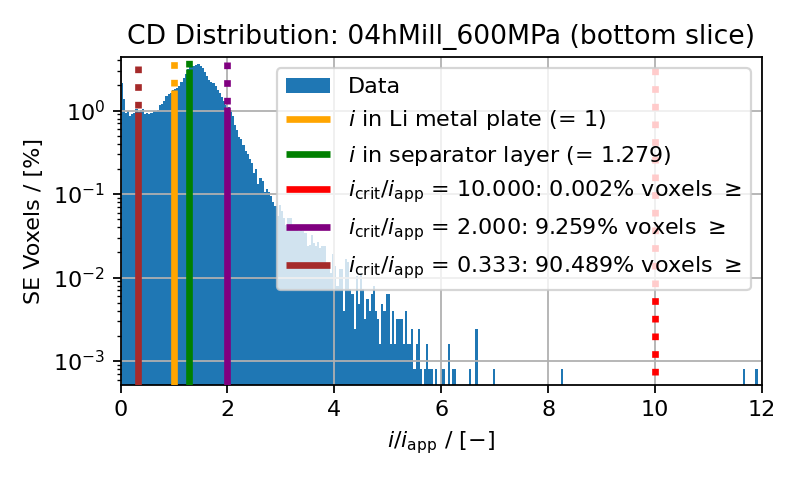 **f** |

**Figure S28:** Normalized current density distribution obtained by TA simulations at 2D-electrode|LPSCl interface for 4 h milled samples (a,b,c) at top slice when samples are pressed at 0,200,600 MPa pressure (d,e,f) at bottom slice when samples are pressed at 0, 200, 600 MPa pressure. The yellow line shows the normalized current density experienced by a lithium metal plate (= 1). Green line represents average current density experienced by the separator layer. This value is different for each sample due to the variations in surface layer porosity. The remaining red, purple and brownish red line shows, where the CCD $i_{\mathrm{crit}}$ is 10x , 2x, and 0.333x the applied current density, respectively. This corresponds to the point where a critical current density i_crit_ = 1 mA/cm² is surpassed for applied current densities of 0.1 , 0.5 and 3 mA/cm^2^, respectively. Detailed explanations are provided in Note S4 and S5.

| 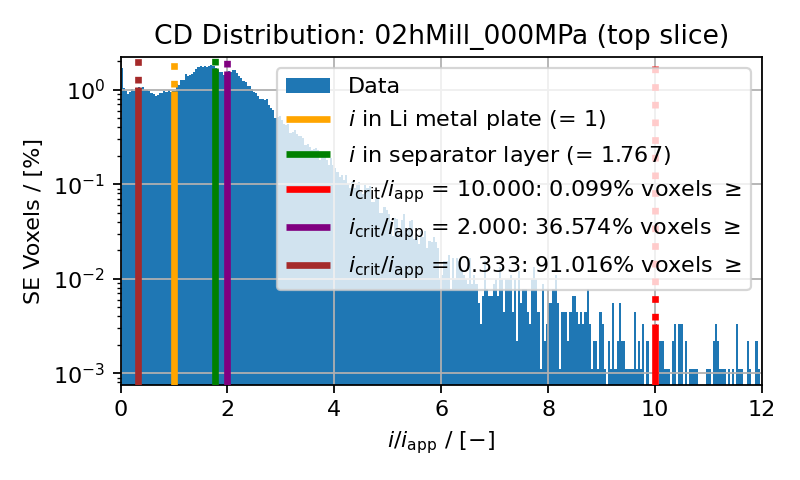 **A** | 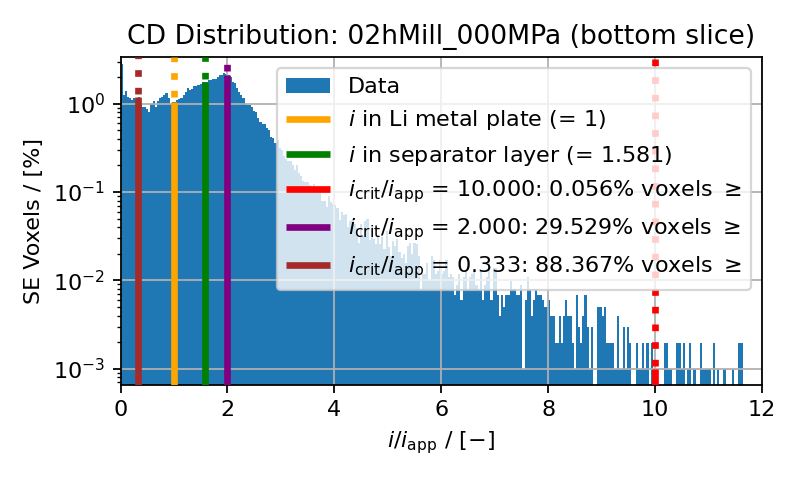 **d** |
| --- | --- |
| 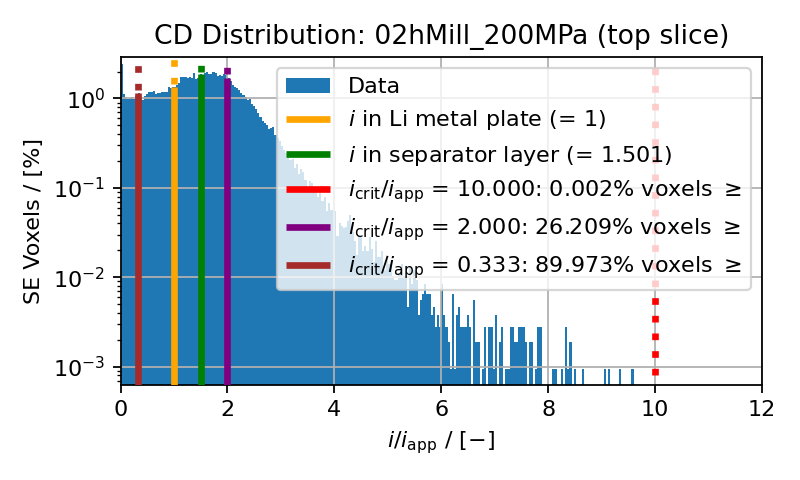 **B** | 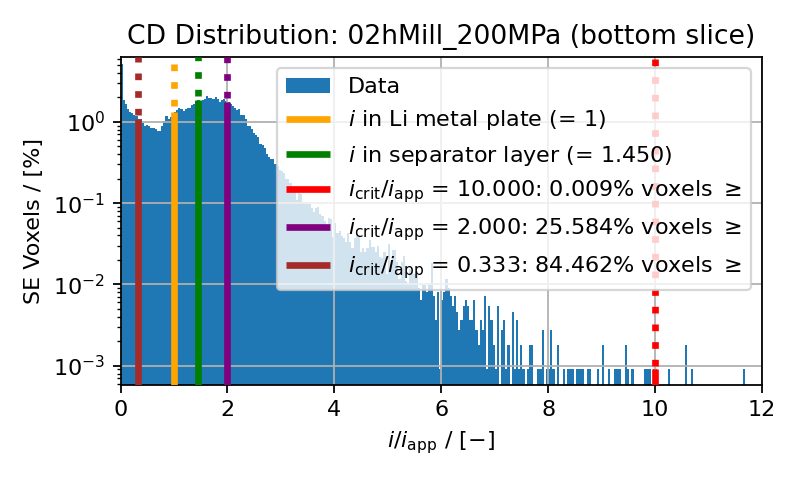**e** |
| 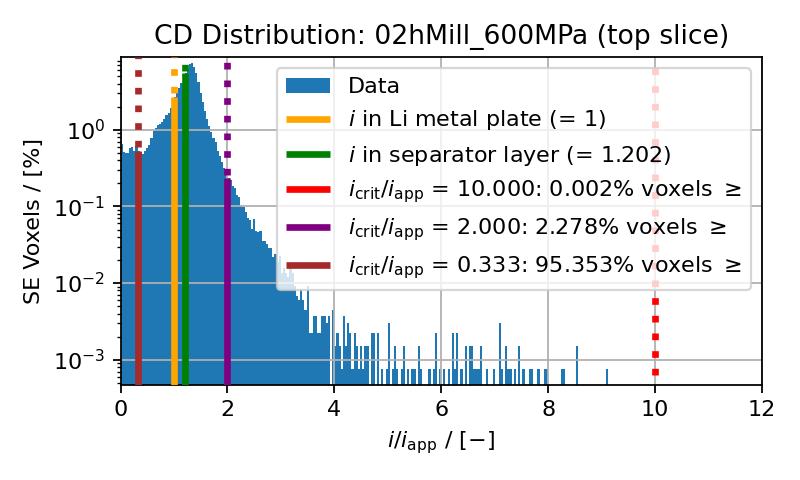 **c** | 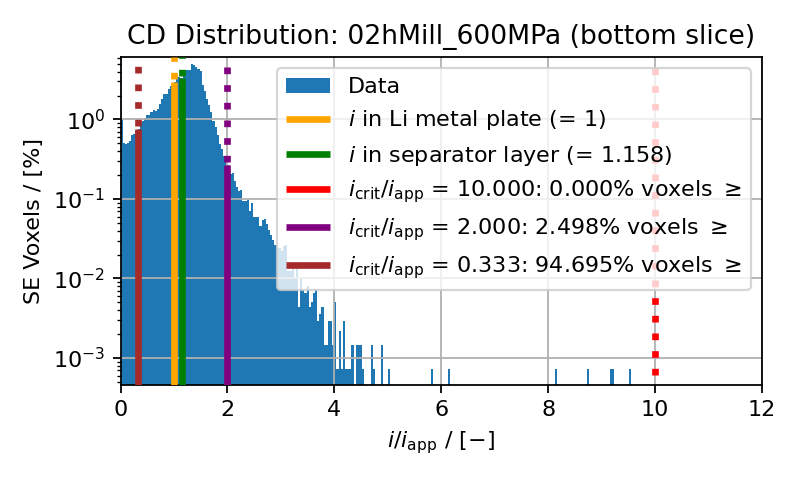 **f** |

**Figure S29:** Normalized current density distribution obtained by TA simulations at 2D-electrode|LPSCl interface for 2 h milled samples (a,b,c) at top slice when samples are pressed at 0,200,600 MPa pressure (d,e,f) at bottom slice when samples are pressed at 0, 200, 600 MPa pressure. The yellow line shows the normalized current density experienced by a lithium metal plate (= 1). Green line represents average current density experienced by the separator layer. This value is different for each sample due to the variations in surface layer porosity. The remaining red, purple and brownish red line shows, where the CCD $i_{\mathrm{crit}}$ is 10x , 2x, and 0.333x the applied current density, respectively. This corresponds to the point where a critical current density i_crit_ = 1 mA/cm² is surpassed for applied current densities of 0.1 , 0.5 and 3 mA/cm^2^, respectively. Detailed explanations are provided in Note S4 and S5.

| 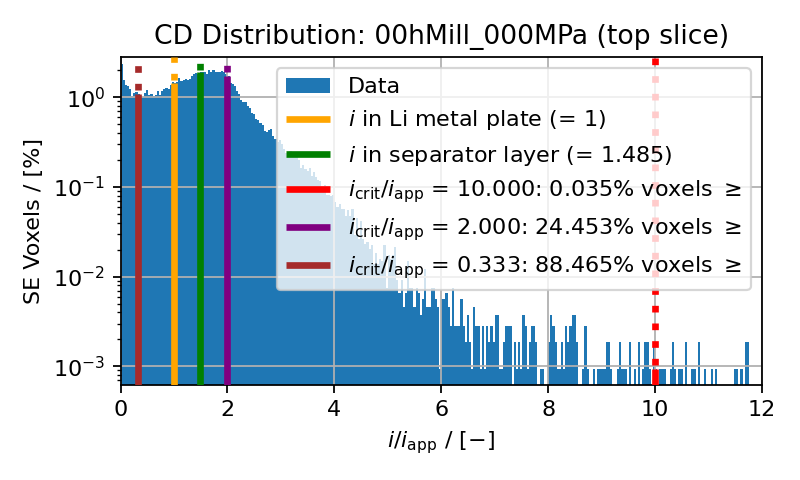 **A** | 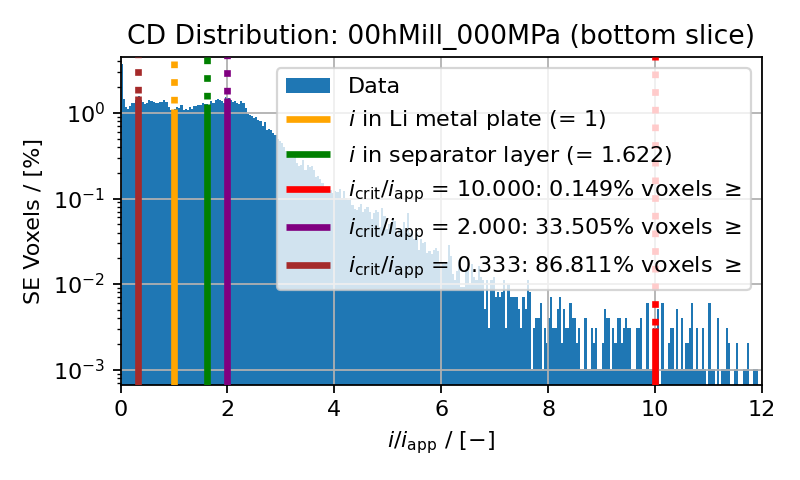 **d** |
| --- | --- |
| 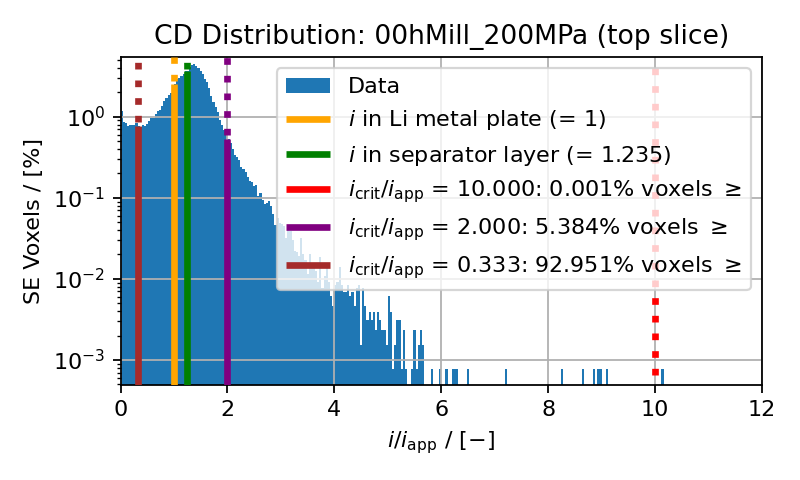 **B** | 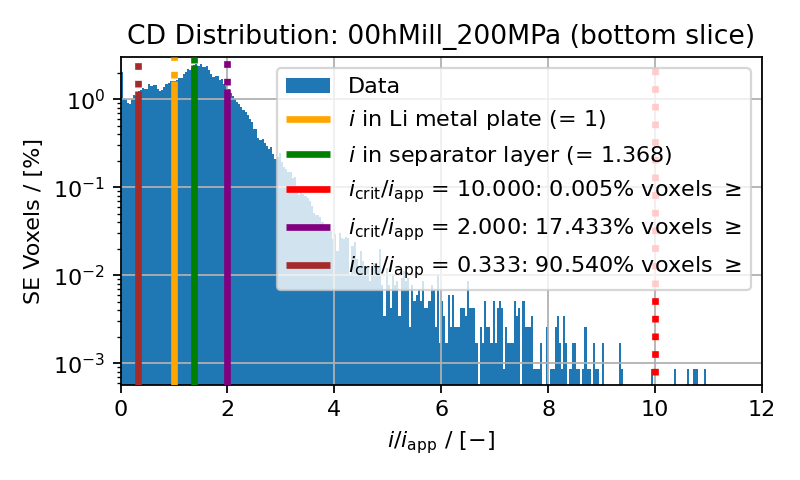**e** |
| 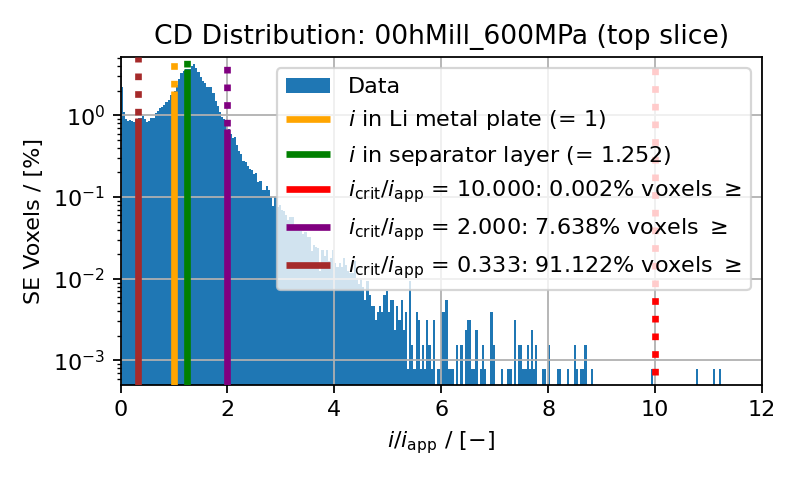 **c** | 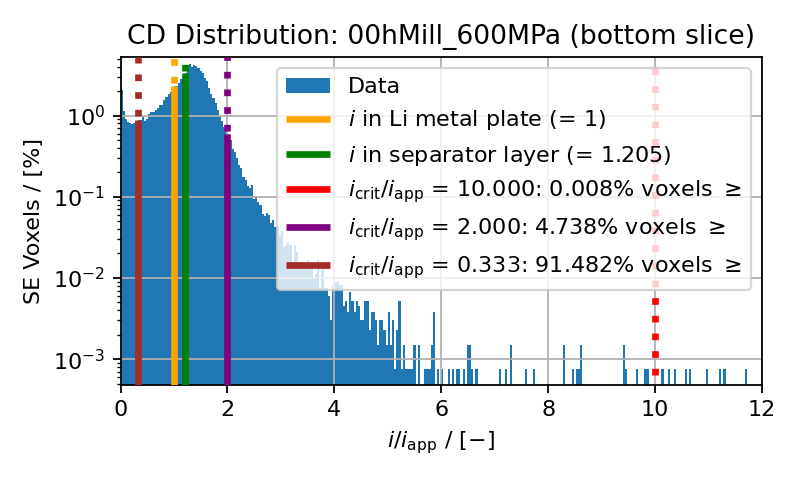 **f** |

**Figure S30:** Normalized current density distribution obtained by TA simulations at 2D-electrode|LPSCl interface for non-milled (pristine) samples (a,b,c) at top slice when samples are pressed at 0, 200, 600 MPa pressure (d, e, f) at bottom slice when samples are pressed at 0,200,600 MPa pressure. The yellow line shows the normalized current density experienced by a lithium metal plate (= 1). Green line represents average current density experienced by the separator layer. This value is different for each sample due to the variations in surface layer porosity. The remaining red, purple and brownish red line shows, where the CCD $i_{\mathrm{crit}}$ is 10x , 2x, and 0.333x the applied current density, respectively. This corresponds to the point where a critical current density i_crit_ = 1 mA/cm² is surpassed for applied current densities of 0.1 , 0.5 and 3 mA/cm^2^, respectively. Detailed explanations are provided in Note S4 and S5.

**Supplementary Tables**

**Table S1.** Type and amount of binder, densification pressure, porosity, mechanical flexibility, and Li-ion conductivities as reported in literature for LPSCl tapes.

| **No** | **Type** | **Amount** | **Li-ion conductivity**  **S/cm** | **Densification pressure**  **MPa** | **Porosity**  **%** | **Mechanical flexibility** | **Ref.** |
| --- | --- | --- | --- | --- | --- | --- | --- |
| 1 | PBMA | 3%−10% | 1.5·10^−4^ - 8.6·10^−4^ | 370 | - | - | Chen et al.^[4]^ |
| **2** | acrylate type | 1 wt % | 1.3·10^−3^ | 300 | - | sufficient | Lee et al.^[5]^ |
| **3** | HNBR | 5 wt% | 0.5·10^−3^ | 70 | 24 | - | Sedlmeier et al.^[6]^ |
| **4** | HNBR | 5 wt% | 0.94·10^−3^ | 590 | 3 | - | Sedlmeier et al.^[6]^ |
| **5** | HNBR | 5 vol% | 5.80·10^-4^ | 300 | 45 | High | Tron et al.^[7]^ |
| **6** | PIB | 5 vol% | 1.24·10^-3^ | 300 | 43 | High | Tron et al.^[7]^ |
| **7** | NBR | 5 vol% | 5.84·10^-4^ | 300 | 31 | High | Tron et al.^[7]^ |
| **8** | PBMA | 5 vol% | 1.45·10^-3^ | 300 | 6 | Very low | Tron et al.^[7]^ |
| **9** | SBS | 5 vol% | 9.97·10^-4^ | 300 | 20 | Medium | Tron et al.^[7]^ |
| **10** | SEBS | 5 vol% | 1.19·10^-3^ | 300 | 42 | Medium | Tron et al.^[7]^ |
| **11** | PIB | 5 wt% | ~3.8·10^-5^ - 9 ·10^-5^ | 0 - 20 | ~ 14 | High | Mills et al.^[8]^ |

**Table S2.** Total Transferred Energy for HDM with BPR values of 10:1, 20:1, and 30:1 for a milling time of 10 hours.

| BPR (Ball-to-Powder Ratio) | Total Energy Transferred (J) |
| --- | --- |
| 10:1 | 3.82·10^13^ |
| 20:1 | 3.05·10^14^ |
| 30:1 | 1.03·10^15^ |

**Table S3.** Total Transferred Energy for LWM for a fixed BPR of 30:1 across milling times of 2, 4, and 10 hours.

| Milling Time (h) | Total Energy Transferred (J) |
| --- | --- |
| 2 | 5.99·10^12^ |
| 4 | 1.20·10^13^ |
| 10 | 2.99·10^13^ |

**Table S4** Quantification of amorphous content in LPSCl after different milling treatments using Rietveld refinement with crystalline silicon as an internal standard.

| Samples | Amorphous content (%) |
| --- | --- |
| Pristine LPSCl | 0.2 |
| LWM 2 hours | 2.7 |
| LWM 4 hours | 5.1 |
| LWM 10 hours | 8.6 |
| HDM 10:1 | 25.5 |
| HDM 20:1 | 29.7 |
| HDM 30:1 | 29.2 |

**Table S5.** Summary of Li-Ion conductivity (mS/cm) values measured via EIS using Compredrive under varying pressure conditions. Here, for 10h milling, initial pressure for measuring conductivity is 50 MPa instead of 10 MPa. It might be because of smaller particle size; higher pressure is required to maintain contact between tape and plunger.

| Pressure (MPa) | Pristine | 2h | 4h | 10h |
| --- | --- | --- | --- | --- |
| 10 | 0.00143 | 0.0051 | 0.06026 | 0.23305 |
| 100 | 0.3327 | 0.39375 | 0.44431 | 0.62146 |
| 200 | 0.65319 | 0.66016 | 0.73286 | 0.80363 |
| 300 | 0.80005 | 0.81779 | 0.91623 | 0.90363 |
| 400 | 0.88846 | 0.91799 | 1.02174 | 0.92929 |
| 500 | 0.93345 | 0.96924 | 1.0638 | 0.91319 |
| 600 | 0.93487 | 1.01419 | 1.09802 | 0.89475 |
| 700 | 0.91612 | 1.03248 | 1.10907 | 0.8714 |
| 800 | 0.8672 | 1.03469 | 1.0987 | 0.8509 |
| 900 | 0.8672 | 1.02426 | 1.09413 | 0.8248 |
| 1000 | 0.8672 | 1.02518 | 1.073 | 0.80362 |

**Table S6.** The current ratios calculated by the EA model for different samples

| **i_app=0.5** | | | | | |
| --- | --- | --- | --- | --- | --- |
| *Name* | *Milling time  (hr)* | *Pressure  (MPa)* | *Av. Max/Min current ratio (mA/cm^2^)* | *Positive variation among different realizations* | *Negative variation among different realizations* |
| *Pristine 0MPa* | 0 | 0 | 1.040181799 | 0.005638292 | 0.008917161 |
| *Pristine 200MPa* | 0 | 200 | 1.053629437 | 0.007836309 | 0.008289654 |
| *Pristine 400MPa* | 0 | 400 | 1.043937892 | 0.027019531 | 0.013238527 |
| *2h 0MPa* | 2 | 0 | 1.061231624 | 0.021216945 | 0.015049405 |
| *2h 200MPa* | 2 | 200 | 1.053629437 | 0.007836309 | 0.008289654 |
| *2h 400MPa* | 2 | 400 | 1.033925999 | 0.013504571 | 0.007101517 |
| *4h 0MPa* | 4 | 0 | 1.064350584 | 0.013240352 | 0.012051523 |
| *4h 200MPa* | 4 | 200 | 1.060364754 | 0.008165157 | 0.005102872 |
| *4h 400MPa* | 4 | 400 | 1.058277195 | 0.02436034 | 0.022453586 |
| *10h 0MPa* | 10 | 0 | 1.064532144 | 0.008723673 | 0.013634712 |
| *10h 200MPa* | 10 | 200 | 1.047768366 | 0.014591565 | 0.012315137 |
| *10h 400MPa* | 10 | 400 | 1.039204762 | 0.009795016 | 0.010234594 |
| **i_app=3** | | | | | |
| *Name* | *Milling time  (hr)* | *Pressure  (MPa)* | *Av. Max/Min current ratio (mA/cm^2^)* | *Positive variation among different realizations* | *Negative variation among different realizations* |
| *Pristine 0MPa* | 0 | 0 | 1.057986579 | 0.006746362 | 0.013842857 |
| *Pristine 200MPa* | 0 | 200 | 1.073531362 | 0.007319345 | 0.0106152 |
| *Pristine 400MPa* | 0 | 400 | 1.065393361 | 0.041313864 | 0.021245832 |
| *2h 0MPa* | 2 | 0 | 1.084376208 | 0.026174339 | 0.02109207 |
| *2h 200MPa* | 2 | 200 | 1.073531362 | 0.007319345 | 0.0106152 |
| *2h 400MPa* | 2 | 400 | 1.051816697 | 0.019379958 | 0.009933481 |
| *4h 0MPa* | 4 | 0 | 1.086194275 | 0.022003502 | 0.02634926 |
| *4h 200MPa* | 4 | 200 | 1.084119838 | 0.012986003 | 0.008606863 |
| *4h 400MPa* | 4 | 400 | 1.07802314 | 0.031889734 | 0.031367184 |
| *10h 0MPa* | 10 | 0 | 1.0877602 | 0.013880672 | 0.019075658 |
| *10h 200MPa* | 10 | 200 | 1.067343453 | 0.022075177 | 0.017800516 |
| *10h 400MPa* | 10 | 400 | 1.057268676 | 0.011386969 | 0.013426466 |

**Table S7.** The value of the parameters used to solve the electrochemical-transport model

| **Parameter** | **Value** | **Unit** |
| --- | --- | --- |
| $k_{SE}$ | 3E-4 | S/cm |
| $i_{0}$ | 7.82 | A/m^2 |
| $T$ | 298.15 | K |
| $i_{app}$ | -0.5 and -3 | mA/cm^2^ |

**Table S8.** The boundary conditions utilized to solve the stress model.

| Electrolyte | Pressing Block |
| --- | --- |
| - *Base face*: Fixed; no displacement in any direction ($u=0$). - *Side faces*: No displacement in the direction normal to the plane ($u.n=0$). | - The pressing block is displaced toward the electrolyte by a predefined displacement, applying stress on the electrolyte. |

**Table S9.** The mechanical properties of the electrolyte and the pressing block.

|  | **Electrolyte** | **Pressing Material** |
| --- | --- | --- |
| **Young’s Modulus** [GPa] | 22 | 130 |
| **Poisson Ratio** | 0.28 | 0.3 |
| **Density** [Kg/m^3^] | 1640 | 2330 |

**Table S10.** List of all effective conductivities and tortuosities obtained by the simulated conductivity measurements on the FIB-tomographies. The process and equations are detailed in Section 4d) of the main manuscript and Note S4.

| **Data** | **Solid Volume Fraction (-)** | **Local electrical conductivity / (S/cm)** | **Effective conductivity** $\boldsymbol{k}_{\mathbf{SE}}$ **(S/cm)** | **Tortuositiy** $\boldsymbol{\tau}_{\mathbf{SE}}$ **(-)** |
| --- | --- | --- | --- | --- |
| 00hMill_000MPa | 0.603275556 | 0.003076 | 0.000878424 | 2.112505588 |
| 00hMill_200MPa | 0.787829861 | 0.003076 | 0.00159671 | 1.517723727 |
| 00hMill_600MPa | 0.834165712 | 0.003076 | 0.00178526 | 1.437266129 |
| 02hMill_000MPa | 0.672007248 | 0.00214 | 0.000818459 | 1.757077033 |
| 02hMill_200MPa | 0.696267448 | 0.00214 | 0.000845008 | 1.763311517 |
| 02hMill_600MPa | 0.863814366 | 0.00214 | 0.00150819 | 1.225682934 |
| 04hMill_000MPa | 0.667959245 | 0.001741 | 0.000626444 | 1.856378296 |
| 04hMill_200MPa | 0.725396458 | 0.001741 | 0.000788324 | 1.602025606 |
| 04hMill_600MPa | 0.751675851 | 0.001741 | 0.000821219 | 1.593567192 |
| 10hMill_000MPa | 0.680313377 | 0.00127 | 0.000483198 | 1.788082708 |
| 10hMill_200MPa | 0.762647274 | 0.00127 | 0.000628357 | 1.541419986 |
| 10hMill_600MPa | 0.757281042 | 0.00127 | 0.000629571 | 1.527622656 |

**Table S11.** Evaluation of normalized current densities obtained by the simulated conductivity measurements on the FIB-tomographies Data from the top interface layer of the tomographies is shown. The process and equations including an explanation of $j_{>3\sigma}$ are detailed in Section 4d) of the main manuscript and Note S4.

| **Data** | Average *j* in SE | Standard Deviation of *j* | $j_{>3\sigma}$ | $\max(j)$ | area exceeding CCD at 0.1 mA/cm² (%) | area exceeding CCD at 0.5 mA/cm² (%) | area exceeding CCD at 3 mA/cm² (%) |
| --- | --- | --- | --- | --- | --- | --- | --- |
| 00hMill_000MPa | 1.494 | 0.957 | 7.123 | 21.71 | 0.03528 | 24.45 | 88.47 |
| 00hMill_200MPa | 1.252 | 0.557 | 4.259 | 10.29 | 0.00077 | 5.38 | 92.95 |
| 00hMill_600MPa | 1.262 | 0.645 | 5.097 | 11.32 | 0.00235 | 7.64 | 91.12 |
| 02hMill_000MPa | 1.771 | 1.183 | 9.230 | 20.82 | 0.09939 | 36.57 | 91.02 |
| 02hMill_200MPa | 1.500 | 0.895 | 5.881 | 28.01 | 0.00188 | 26.21 | 89.97 |
| 02hMill_600MPa | 1.204 | 0.437 | 3.481 | 18.00 | 0.00225 | 2.28 | 95.35 |
| 04hMill_000MPa | 1.573 | 1.082 | 8.595 | 16.61 | 0.06562 | 29.95 | 89.66 |
| 04hMill_200MPa | 1.410 | 0.897 | 7.501 | 25.27 | 0.05854 | 18.03 | 90.74 |
| 04hMill_600MPa | 1.484 | 0.933 | 7.480 | 14.96 | 0.03371 | 24.29 | 90.96 |
| 10hMill_000MPa | 1.445 | 0.859 | 5.703 | 27.04 | 0.03036 | 22.83 | 90.34 |
| 10hMill_200MPa | 1.347 | 0.712 | 4.671 | 19.39 | 0.00687 | 15.90 | 90.75 |
| 10hMill_600MPa | 1.474 | 0.983 | 7.211 | 15.85 | 0.01484 | 24.92 | 89.15 |

**Table S12.** Evaluation of normalized current densities obtained by the simulated conductivity measurements on the FIB-tomos. Data from the bottom interface layer of the tomographies is shown. The process and equations including an explanation of $j_{>3\sigma}$ are detailed in Section 4d) of the main manuscript and Note S4.

| **Data** | Average $j$ in SE | Standard Deviation of *j* | $j_{>3\sigma}$ | $\max(j)$ | area exceeding CCD at 0.1 mA/cm² (%) | area exceeding CCD at 0.5 mA/cm² (%) | area exceeding CCD at 3 mA/cm² (%) |
| --- | --- | --- | --- | --- | --- | --- | --- |
| 00hMill_000MPa | 1.612 | 1.258 | 10.092 | 30.83 | 0.14905 | 33.51 | 86.81 |
| 00hMill_200MPa | 1.347 | 0.807 | 6.423 | 12.55 | 0.00513 | 17.43 | 90.54 |
| 00hMill_600MPa | 1.197 | 0.566 | 4.217 | 15.42 | 0.00828 | 4.74 | 91.48 |
| 02hMill_000MPa | 1.575 | 1.055 | 8.516 | 20.79 | 0.05633 | 29.53 | 88.37 |
| 02hMill_200MPa | 1.455 | 0.955 | 6.435 | 17.53 | 0.00906 | 25.58 | 84.46 |
| 02hMill_600MPa | 1.155 | 0.458 | 3.228 | 9.50 | 0.00000 | 2.50 | 94.69 |
| 04hMill_000MPa | 1.404 | 0.841 | 5.363 | 9.50 | 0.00000 | 19.98 | 89.22 |
| 04hMill_200MPa | 1.377 | 0.919 | 6.745 | 15.11 | 0.04707 | 20.27 | 85.45 |
| 04hMill_600MPa | 1.290 | 0.643 | 4.457 | 12.01 | 0.00160 | 9.26 | 90.49 |
| 10hMill_000MPa | 1.416 | 0.865 | 5.946 | 15.69 | 0.02257 | 18.75 | 90.63 |
| 10hMill_200MPa | 1.315 | 0.686 | 5.128 | 14.83 | 0.00647 | 10.97 | 90.62 |
| 10hMill_600MPa | 1.198 | 0.487 | 3.374 | 6.42 | 0.00000 | 2.96 | 92.93 |

**References**

[1] A. Neumann, T. R. Hamann, T. Danner, S. Hein, K. Becker-Steinberger, E. Wachsman, A. Latz, *ACS Applied Energy Materials* **2021**, 4, 4786.

[2] A. Verma, H. Kawakami, H. Wada, A. Hirowatari, N. Ikeda, Y. Mizuno, T. Kotaka, K. Aotani, Y. Tabuchi, P. P. Mukherjee, *Cell Reports Physical Science* **2021**, 2.

[3] O. Ronneberger, P. Fischer, T. Brox, presented at *Medical image computing and computer-assisted intervention–MICCAI 2015: 18th international conference, Munich, Germany, October 5-9, 2015, proceedings, part III 18*, **2015**.

[4] Y.-T. Chen, M. Duquesnoy, D. H. Tan, J.-M. Doux, H. Yang, G. Deysher, P. Ridley, A. A. Franco, Y. S. Meng, Z. Chen, *ACS Energy Letters* **2021**, 6, 1639.

[5] Y.-G. Lee, S. Fujiki, C. Jung, N. Suzuki, N. Yashiro, R. Omoda, D.-S. Ko, T. Shiratsuchi, T. Sugimoto, S. Ryu, *Nature Energy* **2020**, 5, 299.

[6] C. Sedlmeier, T. Kutsch, R. Schuster, L. Hartmann, R. Bublitz, M. Tominac, M. Bohn, H. A. Gasteiger, *Journal of The Electrochemical Society* **2022**, 169, 070508.

[7] A. Tron, R. Hamid, N. Zhang, A. Paolella, P. Wulfert-Holzmann, V. Kolotygin, P. López-Aranguren, A. Beutl, *Journal of Energy Storage* **2023**, 66, 107480.

[8] A. Mills, G. Yang, W.-Y. Tsai, X. C. Chen, R. L. Sacci, B. L. Armstrong, D. T. Hallinan, J. Nanda, *Journal of the Electrochemical Society* **2023**, 170, 080513.
